# LKB1‐MARK2 signalling mediates lipopolysaccharide‐induced production of cytokines in mouse macrophages

**DOI:** 10.1111/jcmm.15710

**Published:** 2020-08-25

**Authors:** Jie Deng, Chunmei Wen, Xiangyu Ding, Xi Zhang, Guoqing Hou, Andong Liu, Hui Xu, Xuan Cao, Yongheng Bai

**Affiliations:** ^1^ Key Laboratory of Diagnosis and Treatment of Severe Hepato‐Pancreatic Diseases of Zhejiang Province The First Affiliated Hospital Wenzhou Medical University Wenzhou China; ^2^ School of Ophthalmology and Optometry and Eye Hospital Wenzhou Medical University Wenzhou China; ^3^ Department of Medical Genetics School of Basic Medicine Tongji Medical College Huazhong University of Science and Technology Wuhan China; ^4^ Ultrastructural Pathology Laboratory Department of Pathology School of Basic Medicine Tongji Medical College Huazhong University of Science and Technology Wuhan China; ^5^ Institute of Chronic Kidney Disease Wenzhou Medical University Wenzhou China

**Keywords:** cytokine, lipopolysaccharide, LKB1, MARK2, phosphorylation

## Abstract

Lipopolysaccharide (LPS) is an endotoxin involved in a number of acute and chronic inflammatory syndromes. Although LPS‐induced signalling has been extensively studied, there are still mysteries remaining to be revealed. In the current study, we used high‐throughput phosphoproteomics to profile LPS‐initiated signalling and aimed to find novel mediators. A total of 448 phosphoproteins with 765 phosphorylation sites were identified, and we further validated that the phosphorylation of MARK2 on T208 was important for the regulation on LPS‐induced CXCL15 (human IL‐8 homolog), IL‐1β, IL‐6 and TNF‐α release, in which LKB1 had a significant contribution. In summary, induction of cytokines by LPS in mouse macrophage is regulated by LKB1‐MARK2 signals. Our study provides new clues for further exploring the underlying mechanisms of LPS‐induced diseases, and new therapeutic approaches concerning bacterial infection may be derived from these findings.

## 1 INTRODUCTION

Lipopolysaccharide (LPS) is a bacterial endotoxin, part of outer membrane of cell wall of gram‐negative bacteria. The first host protein involved in the recognition of LPS is LPS‐binding protein (LBP), which brings LPS to the cell surface to form a ternary complex with CD14.^1^ Binding to CD14 facilitates LPS transfer to the LPS receptor complex composed of toll‐like receptor 4 (TLR4) and MD2, thereby transmitting the stimulus signal into the cells in a MYD88‐dependent or MYD88‐independent pathway.^2^ LPS activates macrophages to produce a variety of inflammatory cytokines, including interleukins (IL‐1β, IL‐6, IL‐8) and tumour necrosis factors (TNF‐α/β), which serve to trigger efficient immune response and protecting the host from bacterial infection.^3^ However, hyperactivity of immune response by severe bacterial infection leads to uncontrolled release of these substances, which may cause acute or chronic inflammatory syndromes.^4^ Although LPS‐related signalling has been extensively studied during the past decades, there are still mysteries and unknown signalling remaining to be revealed.

The MARK family (also known as Par‐1) is composed of four members including MARK1/Par‐1c, MARK2/Par‐1b/Emk, MARK3/Par‐1a/C‐Tak1 and MARK4/Par‐1d, which belong to the AMP‐activated protein kinase (AMPK)‐related protein kinases (also known as sucrose non‐fermenting 1, Snf1) of the Ca2+/calmodulin‐dependent kinase II (CaMK) group. MARK2 has been implicated in the regulation of a number of cellular processes, including generation and maintenance of cell polarity, metabolic rate, fertility, adiposity and insulin sensitivity.^5^ MARK2 is known to be activated by phosphorylation on T208 and inactivated by phosphorylation on S212, both of which are located in the activation loop of the catalytic domain,^6^ MARKK/TAO‐1 and LKB1 are known to be the upstream kinases.^7^, ^8^

LKB1, also known as Serine/threonine kinase 11 (STK11), is a master kinase that activates 13 kinases of the AMPK subfamily across evolutionary distant species.^8^ In addition to acting as a tumour suppressor, LKB1 also plays a role in regulating cell polarity and energy metabolism. LKB1‐mediated activation of MARKs has been reported to regulate microtubule dynamics. LKB1 has reported to phosphorylate and activate MARK2 at T208, which in turn phosphorylates microtubule‐associated protein Tau and suppresses tubulin polymerization.^9^, ^10^ However, the role of LKB1 and MARK2 in the immune response of macrophages to LPS remains unclear. In the current study, we conducted high‐throughput phosphoproteomics to profile LPS‐initiated signalling and further validated that the phosphorylation of MARK2 on T208 was important for the regulation on LPS‐induced CXCL15, IL‐1β, IL‐6 and TNF‐α release, in which LKB1 had a significant contribution. In summary, induction of cytokines by LPS in mouse macrophage is regulated by LKB1‐MARK2 signals.

## 2 MATERIALS AND METHODS

### 2.1 Cell culture

HeLa cells were obtained from American Type Culture Collection (Manassas, VA, USA). RAW264.7 cells were from the Cell Bank of Chinese Academy of Sciences (Shanghai, China). All cells were cultured in DMEM with 10% foetal bovine serum (Invitrogen, Carlsbad, CA, USA), 100 U/mL penicillin and 100 μg/mL streptomycin (Solarbio Science and Technology, Beijing, China). Cells were maintained in a humidified incubator at 37°C with 5% CO_2_.

### 2.2 Western blotting

Cells were lysed in a sample buffer containing 2% SDS, 60 mmol/L Tris‐HCl (pH 6.8) and 5% glycerol. Cell lysates were boiled for 5 minutes. Protein concentration was determined using a BCA kit (Beyotime, Shanghai, China), and equal amount of protein was loaded for Western blot analysis as previously described.^11^ Primary antibodies against ERK1/2, p‐ERK1/2, p38, p‐p38, JNK1/2, p‐JNK1/2, PKCδ, p‐PKCδ, PKA, p‐PKA, MARK2, p‐MAPK, LKB1, p65 and p‐p65, as well as secondary antibodies, were all from Cell Signaling Technology (Boston, MA, USA). Anti‐β‐actin antibody was from Proteintech (Wuhan, China). Blots were developed using enhanced ECL chemiluminescence reagents (Pierce, Thermo Scientific, Waltham, MA, USA) according to the manufacturer's instruction. β‐actin was used as a loading control.

### 2.3 Phosphopeptide preparation

RAW264.7 cells were collected with a lysis buffer containing 20 mmol/L Tris, 0.2 mmol/L EDTA and 8 mol/L urea within protease and phosphatase inhibitors (Bimake, Houston, USA). Proteins obtained above were then incubated with DTT and iodoacetamide for 12 hours digestion within trypsin at 37°C. StageTips were used for desalting the tryptic peptides. After that, the peptides were fractionated by hydrophilic interaction liquid chromatography (HILIC) as previously described.^12^, ^13^ All samples were performed on an Agilent 1100 HPLC system using a 1 × 250 mm TSKgel Amide‐80 5 µm particle column (Tosoh Biosciences, South San Francisco, CA, USA). The peptides were dried under vacuum followed by reconstitution within 5% trifluoroacetic acid (TFA)/60% acetonitrile. Phosphopeptides were enriched on TiO2 beads (GL Sciences, Inc, Tokyo, Japan) in StageTips.

### 2.4 Liquid chromatography‐mass spectrometry (LC‐MS)

A Thermo Scientific EASY‐nLC 1000 coupled to a Q Exactive mass spectrometer was used for LC‐MS. The peptide separation was performed by a reversed‐phase column (Reprosil C18, 3 µm, Dr Maisch GmbH, Ammerbuch, Germany). The Q Exactive was operated in top 10 data‐dependent mode with survey scans acquired at a resolution of 70 000 at *m*/*z* 200. MS/MS scans were set to a resolution of 17 500 at *m*/*z* 200. Spectra were acquired with a normalized collision energy of 27 eV and a dynamic exclusion duration of 30 seconds.^12^, ^13^

### 2.5 Mass spectrometric analysis

The macrophage files were identified and quantified using the MaxQuant computational proteomics platform. The fragmentation spectra were used to search the UniProt mouse protein database. Carbamidomethylation of cysteine was set as a fixed modification, and oxidation of methionine and protein N‐terminal acetylation, D_4_‐lysine, ^13^C_6_‐arginine, ^13^C_6_‐^15^N_2_‐lysine, and ^13^C_6_‐^15^N_4_‐arginine were used as variable modifications for database searching. Both protein and peptide identifications were filtered for <1% false discovery rate (FDR).

### 2.6 Plasmids, siRNA and reporter gene activity assay

The open reading frame of MARK2 and LKB1 was amplified according to the sequence of NM_007928.3 and NM_011492.4, respectively, and cloned into the pEGFP‐C3 vector (Invitrogen). MARK2 and LKB1 mutations were constructed using QuikChange II XL site‐directed mutagenesis kit (Stratagene, La Jolla, CA, USA). The pcDNA 3.1‐based expression vectors of TLR4, MD2 and CD14, as well as CXCL15‐driven firefly luciferase reporter, were previously kept in the laboratory. Renilla luciferase pRL‐TK was from Promega (Madison, WI, USA). All plasmids were prepared using Endofree Plasmid Purification kit (Qiagen, Valencia, CA, USA), and siRNAs were from GenePharma (Shanghai, China). Transfection was performed using Lipofectamine 2000 (Invitrogen) except for the transfection of plasmid into RAW264.7 cells, which used Lonza Nucleofector electrophoresis system. For the luciferase reporter assay, HeLa cells in 24‐well plate were transfected with siRNA overnight, followed by cotransfection with vectors of TLR4, MD2 and CD14, CXCL15‐luc and pRL‐TK.^14^ After 24 hours, the cells were stimulated with LPS (100 ng/mL) for designated time and measured for luciferase activity using the Dual‐Luciferase Reporter assay system (Promega).

### 2.7 Real‐time PCR (RT‐PCR)

Total RNA was isolated using an RNA extraction kit from Promega and was reversely transcribed to cDNA using RT Super Mix (Vazyme, Nanjing, China). Real‐time PCR was performed using SYBR qPCR Master Mix (Vazyme). Primer pairs were listed in Table S1. Levels of mRNA expression were presented after normalization to their respective β‐actin.

### 2.8 Cytokine measurement

Transfected RAW264.7 cells were stimulated with LPS (100 ng/mL) for 2 hours. Medium concentrations of CXCL15, IL‐1β, IL‐6 and TNF‐α were then measured using ELISA kits purchased from Thermo Fisher (Waltham, MA, USA) according to the manufacturer's instructions.

### 2.9 Statistical analysis

Data were analysed by one‐way analysis of variance (ANOVA) with Dunnett's test in GraphPad Prism 7. Values are expressed as means ± standard deviation (SD) from at least three independent experiments. Difference was considered statistically significant when *P* < 0.05.

## 3 RESULTS

### 3.1 LPS‐induced phosphorylation signalling in macrophages

**FIGURE 1:**
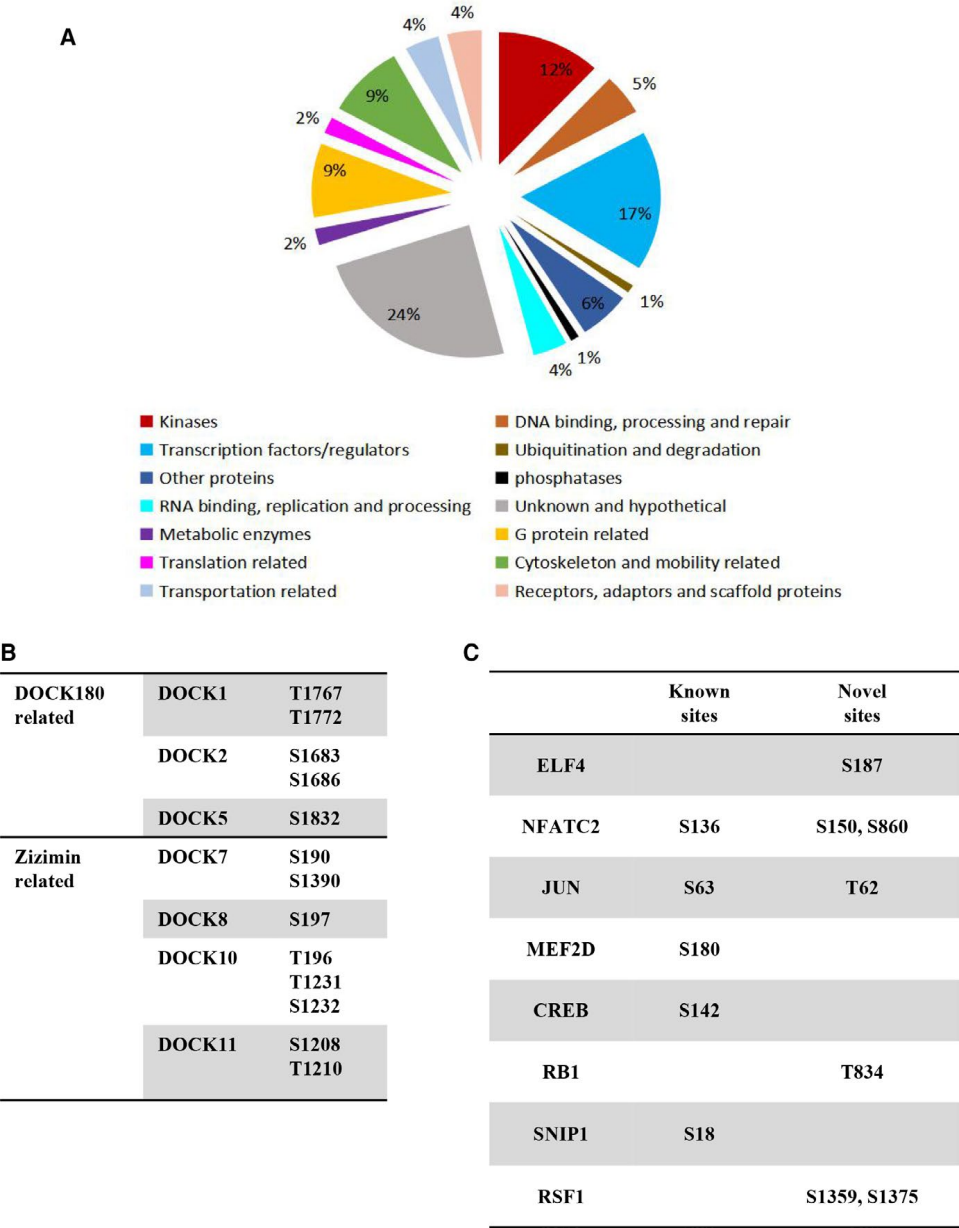
Macrophage phosphorylation sites and proteins involved in lipopolysaccharide stimulation. A, Functional classification of phosphorylated proteins. B, Classification and the phosphorylation sites of Dock family members. C, The phosphorylation sites of known transcription factors which are required for cytokines induction

Three established MAPKs, including ERK1/2, p38 and JNK1/2,^15^ were used a positive control to ensure the functioning of LPS stimulation in RAW264.7 cells (Figure S1A). The time‐point of 30 minutes was used to profile the phosphorylation signalling. A total of 765 phosphorylation sites originated from 448 proteins were identified (Table S2). Among these, proteins were 17% of transcription factor/regulator, 12% of kinases, 9% of G protein‐related proteins and 9% of cytoskeleton and mobility‐related proteins (Figure 1A). Noteworthy, a series of DOCK family proteins were detected with phosphorylation, including DOCK180‐related proteins (DOCK1, 2, and 5) and zizimin‐related proteins (DOCK7, 8, 10 and 11) (Figure 1B). In the identified phosphorylation sites of 63 transcription factors/regulators, 59 were known phosphorylation sites such as p‐JUN^S63^ and p‐NFATC2^S136^ whereas 39 sites were novel sites. According to the different protein domains, these transcription factors/regulators were classified to some protein families. The top three common families were FYVE/PHD zinc finger, winged‐helix DNA‐binding domain, and the classical C2H2 and C2HC zinc finger (Table 1). Identified transcription factors/regulators, which are known to interact with the transcription factors of TNF‐α, IL‐1β, IL‐6 and CXCL15, included CREBBP, RSF1, RB1, TAF1, GTF2I, and MEF2D, were also shown phosphorylated by LPS stimulation (Figure 1C). Among a total of 12 phosphorylation sites in these factors, 7 were novel sites.

**TABLE 1:** Classification of transcription factors related to lipopolysaccharide signalling pathway

Superfamily	Transcription factors
FYVE/PHD zinc finger	BRD1 (S128*), BRPF1 (S1074), JARID1A (S1111), MLLT6 (S340), MLLT10 (S370*, S436), PHF2 (S876), PHF3 (S660*), ZMYND8 (S401*, S524*), RSF1 (S1359*, S1375*)
Winged‐helix DNA‐binding domain	ELF4 (S187), ETV3 (S173*, S181*), FOXK1 (S229, S239), FOXM1 (S635*), FOXO3A (S299*), GTF2F1 (S385, S391), TFDP1 (S23)
C2H2 and C2HC zinc fingers	HIVEP3 (S2053*), PRDM2 (S777*), RREB1 (T739*), TRPS1 (S216), ZFP148 (S306*)
Helix‐loop‐helix DNA‐binding domain	MLX (S45, S48), TCF3 (T179*), TCFE3 (S553), TFAP4 (S120*)
Cyclin‐like	CCNT2 (S596), RB1 (T834), RBL1 (T332,T369,T384*,T385), RBL2 (S1076)
SRF‐like	MEF2C (S222), MEF2D (S180)
Tudor/PWWP/MBT	BRPF1, MBD5 (S246*), PRKCBP1
Bromodomain	BRD1, BRPF1, PRKCBP1
Homeodomain‐like	NCOR1 (S1274), ZHX1 (T197*, S207*)
Leucine zipper domain	CREB1 (S142), JUN (T62*, S63)
Rel/Dorsal DNA‐binding domain	NFATC1 (S247), NFATC2 (S136, S860)
p53‐like transcription factors	NFATC1, NFATC2
P‐loop containing nucleoside triphosphate hydrolases	ATRX (S588*, S590), CHD8 (S2520)
CTF/NF‐I family transcription modulation region	NFIC (S323, S339, S343), NFIX (S280, S284*, S301, S340*, S341)
SMAD MH1 domain	NFIC, NFIX
SMAD/FHA domain	FOXK1, SNIP1 (S18)
DNA‐binding domains of HMG‐I(Y)	AHCTF1 (S1541, S1940*, T1954*), APRIN (S1381)
SNF2 family N‐terminal domain	ATRX, CHD8
Glucocorticoid receptor‐like (DNA‐binding domain)	GATAD2B (S487), TRPS1
N/A	PPARBP (S664, S772*, T805, S1049*, T1051, T1057, S1435, S1441*, T1442), THRAP4 (S862*)
Other domains	APRIN, ARID1A (S697), CHD8, CIC (S766*, S1809*), CREB1, JARID1A, MBD5, MLL1 (S151), MLL2 (S2231, S2299, S4410), NAB2 (S171), NFKBIL2 (S873*), PHF2, PHF3, PRDM2, PRKCBP1, SON (S1723), SUPT5H (S664, T822*), TAX1BP1 (S693), TFDP1, TLE4 (S292), TNFAIP3 (T161*, S577*), ZDHHC5 (S621, S693*, T696*), ZFP318 (S246)

Note

Among the transcription factors/regulators, 59 are known phosphorylation sites and 39 novel phosphorylation sites (*).

### 3.2 Analysis of the LPS‐induced phosphorylation of kinases and phosphatases

**FIGURE 2:**
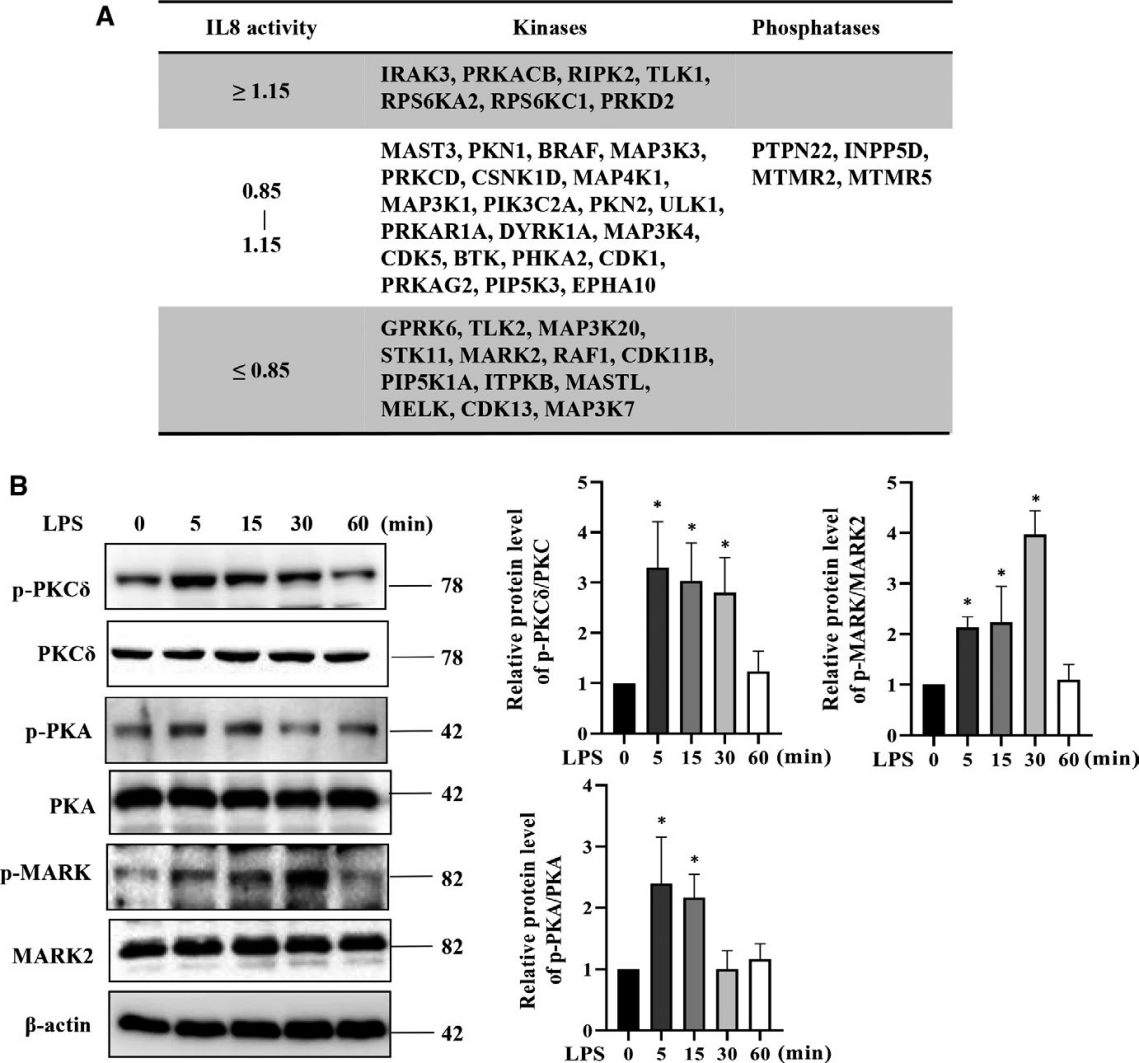
Lipopolysaccharide (LPS)‐induced phosphorylation of kinase and phosphatases. A, Hela cells were transfected with reporter gene vectors and the indicated siRNA for 48 h, followed by LPS (100 ng/mL) for 30 min, and the CXCL15 transcriptional activity was determined by luciferase reporter assay. B, RAW264.7 cells were stimulated with LPS (100 ng/mL) for 30 min, and the phosphorylation of PKCδ, PKA and MARK2 was determined by Western blotting. All data are presented as means ± SD of three independent experiments. *P < 0.05 compared with control group

Lipopolysaccharide stimulation led to phosphorylation in a total of 41 kinases and 4 phosphatases. Among total 87 phosphorylation sites, 31 sites were found to be novel (Table 2). In order to investigate whether these kinases or phosphatases were involved in LPS‐induced cytokine release, we knocked down most of them individually with siRNA available in engineered HeLa cells for CXCL15 luciferase reporter assay. Results showed that 7 kinases upon knockdown led to an increase of CXCL15 production by 15%, whereas 13 kinases led to a reduction by 15% (Figure 2A; Tables S3 and S4). Based on the availability of antibody and the novelty, MARK2 (microtubule affinity regulating kinase 2), which has not been previously associated with LPS stimulation, was selected for further study. Two phosphorylation sites, that is p‐MARK2^T208^ and p‐MARK2^S453^, were identified in the dataset (Table 2). The p‐PKA^T197^ and p‐PKCδ^T505^, two known sites stimulated by LPS^16^ and also identified in the current study (Table S2), were used as positive controls. Results of Western blot indeed showed that p‐MARK2^T208^ was stimulated by LPS in a time‐dependent manner. The peak time was at 30 minutes following LPS stimulation in contrast with 5 minutes for p‐PKA^T197^ and p‐PKC^T505^ (Figure 2B; Figure S2A).

**TABLE 2:** Lipopolysaccharide‐induced activation of kinases and phosphatases

Kinases	Phosphatases
MAP3K3 (S166, S316*, S355*), PIP5K1A (S421*, S445, S447, S448), RAF1 (S29), CDK1 (Y15), PRKAG2 (S113*, S196), MAP4K1 (S455*), RIPK2 (S414*), MASTL (T206, T221, Y586*), PRKCD (T505), PRKACB (T198), GPRK6 (S484), MAP3K20 (S567, S568*, S599, S634, S649, S650), RPS6KC1 (S196*, S280*, S281, T283*, S567*, S576*, S577, S779*), CDK5 (T17), PIK3C2A (S261), IRAK3 (S523*, S525*), MARK2 (T208, S453), CDK13 (S384), MAP3K4 (S492), PRKAR1A (S83), PRKD2 (S211), MELK (S521), ITPKB (S42*, S125*, S247*), ULK1 (T635), RPS6KA2 (S218, S377, S382*), PKN1 (S536, S540, S920), MAST3 (S731*, S732, S733*), MAP3K7 (S412, T417*), DYRK1A (Y321), PIP5K3 (S305*), TLK1 (S158*, S159), PHKA2 (S729), BTK (Y40), CSNK1D (S383, S384), PKN2 (T819*), STK11 (S31), MAP3K1 (S287), CDK11B (S270, Y583, T584), TLK2 (T98*, S99), BRAF (S787), EPHA10 (S471*, S473*)	PTPN22 (S634), INPP5D (S935, T964, S972), MTMR2 (S6, S58), MTMR5 (T1137, S1748*, T1749*)

Note

Among the kinases and phosphatases, 56 are known phosphorylation sites and 31 novel phosphorylation sites (*).

### 3.3 MARK2 phosphorylation on LPS‐induced CXCL15 production

**FIGURE 3:**
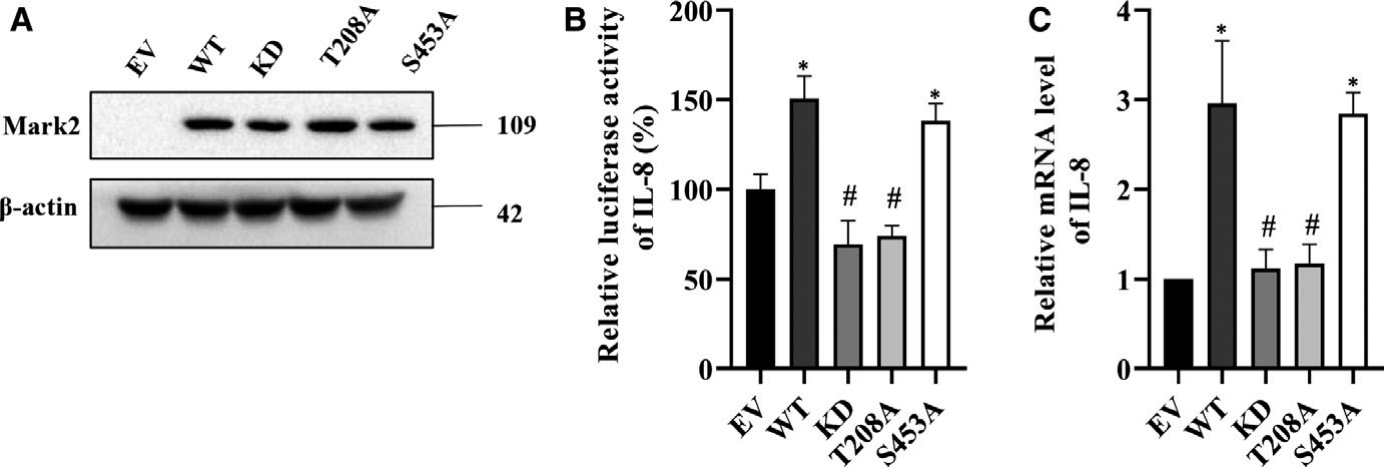
MARK2 phosphorylation on lipopolysaccharide (LPS)‐induced CXCL15 expression. A, RAW264.7 cells were transfected with empty vector (EV), wild‐type MARK2 (WT) and its kinase‐dead mutant MARK2^T208A/S212A^ (KD), MARK2^T208A^ (T208A) and MARK2^S453A^ (S453A), respectively, for 48 h, and the protein levels of MARK2 were detected by Western blotting. B, Hela cells were transfected with reporter gene vectors, and the vectors mentioned in A, followed by LPS (100 ng/mL) incubation for 30 min, and the CXCL15 transcriptional activity was determined by luciferase reporter assay. C, RAW264.7 cells were transfected with the vectors mentioned in A for 48 h, followed by LPS (100 ng/mL) incubation for 30 min, and the mRNA level of CXCL15 was detected by RT‐PCR. All data are presented as means ± SD of three independent experiments. *P < 0.05 compared with EV group, #*P* < 0.05 compared with WT group

MARK2 is known to be activated by phosphorylation on T208 and inactivated by phosphorylation on S212, both of which are located in the activation loop of the catalytic domain.^6^ To understand the function of the above identified p‐MARK2^T208^ and p‐MARK2^S453^ on LPS‐induced cytokine production, we constructed expression vectors of wild‐type MARK2 (MARK2^WT^), and its mutants MARK2^T208A^, MARK2^S453A^ and the kinase‐dead MARK2^T208A/S212A^ (Figure 3A; Figure S2B). After transfection into RAW264.7 cells and LPS stimulation, CXCL15 luciferase activity was elevated by MARK2^WT^ (Figure 3B), which is in line with the previous siRNA results (Figure 2A; Table S3). MARK2^S453A^ exhibited similar effect to MARK^WT^, indicating p‐MARK2^S453^ may not be functional. In contrast, both MARK2^T208A^ and MARK2^T208A/S212A^ suppressed the CXCL15 activity (Figure 3B). This effect was further confirmed by RT‐PCR analysis of CXCL15 mRNA level (Figure 3C).

### 3.4 LKB1 and its phosphorylation on the activation of MARK2

**FIGURE 4:**
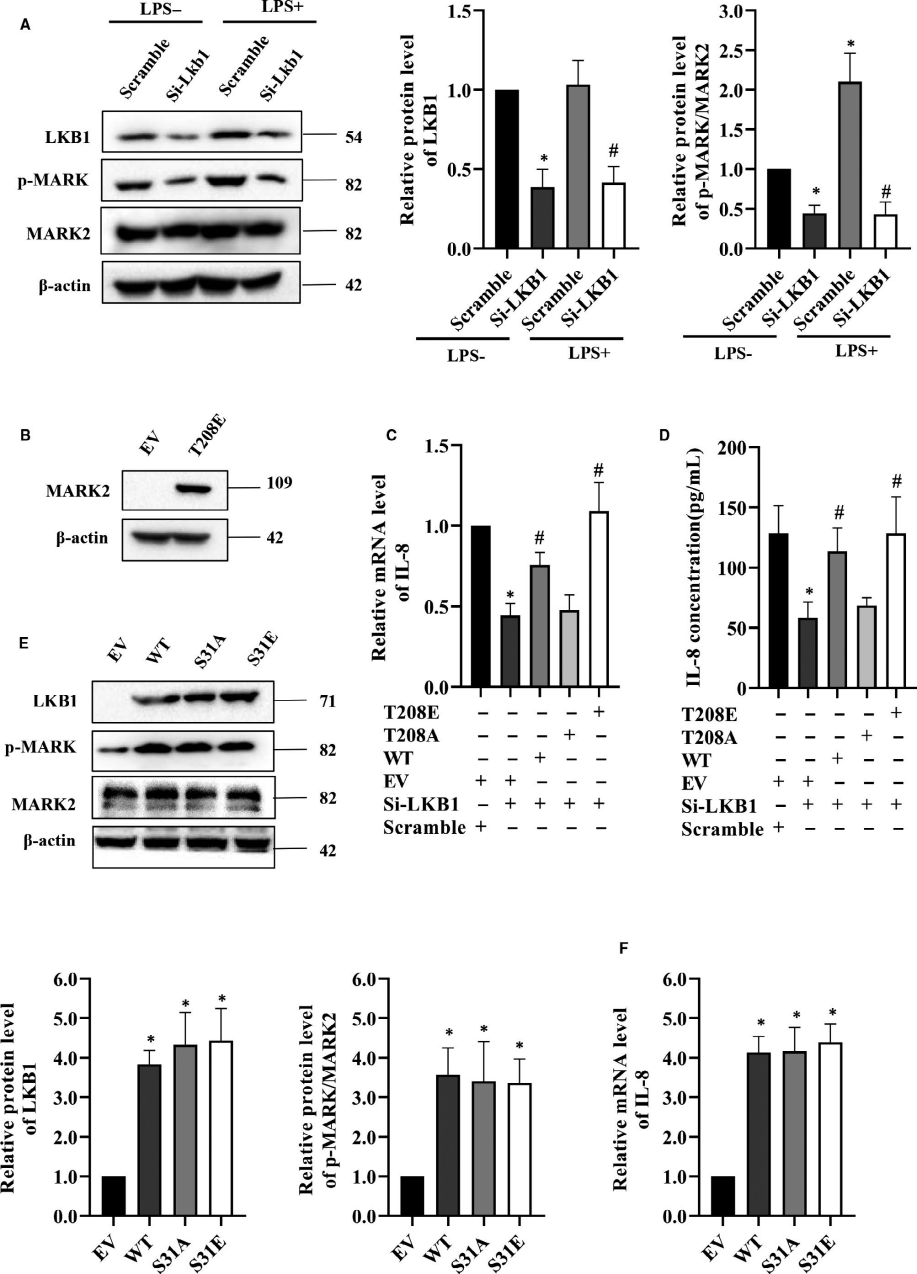
LKB1 and its phosphorylation on the activation of MARK2 and CXCL15 production. A, RAW264.7 cells were transfected with si‐LKB1 for 48 h, followed by DMSO or lipopolysaccharide (LPS) (100 ng/mL) stimulation for 30 min, and the protein level of LKB1 and the activation of MARK2 were determined by Western blotting. *P < 0.05 compared with scramble group, #*P* < 0.05 compared with Si‐LKB1 group. B, Western blotting analysis of the protein level of MARK2 whereas overexpressing empty vector (EV) and MARK2^T208E^ (T208E) in RAW264.7 cells. C, LKB1‐silenced RAW264.7 cells were transfected with EV, wild‐type MARK2 (WT), MARK2^T208A^ (T208A) and MARK2^T208E^ (T208E) for 48 h, followed by LPS (100 ng/mL) incubation for 30 min, and the mRNA level of CXCL15 was detected by RT‐PCR. *P < 0.05 compared with scramble + EV group, #*P* < 0.05 compared with si‐LKB1 + EV group. D, The production of CXCL15 in the supernatant was determined by ELISA under the treatment as C described. *P < 0.05 compared with scramble + EV group, #*P* < 0.05 compared with si‐LKB1 + EV group. E, RAW264.7 cells were transfected with EV, wild‐type LKB1 (WT) and its mutants LKB1^S31A^ (S31A) and LKB1^S31E^ (S31E) for 48 h, followed by LPS (100 ng/mL) stimulation for 30 min, and the protein level of LKB1 and the activation of MARK2 were determined by Western blotting. *P < 0.05 compared with EV group. F, Real‐time PCR analysis of the mRNA level of CXCL15 in RAW264.7 cells under the treatment as E described. *P < 0.05 compared with EV group. All data are presented as means ± SD of three independent experiments

LKB1 is located at the upstream of MARK2 activation by stimulating p‐MARK2^T208^.^9^, ^10^ Silencing LKB1 expression indeed led to a reduction in levels of p‐MARK2^T208^ with or without LPS stimulation (Figure 4A; Figure S2C). As earlier noted, LKB1 siRNA knockdown also reduced LPS‐stimulated CXCL15 activity (Figure 2A; Table S3). To further understand whether this LKB1‐mediated reduction is via MARK2, we additionally constructed a kinase‐active mutant MARK2^T208E^ (Figure 4B; Figure S2D). After transfection with the expression vectors and LPS stimulation, results of both CXCL15 mRNA expression (Figure 4C) and production (Figure 4D) confirmed that LKB1 regulated LPS‐induced cytokine expression via MARK phosphorylation at T208, that is p‐MARK2^T208^.

In our dataset, LKB1 was phosphorylated at S31 after LPS stimulation (Table 2; Table S2), instead of its well‐known S428.^8^ This site appears to be conserved across species (Figure S1B). To understand whether this p‐LKB1^S31^ is functional and activates MARK2^T208^, we constructed expression vectors of wild‐type LKB1 (LKB1^WT^) and its putative kinase‐dead and kinase‐active mutants, LKB1^S31A^ and LKB1^S31E^. Unfortunately, results after transfection showed that p‐LKB1^S31^ had no impact on the activation of MARK2^T208^ (Figure 4E; Figure S2E), nor on the mRNA expression and cytokine production of CXCL15 (Figure 4F).

### 3.5 LKB1‐MARK2 axis mediates LPS‐induced cytokines production

**FIGURE 5:**
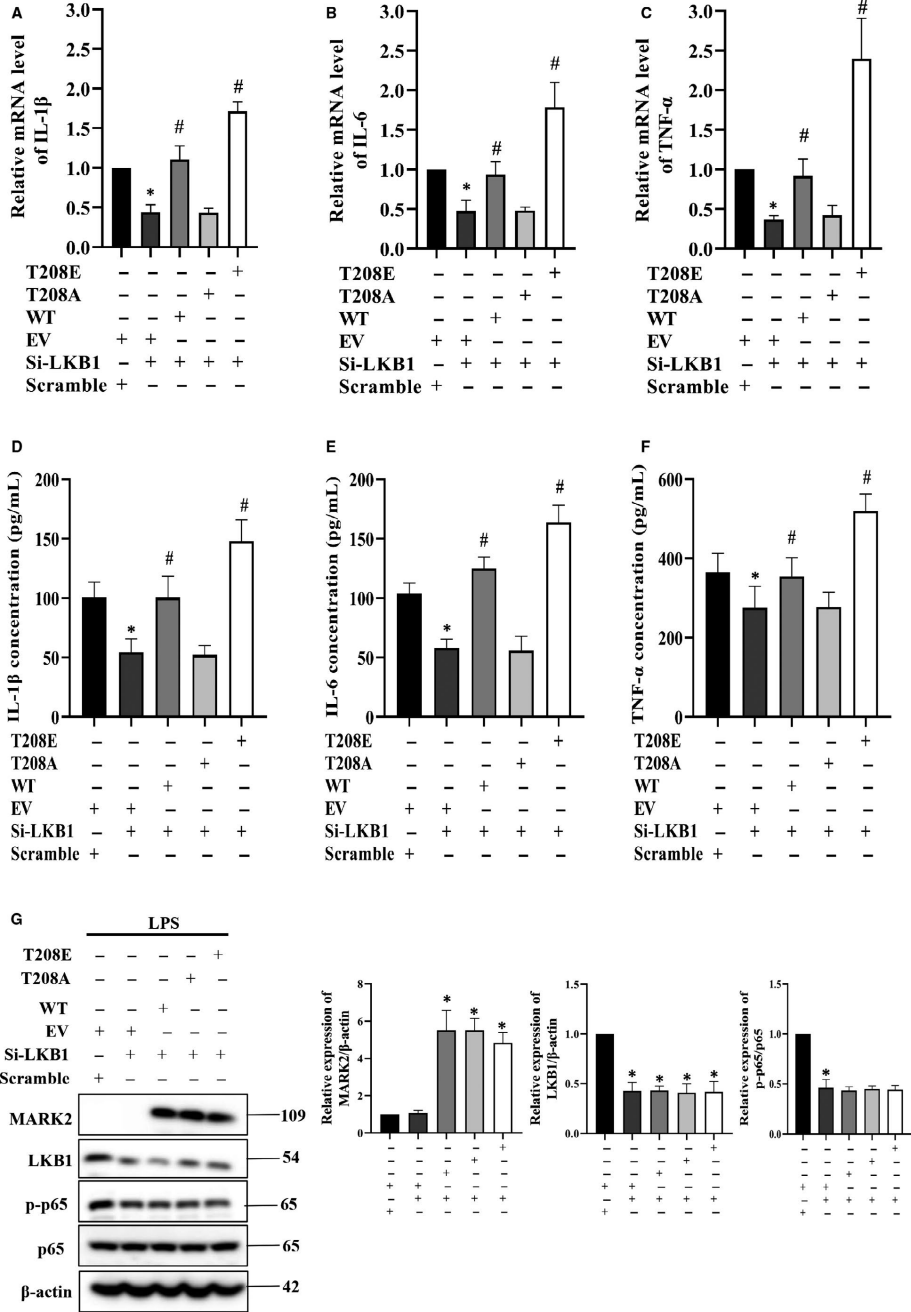
LKB1‐MARK2 mediates lipopolysaccharide (LPS)‐induced cytokines expression. (A‐C) LKB1 silenced RAW264.7 cells were transfected with empty vector (EV), wild‐type MARK2 (WT), MARK2^T208A^ (T208A) and MARK2^T208E^ (T208E) for 48 h, followed by LPS (100 ng/mL) incubation for 30 min, and the mRNA levels of IL‐1β (A), IL‐6 (B), and TNF‐α (C) were detected by RT‐PCR. (D‐F) The production of IL‐1β (D), IL‐6 (E), and TNF‐α (F) in the supernatant was determined by ELISA under the treatment as A‐C described. (G) MARK2, LKB1 and phosphorylation of p65 were determined by Western blotting under the treatment as A‐C described. All data are presented as means ± SD of three independent experiments. *P < 0.05 compared with scramble + EV group, #*P* < 0.05 compared with si‐LKB1 + EV group

As we found, LKB1‐MARK2 was involved in LPS‐induced CXCL15 production. We also evaluated the effects of LKB1‐MARK2 on IL‐1β, IL‐6 and TNF‐α production. As depicted in Figure 5, RT‐PCR results showed that LPS‐induced IL‐1β (Figure 5A), IL‐6 (Figure 5B) and TNF‐α (Figure 5C) mRNA levels were decreased after LKB1 knockdown, and this effect could be rescued by MAKR2^WT^ or MARK2^T208E^ overexpression but not by MARK2^T208A^. This effect was further confirmed by the measurement of IL‐1β, IL‐6 and TNF‐α secretion. Similarly, LKB1 knockdown could significantly lower LPS‐induced IL‐1β (Figure 5D), IL‐6 (Figure 5E) and TNF‐α (Figure 5F) production, which could be rescued by MAKR2^WT^ or MARK2^T208E^ overexpression but not by MARK2^T208A^. Collectively, it indicated that LPS‐induced cytokines production is regulated by LKB1‐MARK2 signals via MARK phosphorylation at T208. NF‐κB is involved in LPS‐induced expression of a serial of pro‐inflammatory cytokines. To elucidate whether NF‐κB participated in LKB1‐MARK2 mediated cytokines production in response to LPS, we tested whether LKB1‐MARK2 had an effect on p‐p65. It showed that LKB1 knockdown could significantly lower LPS‐induced phosphorylation of p65, with no disturbance of p65, MAKR2^WT^ overexpression had no effect on those of LKB1 silenced, so did MARK2^T208E^ and MARK2^T208A^ (Figure 5G; Figure S2F). This indicated that LKB1‐regulated phosphorylation of p65 in response to LPS is most probably independent of MARK2.

## 4 DISCUSSION

**FIGURE 6:**
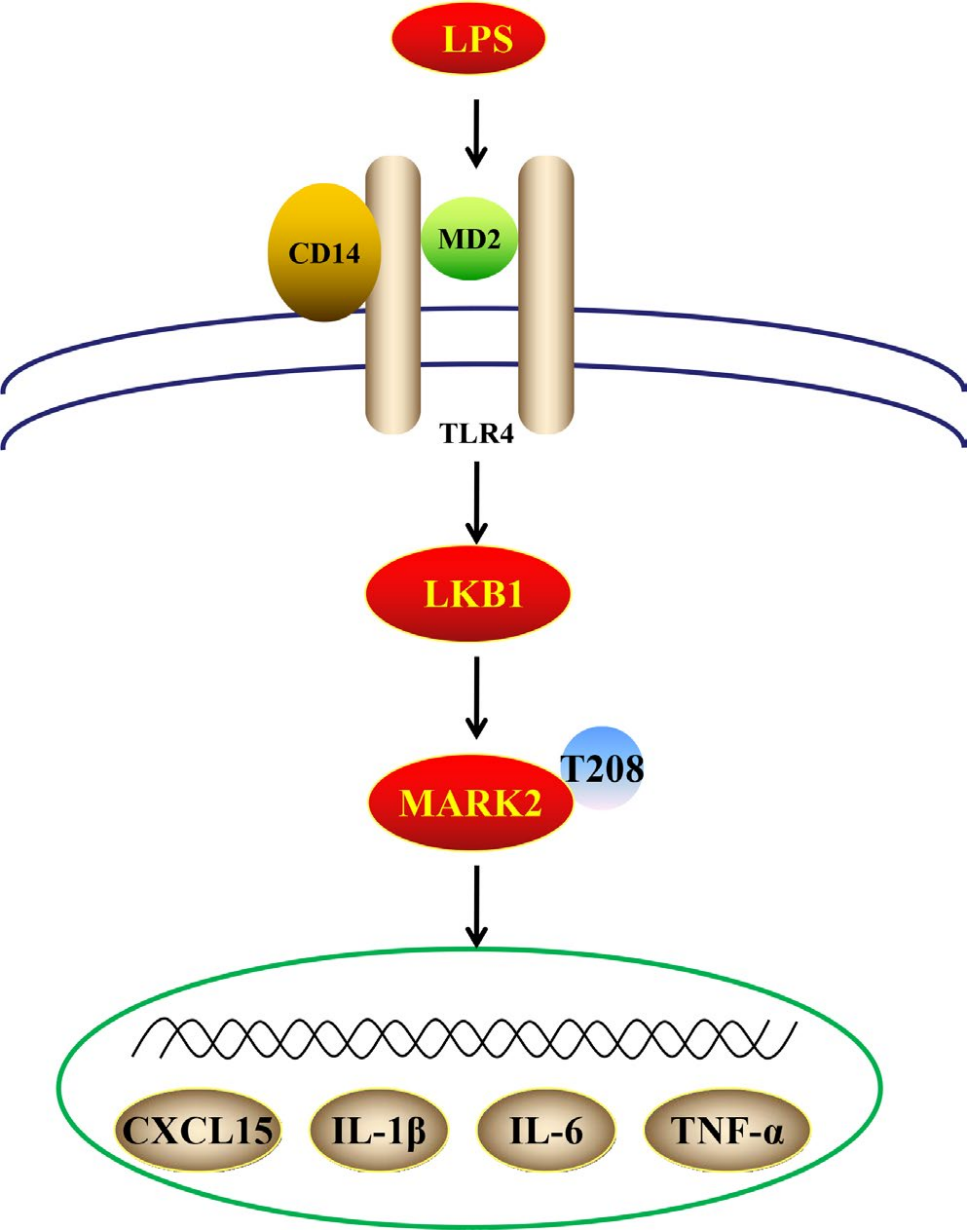
Schematic of the proposed model in this study. LKB1 suppressed lipopolysaccharide‐induced cytokines secretion via phosphorylation of MARK2 at T208

In this study, a total of 448 phosphoproteins with 765 phosphorylation sites were identified in LPS‐stimulated macrophages, with a majority of phosphorylation sites in transcription factors/regulators and kinase. Starting from the profiling data and subsequent CXCL15 reporter screening, we demonstrate that two novel kinases, MARK2 and LKB1, are involved in LPS‐induced cytokine production (Figure 6).

Proteins from DOCK family are large polypeptides, which function as guanine nucleotide exchange factors (GEFs), experimentally proven or potentially. Most members identified have two domains, CZH1 and CZH2 domains, whereas some contain third domains such as PH or SH3 domain. Experimental evidences have linked CZH2 domain to activation of Rho proteins as a GEF activity domain.^17^ CZH1 always precedes CZH2, and these two domains may be functionally linked such as inhibition of activity by domain interaction.^18^ The DOCK family has been shown involved in multiple biological processes such as phagocytosis, cell migration and regulation of cytokine production, mostly by activating small GTPases.^19^ Based on domain structure, sequence similarity and phylogenetic analysis, our data identified multiple novel phosphorylation sites of DOCK1, 2, 5, 7, 8, 10 and 11 following LPS stimulation. Noteworthy, all of the phosphorylation sites of DOCK1‐related proteins were located behind CZH2 domain. In contrast, all of the phosphorylation sites of zizimin‐related fell ahead of CZH1 or between CZH1 and CZH2 domains.^20^ Although the biological function of these phosphorylation sites is still under investigation, it most likely affects the binding of their substrates, and CZH1 and CZH2 interaction.

The transcription factors have been shown involved in LPS‐induced transcriptional activation and cytokine production.^21^ Consistent with previous reports, we found proteins such as JUN, CREB1 and MEFs were phosphorylated following LPS stimulation.^22^ However, most of factors/regulators identified to be phosphorylated were the first time linked to the signalling cascade stimulated by LPS. The amount of new transcription factors/regulators and the novel phosphorylation sites indicated LPS‐induced transcriptional regulation is much more complicated than what we have known.

Kinase and phosphatase are involved in variety of signalling cascade initiated by all kinds of stimuli. As expected, LPS stimulation led to phosphorylations in a number of kinases or phosphatases. Kinases, such as MAP3K7/TAK1 and BTK identified in this study, have been reported previously to be involved in LPS‐induced signalling cascade.^23^, ^24^ Besides, MARK2^T208^ was the first time to be lined with LPS‐induced signal pathway. Although the phospho‐MARK antibody could not distinguish MARK2 T208 from MARK1S215 and MARK3S234, MARK2 was most likely the major MARK protein phosphorylated by LPS treatment combined with our MS data as well as a report described before. The fact that only MARK2 was phosphorylated among MARK family after LPS stimulation raised a question that how did LPS achieve such differential regulation of MARK members in the same family. It was noted that MARK2 T208 and S453 were not identified phosphorylated after LPS treatment in an earlier study concerning primary macrophages, in which sites were replaced by the other 8 S/T points.^22^ This might be due to the difference between cell types and the way that phosphorylated peptides were engaged. It was particularly worthy of note here that most phosphorylation sites were on serine and threonine residues, whereas tyrosine phosphorylation occurred only in 3% of the cases, which was in accordance with a previous report.^22^ This might be attributed to the much stronger enrichment ability of TiO2 beads in p‐S/T than that of p‐Y, along with the unequal distribution of the number of serine/threonine kinases and tyrosine kinases.^25^

Both our MS data and an earlier report^22^ had identified LKB1 phosphorylation on S31 after LPS administration, but it might not be responsible for MARK2^T208^ mediated LPS‐induced cytokines production. It was reported that multiple residues on LKB1 were phosphorylated either by auto‐phosphorylation (Thr185, Thr189, Thr336 and Ser404) or phosphorylation by upstream kinases (Ser31, Ser325, Thr366 and Ser431).^26^ Both of which and the residues surrounding them were highly conserved. To date, the upstream kinases which were responsible for the phosphorylation of LKB1 at Ser31 had not been described yet. Ser31 lied in a consensus motif that might be phosphorylated by AMPK,^27^, ^28^ and it seemed to be necessary to validate whether AMPK or any other AMPK family members could also act as the upstream of LKB1. LKB1^S31^ was reported to be phosphorylated in HEK‐293 cells, which neither affected its nuclear localization nor catalytic activity in vitro.^27^, ^29^ It had been reported that LKB1 was not activated by phosphorylation of its activation loop, but was instead activated upon forming a complex of LKB1‐STRAD‐MD25,^29^ and it was probable that their interaction led to a conformational change which stabilized LKB1 in an active conformation. Combined with our data, LKB1^S31^ might not be required for the formation of LKB1‐STRAD‐MD25 complex, but further investigations needed to be carried out to confirm it. And other regulatory mechanisms might have existed among the pathway that regulating LPS‐induced CXCL15 release which was independent on LKB1^S31^ phosphorylation.

TAOK1 kinase has also been implicated as an upstream regulator that can phosphorylate the T208 residue of MARK2.^7^ Different from our MS finding, TAOK1 and TAOK3 were both to be identified phosphorylated at several sites in a study focusing on primary macrophages. However, TAOK1 only initiated MARK2 activity many times less than that of LKB1,^7^ and as mice cells possessed three closely related TAOK isoforms, the complex regulatory relationship between TAOK and MARK remained to be further explored. Lack of specific immunoprecipitating antibodies against T‐loop of different MARK members meant that it might be difficult to establish how did either TAOK or LKB1 affect the individual activities among MARK family.

It had been reported that LKB1^S428^ could interact with IKKβ in response to LPS stimulation, thus leading to the suppression of NF‐κB activation accompanied by a reduction of pro‐inflammatory cytokines.^30^ Different from our results, we showed that LKB1 deficiency could suppress LPS‐induced p65 phosphorylation and cytokines secretion. The reason might be attributed to LKB1^S428^ phosphorylation. Neither our MS data nor a previous report^22^ had found LKB1^S428^ phosphorylation; instead, LKB1^S31^ was identified. The reason why LPS initiates such disparate phosphorylation of LKB1 and the subsequent effects need further exploration.

## CONFLICT OF INTEREST

The authors confirm that there are no conflicts of interest.

## AUTHOR CONTRIBUTION

**Jie Deng:** Data curation (equal); formal analysis (equal); investigation (equal); methodology (equal); writing‐original draft (equal); writing – review and editing (equal). **Chunmei Wen:** Methodology (equal). **Xiangyu Ding:** Methodology (equal). **Xi Zhang:** Methodology (equal). **Guoqing Hou:** Methodology (equal). **Andong Liu:** Data curation (equal); formal analysis (equal). **Hui Xu:** Investigation (equal); methodology (equal). **Xuan Cao:** Conceptualization (equal); project administration (equal); supervision (equal). **Yongheng Bai:** conceptualization (equal); funding acquisition (equal); project administration (equal); resources (equal); supervision (equal); writing‐original draft (equal); writing – review and editing (equal).

## Supporting information

Fig S1

Fig S2

Table S1

Table S2

Table S3

Table S4

## Data Availability

All data generated or analysed during this study are included in this article.
