# Supplementary figures and images for: LKB1‐MARK2 signalling mediates lipopolysaccharide‐induced production of cytokines in mouse macrophages
Source: J Cell Mol Med. 2020 Aug 25;24(19):11307–17. doi: 10.1111/jcmm.15710 (PMC7576310; doi:10.1111/jcmm.15710)

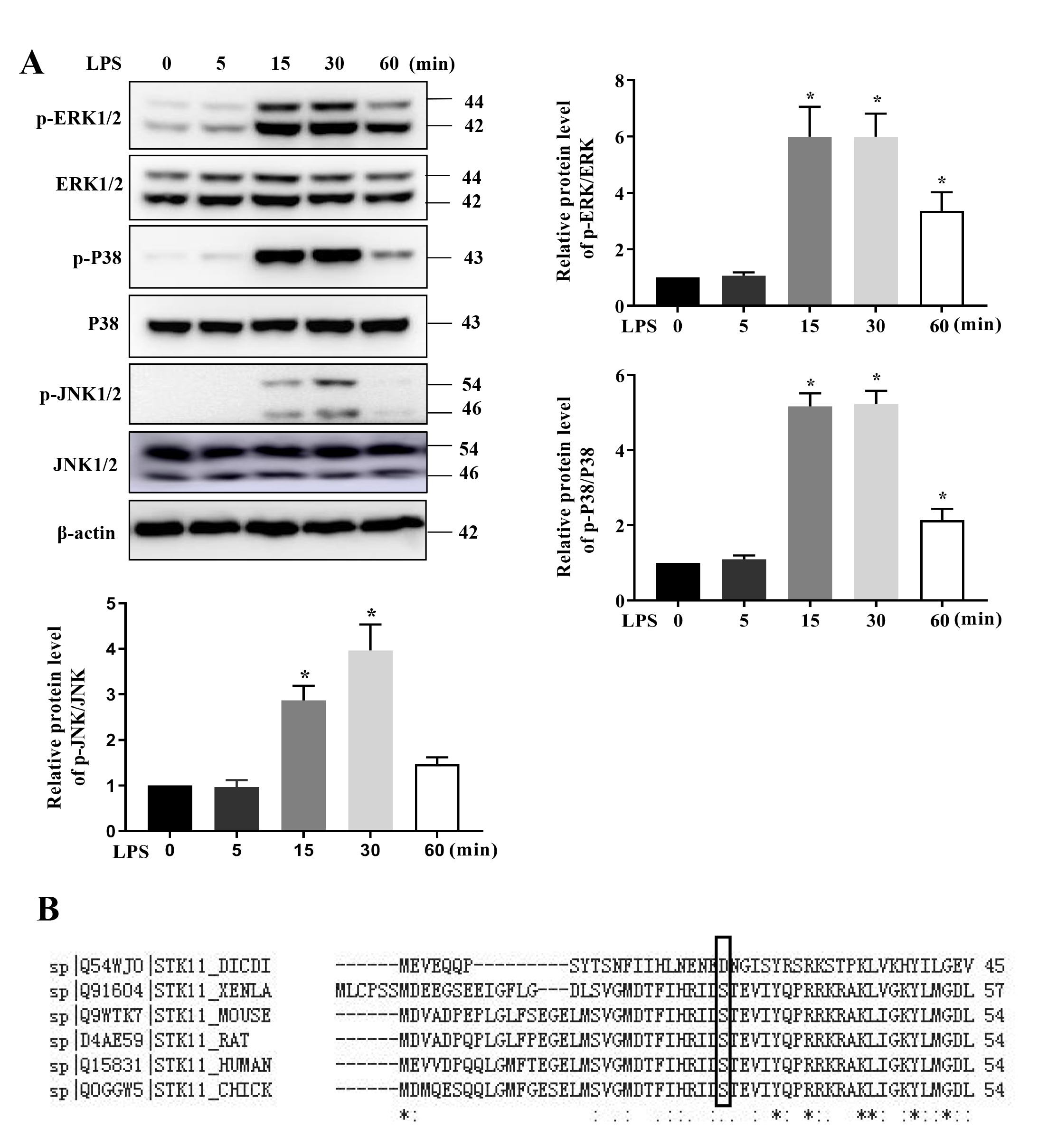

Supplement: Supplementary file 1 — Fig S1 [file JCMM-24-11307-s001.tif]

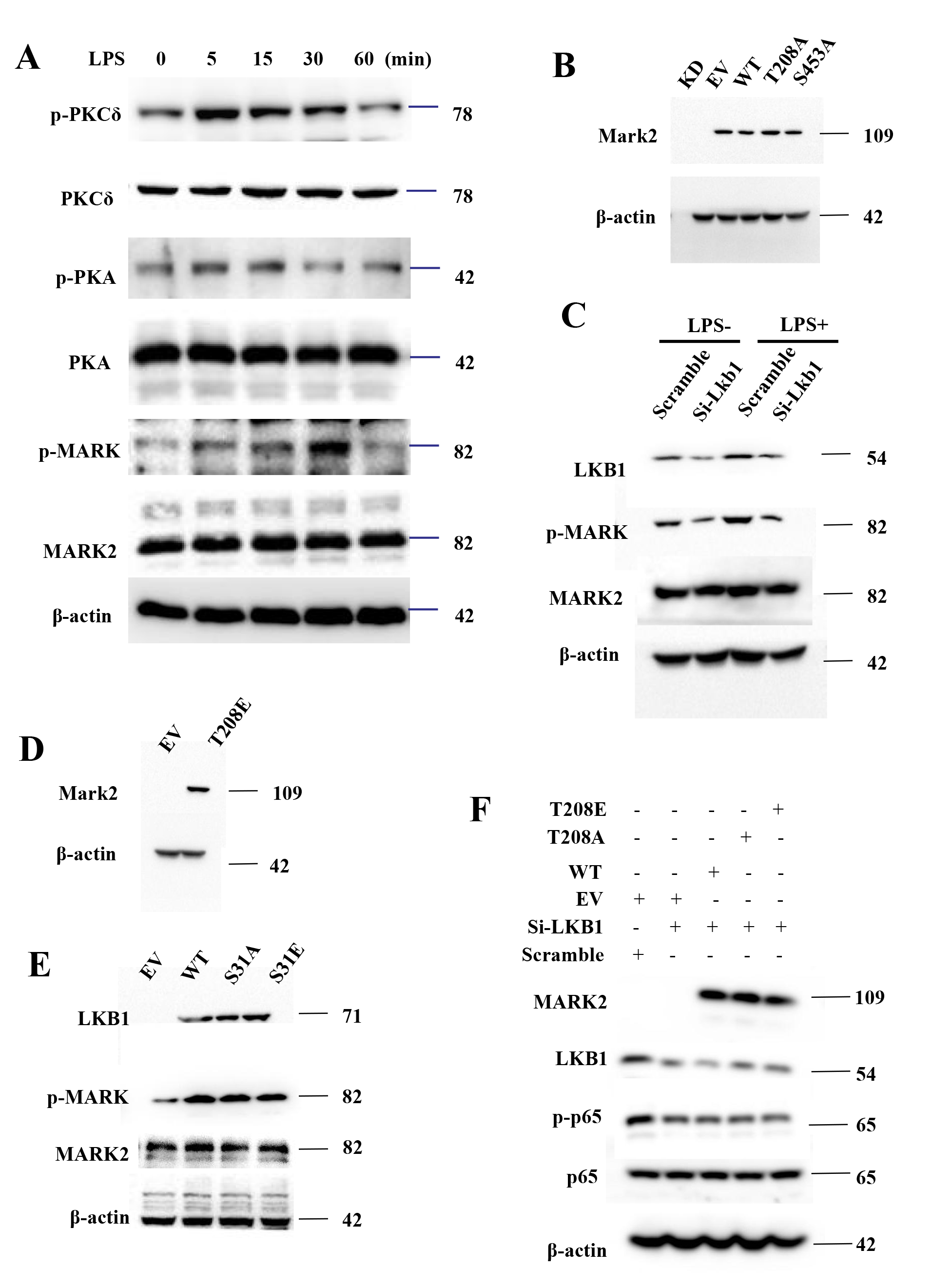

Supplement: Supplementary file 2 — Fig S2 [file JCMM-24-11307-s002.tif]
