# Supplementary material for: LKB1‐MARK2 signalling mediates lipopolysaccharide‐induced production of cytokines in mouse macrophages
Source: J Cell Mol Med. 2020 Aug 25;24(19):11307–17. doi: 10.1111/jcmm.15710 (PMC7576310; doi:10.1111/jcmm.15710)
Supplement: Supplementary file 3 — Table S1 [file JCMM-24-11307-s003.doc]

**Supplemental Table 1. Primers for RT-PCR analysis in this study**

| **Gene** | **Primers** |
| --- | --- |

| Irak3 | F: 5’-CTGGCTGGATGTTCGTCATATT-3’ |
| --- | --- |
|  | R: 5’-GGAGAACCTCTAAAAGGTCGC-3’ |
| Prkacb | F: 5’-AGATCGTCCTGACCTTTGAGT-3’ |
|  | R: 5’-GGCAAAACCGAAGTCTGTCAC-3’ |
| Ripk2 | F: 5’-ATCCCGTACCACAAGCTCG-3’ |
|  | R: 5-GGATGTGTAGGTGCTTCACTG-3’ |
| Tlk1 | F: 5’-AGTCAGGGAAAAAGTATCGGGG-3’ |
|  | R:5’-TTCTGCGGAGAACGGATTGC-3’ |
| Tlk2 | F: 5’-GGCTACTGAAGTACAGTTCCCG-3’ |
|  | R: 5’-ATCAATGCGATCTTCCTTTCGAT-3’ |
| Rps6ka2 | F:5’-GTGTCCCCTTGGCGAATTAAG-3’ |
|  | R: 5’-GTACACTCAGTAGCTCAAAGCAG-3’ |
| Rps6kc1 | F: 5’-AGGACTGCGGAGTACCTCAT-3’ |
|  | R: 5’-GGGCCTTGAACTTAGTGATCC-3’ |
| Mastl | F: 5’-TCGGCAAGTGAGGAGAATGAA-3’ |
|  | R: 5’-CACCACGGCTAATGGGCTT-3’ |
| Mast3 | F:5’-AGGCTGCATCTATCAGAGCG-3’ |
|  | R: 5’-AGGCTCCTCATCGAAGCTCA-3’ |
| Mtmr2 | F: 5’-AGAACTCGGTGCATACCAAATC-3’ |
|  | R: 5’-GCTAACTTGTTTGCCTCCCTCA-3’ |
| Mtmr5 | F: 5’-GCTTTTTGCGCCAAAGACTCT-3’ |
|  | R: 5’-CAGGTTCCCGATCACATTCTC-3’ |
| Braf | F:5’-TGATGCGCTGTCTTCGGAAAT-3’ |
|  | R: 5’-GCCAGGCTCAAAATCAAACACT-3’ |
| Map3k1 | F: 5’-CGCCCTGCCCATCTACTTC-3’ |
|  | R: 5’-CTCCATCTCTCGACCGGAGG-3’ |
| Map3k3 | F:5’-ATAAGGACACAGGTCACCCAA-3’ |
|  | R: 5’-TGCTCCACATCTTCGTATCTCA-3’ |
| Map3k4 | F: 5’-AGGCAGGAGTGCATGTTGG-3’ |
|  | R: 5’-AAGTCCTCTGGATCGGATTCC-3’ |
| Map3k7 | F: 5’-CGGATGAGCCGTTACAGTATC-3’ |
|  | R: 5’-ACTCCAAGCGTTTAATAGTGTCG-3’ |
| Map3k20 | F: 5’-CGGACTGCCAATGGTTAGACACTC-3’ |
|  | R: 5’-CTCACCGCAGCAGCATAGGAATC-3’ |
| Map4k1 | F: 5’-CCCATTCTTATGTGGGGCATT-3’ |
|  | R: 5’-TGGAAGAGCACCGACTTTGC-3’ |
| Prkcd | F: 5’-CCTCCTGTACGAAATGCTCATC-3’ |
|  | R: 5’-GTTTCCTGTTACTCCCAGCCT-3’ |
| Csnk1d | F:5’-ACGCCGGGATCGAGAAGAA-3’ |
|  | R: 5’-CCGACCGGGAATCTGTGAG-3’ |
| Pik3c2a | F:5’-TGACAGCCCAAGAGGCTTTG-3’ |
|  | R:5’-CCTGGGTGAGCTTTTCTACATC-3’ |
| Ulk1 | F: 5’-AAGTTCGAGTTCTCTCGCAAG-3’ |
|  | R: 5’-CGATGTTTTCGTGCTTTAGTTCC-3’ |
| Ptpn22 | F:5’-CAGCAACTACTGAAAGAAGCCC-3’ |
|  | R: 5’-AGGATAGATTTTGTCGGCCTTG-3’ |
| Prkar1a | F: 5’-ATGGCGTCTGGCAGTATGG-3’ |
|  | R: 5’-GCTGCACGATGGAGTCCTTC-3’ |
| Prkag2 | F: 5’-AAAGAACCCTAGCCTGAAGAGG-3’ |
|  | R: 5’-ACCTTCCGAGATGAATGCTTTT-3’ |
| Dyrk1a | F: 5’-TGCACCGTCGTTCTCATTCC-3’  R: 5’-CTGGTCACTTATGCTCGGCT-3’ |
| Inpp5d | F: 5’-GCCCCTGCATGGGAAATCAA-3’ |
|  | R: 5’-TGGGTAGCTGGTCATAACTCC-3’ |
| Cdk1 | F: 5’-CCTGCTTATCAATGCAGAGGG-3’ |
|  | R: 5’-TGCGGGTCACCATTTCAGC-3’ |
| Cdk5 | F: 5’-CCCTGAGATTGTGAAGTCATTCC-3’ |
|  | R: 5’-CCAATTTCAACTCCCCATTCCT-3’ |
| Cdk11B | F: 5’-GACCGCGATTCCAAAAGGGAT-3’ |
|  | R: 5’-CCGAGACATTTGCTGGGGT-3’ |
| Cdk13 | F:5’-AAATATGTGGGCCTCGCTATG-3’ |
|  | R: 5’-CCCTGGCTTTGTAAACTTGTCC-3’ |
| Btk | F: 5’-AAGAAGCGCCTGTTTCTCTTG-3’ |
|  | R: 5’-GGTACGGGAACCTTTCAATGAT-3’ |
| Phka2 | F: 5’-TCCTGTGTTACCAGAACCCAG-3’ |
|  | R: 5’-CCCCAAACAGCCAATATGCT-3’ |
| Gprk6 | F:5’-GAGAACATCGTAGCGAACACG-3’ |
|  | R: 5’-ACAGAACTCACGAAATAACAGGC-3’ |
| Stk11 | F: 5’-TTGGGCCTTTTCTCCGAGG-3’ |
|  | R: 5’-CAGGTCCCCCATCAGGTACT-3’ |
| Mark2 | F:5’-TTTCGCCAGATAGTGTCTGCT-3’ |
|  | R: 5’-CCACAGAAAGTATCCAGCTTGTT-3’ |
| Raf1 | F: 5’-TGGACTCAAAGATGCGGTGTT-3’ |
|  | R: 5’-AAAACCCGGATAGTATTGCTTGT-3’ |
| Pip5k1a | F:5’-CAGCATCTGGAATCAAGAGAGC-3’ |
|  | R: 5’-ATCAACACTTCGATGGCCTATTT-3’ |
| Pip5k3 | F: 5’-TCCCCGACACTGGACTCTG-3’ |
|  | R: 5’-GGCTGGCCCAACTTGAACT-3’ |
| Itpkb | F: 5’-CGGCGCAGGCTGAATAGTAG-3’ |
|  | R: 5’-ATGCCCACTTTCTGGTTCACC-3’ |
| Melk | F: 5’-TATGAAACGATTGGGACAGGTG-3’ |
|  | R: 5’-CACCACGGCTAATGGGCTT-3’ |
| Epha10 | F: 5’-CGCCGAAGAAGTTATCCTCCT-3’ |
|  | R: 5’-GGGACGGTCGTGTTCATCC-3’ |
| Tlr4 | F: 5’-ATGGCATGGCTTACACCACC-3’ |
|  | R: 5’-GAGGCCAATTTTGTCTCCACA-3’ |
| Md2 | F: 5’-CGCTGCTTTCTCCCATATTGA-3’ |
|  | R: 5’-CCTCAGTCTTATGCAGGGTTCA-3’ |
| Cd14 | F: 5’-CTCTGTCCTTAAAGCGGCTTAC-3’ |
|  | R: 5’-GTTGCGGAGGTTCAAGATGTT-3’ |
| Il-1β | [F:5'-GCA ACT GTT CCT GAA CTC AAC T](http://www.vazyme.com/product/167.html)-3’ |
|  | R: 5’-ATCTTTTGGGGTCCGTCAACT-3’ |
| Il-6 | F: 5’-TAG TCC TTC CTA CCC CAA TTT CC-3’ |
|  | R: 5’-TTG GTC CTT AGC CAC TCC TTC-3’ |
| CXCL15 | F: 5’-CAA GGC TGG TCC ATG CTC C-3’ |
|  | R: 5’-TGC TAT CAC TTC CTT TCT GTT GC-3’ |
| Tnf-ɑ | F: 5’-CCC TCA CAC TCA GAT CAT CTT CT-3’ |
|  | R: 5’-GCT ACG ACG TGG GCT ACA G-3’ |
| β-actin | F:5’-GGC TGT ATT CCC CTC CAT CG-3’ |
|  | R: 5’-CCA GTT GGT AAC AAT GCC ATG T-3’ |
