# Supplementary material for: LKB1‐MARK2 signalling mediates lipopolysaccharide‐induced production of cytokines in mouse macrophages
Source: J Cell Mol Med. 2020 Aug 25;24(19):11307–17. doi: 10.1111/jcmm.15710 (PMC7576310; doi:10.1111/jcmm.15710)
Supplement: Supplementary file 4 — Table S2 [file JCMM-24-11307-s004.doc]

| **Supplementary Table 2. A total of 765 phosphorylation sites originated from 448 proteins** | | | | | | | |
| --- | --- | --- | --- | --- | --- | --- | --- |
| **IPI** | **Protein** | **Site** | **SiteIndex** | **Seq** | **Protein names** | **Gene names** | **Length** |
| IPI00551236.2 | P54227 | R.ASGQAFELILS*PR.S | 25 | AFELILSPRSKES | Stathmin (Leukemia-associated gene protein) (Leukemia-associated phosphoprotein p18) (Metablastin) (Oncoprotein 18) (Op18) (Phosphoprotein p19) (pp19) (Prosolin) (Protein Pr22) (pp17) | Stmn1 Lag Lap18 Pr22 | 149 |
| IPI00551236.2 | P54227 | K.ESVPDFPLS*PPK.K | 38 | VPDFPLSPPKKKD | Stathmin (Leukemia-associated gene protein) (Leukemia-associated phosphoprotein p18) (Metablastin) (Oncoprotein 18) (Op18) (Phosphoprotein p19) (pp19) (Prosolin) (Protein Pr22) (pp17) | Stmn1 Lag Lap18 Pr22 | 149 |
| IPI00655152.1 | Q8BZR9 | K.AEAPAGPALGLPS*PEVESGLER.G | 25 | PALGLPSPEVESG | Nuclear cap-binding protein subunit 3 | Ncbp3 | 615 |
| IPI00474783.3 | Q5SWU9 | R.FIIGSVSEDNS*EDEISNLVK.L | 29 | SVSEDNSEDEISN | Acetyl-CoA carboxylase 1 (ACC1) (EC 6.4.1.2) (ACC-alpha) (Acetyl-CoA carboxylase 265) [Includes: Biotin carboxylase (EC 6.3.4.14)] | Acaca Acac Gm738 | 2345 |
| IPI00314743.4 | A0A0R4J0P5 | R.EVTPLIGS*PSVQPSCGVIK.R | 298 | VTPLIGSPSVQPS | Proline-serine-threonine phosphatase-interacting protein 1 | Pstpip1 | 415 |
| IPI00314743.4 | A0A0R4J0P5 | K.TTPSAPAASTETLT*PTPER.N | 334 | ASTETLTPTPERN | Proline-serine-threonine phosphatase-interacting protein 1 | Pstpip1 | 415 |
| IPI00458476.2 | | R.T*NPITGTWMLHDAIR.H | |  |  |  |  |
| IPI00395068.3 | P19426 | R.SLS*EQPVVDTATATEQAK.Q | 51 | GVKRSLSEQPVVD | Negative elongation factor E (NELF-E) (RNA-binding protein RD) | Nelfe D17h6s45 Rd Rdbp | 375 |
| IPI00395068.3 | P19426 | K.SVWGSLAVQNS*PK.G | 357 | SLAVQNSPKGCHR | Negative elongation factor E (NELF-E) (RNA-binding protein RD) | Nelfe D17h6s45 Rd Rdbp | 375 |
| IPI00341869.5 | Q80XU3 | K.VVDYSQFQES*DDADEDYGR.D | 19 | YSQFQESDDADED | Nuclear ubiquitous casein and cyclin-dependent kinase substrate 1 (JC7) | Nucks1 Nucks | 234 |
| IPI00222531.6 | Q7TNF9 | R.S*LEGLNQELEEVFVK.E | 213 | SPCLHRSLEGLNQ | Protein FAM117A | Fam117a | 451 |
| IPI00222531.6 | Q7TNF9 | R.VFEEATS*PGPDLAFLTSCPDK.N | 354 | VFEEATSPGPDLA | Protein FAM117A | Fam117a | 451 |
| IPI00653346.1 | | R.ELAGSVESLPSSPWNS*PR.V | |  |  |  |  |
| IPI00475159.1 | Q9CR86 | R.GNVVPS*PLPTR.R | 42 | RGNVVPSPLPTRR | Calcium-regulated heat stable protein 1 (Calcium-regulated heat-stable protein of 24 kDa) (CRHSP-24) | Carhsp1 | 148 |
| IPI00785260.1 | Q3U1J1 | R.TTATLDPT*SPAPGEGPSGR.K | 179 | TATLDPTSPAPGE | TCF3 fusion partner homolog (Protein FB1) (Protein amida) | Tfpt Amida | 259 |
| IPI00116202.1 | Q99L02 | R.DLFSLDSEGPSPTS*PPLR.S | 236 | EGPSPTSPPLRSS | PAXIP1-associated glutamate-rich protein 1A (PAXIP1-associated protein 1) (PTIP-associated protein 1) | Pagr1a Pa1 Pagr | 253 |
| IPI00620611.3 | B1AXD8 | R.TLDFDPLLSPAS*PK.R | 21 | PLLSPASPKRRRC | Akirin-2 | Akirin2 | 201 |
| IPI00117305.2 | P70181 | K.ATS*QEIVSSISQEWK.D | 421 | AALKATSQEIVSS | Phosphatidylinositol 4-phosphate 5-kinase type-1 beta (PIP5K1-beta) (PtdIns(4)P-5-kinase 1 beta) (EC 2.7.1.68) (Phosphatidylinositol 4-phosphate 5-kinase type I alpha) (PIP5KIalpha) (Phosphatidylinositol 4-phosphate 5-kinase type I beta) (PIP5KIbeta) | Pip5k1b Pip5k1a | 539 |
| IPI00117305.2 | P70181 | R.DLLTEGQS*FSSLDEEALGSR.H | 445 | LLTEGQSFSSLDE | Phosphatidylinositol 4-phosphate 5-kinase type-1 beta (PIP5K1-beta) (PtdIns(4)P-5-kinase 1 beta) (EC 2.7.1.68) (Phosphatidylinositol 4-phosphate 5-kinase type I alpha) (PIP5KIalpha) (Phosphatidylinositol 4-phosphate 5-kinase type I beta) (PIP5KIbeta) | Pip5k1b Pip5k1a | 539 |
| IPI00757909.1 | Q9D0L7 | R.S*AEDLTDGSYDDILNAEQLK.K | 43 | RLRPSRSAEDLTD | Armadillo repeat-containing protein 10 | Armc10 | 306 |
| IPI00229362.1 | P70255 | R.NWTEDIEGGISS*PVK.K | 323 | IEGGISSPVKKTE | Nuclear factor 1 C-type (NF1-C) (Nuclear factor 1/C) (CCAAT-box-binding transcription factor) (CTF) (Nuclear factor I/C) (NF-I/C) (NFI-C) (TGGCA-binding protein) | Nfic | 439 |
| IPI00229362.1 | P70255 | K.SPFNSPS*PQDSPR.L | 339 | SPFNSPSPQDSPR | Nuclear factor 1 C-type (NF1-C) (Nuclear factor 1/C) (CCAAT-box-binding transcription factor) (CTF) (Nuclear factor I/C) (NF-I/C) (NFI-C) (TGGCA-binding protein) | Nfic | 439 |
| IPI00129918.1 | P97801 | R.GTGQS*DDSDIWDDTALIK.A | 25 | RRGTGQSDDSDIW | Survival motor neuron protein | Smn1 Smn | 288 |
| IPI00110093.1 | | R.YGVYEDENYEVES*DDEEIPFK.C | |  |  |  |  |
| IPI00223531.3 | O08584 | R.ESSQLWGCGPGDLPS*PGK.V | 171 | GPGDLPSPGKVRS | Krueppel-like factor 6 (Core promoter element-binding protein) | Klf6 Copeb Cpbp | 283 |
| IPI00719993.1 | | K.DGELPVEDDIDLS*DVELDDLEKDEL.- | | |  |  |  |
| IPI00230005.3 | P68181 | R.TWT*LCGTPEYLAPEIILSK.G | 198 | VKGRTWTLCGTPE | cAMP-dependent protein kinase catalytic subunit beta (PKA C-beta) (EC 2.7.11.11) | Prkacb Pkacb | 351 |
| IPI00480432.2 | Q8R361 | K.YDLESASAILPSSALEDPELGS*LGK.M | 224 | EDPELGSLGKMGK | Rab11 family-interacting protein 5 (Rab11-FIP5) (Rab11-interacting protein Rip11) | Rab11fip5 D6Ertd32e Rip11 | 645 |
| IPI00480432.2 | Q8R361 | R.TYS*DEASQLR.A | 307 | THKRTYSDEASQL | Rab11 family-interacting protein 5 (Rab11-FIP5) (Rab11-interacting protein Rip11) | Rab11fip5 D6Ertd32e Rip11 | 645 |
| IPI00621765.4 | Q8BHX3 | K.EQVYNISINGS*PLADSK.E | 228 | NISINGSPLADSK | Borealin (Cell division cycle-associated protein 8) (MESrg) | Cdca8 | 289 |
| IPI00653368.1 | Q8BP27 | R.ENPPS*PPT*SPAAPQPR.E | 67 | TRENPPSPPTSPA | Swi5-dependent recombination DNA repair protein 1 homolog (Meiosis protein 5 homolog) | Sfr1 Mei5 Meir5 | 319 |
| IPI00653368.1 | Q8BP27 | R.ENPPS*PPTS*PAAPQPR.E | 67 | TRENPPSPPTSPA | Swi5-dependent recombination DNA repair protein 1 homolog (Meiosis protein 5 homolog) | Sfr1 Mei5 Meir5 | 319 |
| IPI00111523.3 | Q6NVF4 | K.LSGSCAPSTGFASQPS*SPR.V | 945 | GFASQPSSPRVGG | DNA helicase B (EC 3.6.4.12) | Helb | 1074 |
| IPI00111523.3 | Q6NVF4 | R.WLSASVNDDVDT*DEESAQLR.G | 992 | VNDDVDTDEESAQ | DNA helicase B (EC 3.6.4.12) | Helb | 1074 |
| IPI00111523.3 | Q6NVF4 | R.IGDGFPFDEES*PSK.F | 1015 | FPFDEESPSKFRM | DNA helicase B (EC 3.6.4.12) | Helb | 1074 |
| IPI00415385.1 | Q8K019 | K.SATSGDIWPGLSAYDNS*PR.S | 221 | LSAYDNSPRSPHS | Bcl-2-associated transcription factor 1 (Btf) | Bclaf1 Btf Kiaa0164 | 919 |
| IPI00415385.1 | Q8K019 | K.SATSGDIWPGLSAY*DNSPR.S | 218 | WPGLSAYDNSPRS | Bcl-2-associated transcription factor 1 (Btf) | Bclaf1 Btf Kiaa0164 | 919 |
| IPI00415385.1 | Q8K019 | R.ADGDWDDQEVLDYFS*DK.E | 383 | EVLDYFSDKESAK | Bcl-2-associated transcription factor 1 (Btf) | Bclaf1 Btf Kiaa0164 | 919 |
| IPI00415385.1 | Q8K019 | R.IDIS*PSALR.K | 656 | HRRIDISPSALRK | Bcl-2-associated transcription factor 1 (Btf) | Bclaf1 Btf Kiaa0164 | 919 |
| IPI00124064.3 | Q8VDZ4 | R.GLGS*PEPGTTAPYLGR.S | 621 | RSRGLGSPEPGTT | Palmitoyltransferase ZDHHC5 (EC 2.3.1.225) (Zinc finger DHHC domain-containing protein 5) (DHHC-5) | Zdhhc5 Kiaa1748 | 715 |
| IPI00124064.3 | Q8VDZ4 | K.SIGSASPGPGQPPLSSPT*R.G | 696 | PPLSSPTRGGVKK | Palmitoyltransferase ZDHHC5 (EC 2.3.1.225) (Zinc finger DHHC domain-containing protein 5) (DHHC-5) | Zdhhc5 Kiaa1748 | 715 |
| IPI00135443.2 | Q64511 | K.SQDFGNLFSFPS*YSQK.S | 1439 | NLFSFPSYSQKSE | DNA topoisomerase 2-beta (EC 5.99.1.3) (DNA topoisomerase II, beta isozyme) | Top2b | 1612 |
| IPI00135443.2 | Q64511 | K.FDS*NEEDTASVFAPSFGLK.Q | 1453 | DSAKFDSNEEDTA | DNA topoisomerase 2-beta (EC 5.99.1.3) (DNA topoisomerase II, beta isozyme) | Top2b | 1612 |
| IPI00135443.2 | Q64511 | K.IVETINSDS*DSEFGIPK.K | 1511 | ETINSDSDSEFGI | DNA topoisomerase 2-beta (EC 5.99.1.3) (DNA topoisomerase II, beta isozyme) | Top2b | 1612 |
| IPI00135443.2 | Q64511 | K.IVETINS*DS*DSEFGIPK.K | 1509 | IVETINSDSDSEF | DNA topoisomerase 2-beta (EC 5.99.1.3) (DNA topoisomerase II, beta isozyme) | Top2b | 1612 |
| IPI00135443.2 | Q64511 | K.TSFDQDS*DVDIFPSDFTSEPPALPR.T | 1568 | TSFDQDSDVDIFP | DNA topoisomerase 2-beta (EC 5.99.1.3) (DNA topoisomerase II, beta isozyme) | Top2b | 1612 |
| IPI00667587.1 | D3YZP9 | K.SGGIVIS*PFR.L | 45 | SGGIVISPFRLEE | Coiled-coil domain-containing protein 6 | Ccdc6 | 469 |
| IPI00667587.1 | D3YZP9 | K.LDQPVSAPPS*PR.D | 237 | PVSAPPSPRDISM | Coiled-coil domain-containing protein 6 | Ccdc6 | 469 |
| IPI00133685.1 | Q9D1F4 | K.S*LPVSVPVWAFK.E | 184 | TQQYAKSLPVSVP | Proline-rich AKT1 substrate 1 (Proline-rich AKT substrate) | Akt1s1 Pras | 257 |
| IPI00469331.2 | Q8R4R6 | R.GVLSS*PSLAFT*TPIR.T | 258 | DRGVLSSPSLAFT | Nucleoporin NUP35 (35 kDa nucleoporin) (Mitotic phosphoprotein 44) (MP-44) (Nuclear pore complex protein Nup53) (Nucleoporin NUP53) | Nup35 Mp44 Nup53 | 325 |
| IPI00274639.4 | Q9ESL4 | R.SDS*SADCQWLDTLR.M | 567 | PGSRSDSSADCQW | Mitogen-activated protein kinase kinase kinase 20 (EC 2.7.11.25) (Human cervical cancer suppressor gene 4 protein) (HCCS-4) (Leucine zipper- and sterile alpha motif kinase ZAK) (Leucine zipper- and sterile alpha motif-containing kinase) (MLK-like mitogen-activated protein triple kinase) (Mitogen-activated protein kinase kinase kinase MLT) (Mixed lineage kinase-related kinase) (MLK-related kinase) (MRK) (Sterile alpha motif- and leucine zipper-containing kinase AZK) | Map3k20 Mltk Zak | 802 |
| IPI00274639.4 | Q9ESL4 | K.YQQITPSINPS*R.S | 634 | TPSINPSRSSSPT | Mitogen-activated protein kinase kinase kinase 20 (EC 2.7.11.25) (Human cervical cancer suppressor gene 4 protein) (HCCS-4) (Leucine zipper- and sterile alpha motif kinase ZAK) (Leucine zipper- and sterile alpha motif-containing kinase) (MLK-like mitogen-activated protein triple kinase) (Mitogen-activated protein kinase kinase kinase MLT) (Mixed lineage kinase-related kinase) (MLK-related kinase) (MRK) (Sterile alpha motif- and leucine zipper-containing kinase AZK) | Map3k20 Mltk Zak | 802 |
| IPI00274639.4 | Q9ESL4 | R.NFS*SLNLSSR.D | 649 | GLSRNFSSLNLSS | Mitogen-activated protein kinase kinase kinase 20 (EC 2.7.11.25) (Human cervical cancer suppressor gene 4 protein) (HCCS-4) (Leucine zipper- and sterile alpha motif kinase ZAK) (Leucine zipper- and sterile alpha motif-containing kinase) (MLK-like mitogen-activated protein triple kinase) (Mitogen-activated protein kinase kinase kinase MLT) (Mixed lineage kinase-related kinase) (MLK-related kinase) (MRK) (Sterile alpha motif- and leucine zipper-containing kinase AZK) | Map3k20 Mltk Zak | 802 |
| IPI00274639.4 | Q9ESL4 | R.NFSS*LNLSSR.D | 650 | LSRNFSSLNLSSR | Mitogen-activated protein kinase kinase kinase 20 (EC 2.7.11.25) (Human cervical cancer suppressor gene 4 protein) (HCCS-4) (Leucine zipper- and sterile alpha motif kinase ZAK) (Leucine zipper- and sterile alpha motif-containing kinase) (MLK-like mitogen-activated protein triple kinase) (Mitogen-activated protein kinase kinase kinase MLT) (Mixed lineage kinase-related kinase) (MLK-related kinase) (MRK) (Sterile alpha motif- and leucine zipper-containing kinase AZK) | Map3k20 Mltk Zak | 802 |
| IPI00649373.1 | Q68FF6 | K.SLSS*PTDNLELSAR.S | 371 | QGKSLSSPTDNLE | ARF GTPase-activating protein GIT1 (ARF GAP GIT1) (G protein-coupled receptor kinase-interactor 1) (GRK-interacting protein 1) | Git1 | 770 |
| IPI00649373.1 | Q68FF6 | R.S*MDSSDLSDGAVTLQEYLELK.K | 419 | RNNRARSMDSSDL | ARF GTPase-activating protein GIT1 (ARF GAP GIT1) (G protein-coupled receptor kinase-interactor 1) (GRK-interacting protein 1) | Git1 | 770 |
| IPI00664271.1 | Q9CZ91 | K.DARQS*APAAESSESTSGEGR.C | 139 | FKDARQSAPAAES | Serum response factor-binding protein 1 (SRF-dependent transcription regulation-associated protein) (p49/STRAP) | Srfbp1 | 441 |
| IPI00554927.1 | | R.ISHELESSS*SEVN.- |  |  |  |  |  |
| IPI00121172.2 | Q5HZH2 | R.S*LDAFAEEVGAALR.A | 24 | RRPSGRSLDAFAE | Ribosome biogenesis protein TSR3 homolog | Tsr3 | 323 |
| IPI00170092.2 | Q8K3I9 | R.TSS*LDTITGPYLTGQWPR.D | 163 | TIRRTSSLDTITG | Glucocorticoid-induced transcript 1 protein (Glucocorticoid-induced gene 18 protein) (Testhymin) (Thymocyte/spermatocyte selection protein 1) | Glcci1 Gig18 Tssn1 | 537 |
| IPI00134747.3 | Q9D2E2 | R.S*LQSQPGTQTLAEAEDGPPTK.Q | 349 | KDKRKRSLQSQPG | Target of EGR1 protein 1 | Toe1 | 511 |
| IPI00226149.5 | Q8BVY0 | K.ALLATET*PEASAPGTS*GKK.Q | 399 | ALLATETPEASAP | Ribosomal L1 domain-containing protein 1 | Rsl1d1 | 452 |
| IPI00330763.3 | Q80XP8 | K.VSSLS*PEQEQGLWK.Q | 193 | HKVSSLSPEQEQG | Protein FAM76B | Fam76b | 339 |
| IPI00352179.2 | Q64701 | R.IFLGADAEEEIGT*PR.K | 332 | AEEEIGTPRKFTA | Retinoblastoma-like protein 1 (107 kDa retinoblastoma-associated protein) (p107) (pRb1) | Rbl1 | 1063 |
| IPI00352179.2 | Q64701 | R.SFAPST*PLTGR.R | 369 | RSFAPSTPLTGRR | Retinoblastoma-like protein 1 (107 kDa retinoblastoma-associated protein) (p107) (pRb1) | Rbl1 | 1063 |
| IPI00352179.2 | Q64701 | K.EAVTT*PVASATQSVSR.L | 385 | EKEAVTTPVASAT | Retinoblastoma-like protein 1 (107 kDa retinoblastoma-associated protein) (p107) (pRb1) | Rbl1 | 1063 |
| IPI00625247.3 | Q922B6 | R.STFS*LPEEEEEPEPLVFAEQPSVK.L | 32 | SLRSTFSLPEEEE | E3 ubiquitin-protein ligase TRAF7 (EC 2.3.2.27) (RING-type E3 ubiquitin transferase TRAF7) (TNF receptor-associated factor 7) | Traf7 | 594 |
| IPI00113783.1 | O09039 | R.GSTDS*LDQGASPGVLLDPACQK.T | 309 | PRGSTDSLDQGAS | SH2B adapter protein 3 (Lymphocyte adapter protein) (Lymphocyte-specific adapter protein Lnk) (Signal transduction protein Lnk) | Sh2b3 Lnk | 548 |
| IPI00554855.1 | Q05512 | K.LDT*FCGSPPYAAPELFQGK.K | 208 | FGNKLDTFCGSPP | Serine/threonine-protein kinase MARK2 (EC 2.7.11.1) (EC 2.7.11.26) (ELKL motif kinase 1) (EMK-1) (MAP/microtubule affinity-regulating kinase 2) (PAR1 homolog) (PAR1 homolog b) (Par-1b) (mPar-1b) | Mark2 Emk | 776 |
| IPI00554855.1 | Q05512 | K.VPAS*PLPGLDR.K | 453 | TAKVPASPLPGLD | Serine/threonine-protein kinase MARK2 (EC 2.7.11.1) (EC 2.7.11.26) (ELKL motif kinase 1) (EMK-1) (MAP/microtubule affinity-regulating kinase 2) (PAR1 homolog) (PAR1 homolog b) (Par-1b) (mPar-1b) | Mark2 Emk | 776 |
| IPI00480574.1 | Q99L90 | K.GDQVLNFS*DAEDLIDDSK.L | 282 | DQVLNFSDAEDLI | Microspherule protein 1 (58 kDa microspherule protein) | Mcrs1 Msp58 | 462 |
| IPI00229455.1 | Q60591 | R.DAGLS*PEQPALALAGVAASPR.F | 136 | GRDAGLSPEQPAL | Nuclear factor of activated T-cells, cytoplasmic 2 (NF-ATc2) (NFATc2) (NFAT pre-existing subunit) (NF-ATp) (T-cell transcription factor NFAT1) | Nfatc2 Nfat1 Nfatp | 927 |
| IPI00229455.1 | Q60591 | R.LS*PGAYPTVIQQQTAPSQR.A | 860 | NQGQRLSPGAYPT | Nuclear factor of activated T-cells, cytoplasmic 2 (NF-ATc2) (NFATc2) (NFAT pre-existing subunit) (NF-ATp) (T-cell transcription factor NFAT1) | Nfatc2 Nfat1 Nfatp | 927 |
| IPI00420527.2 | | R.APPAAPAADEPGS*PGGPPR.R | |  |  |  |  |
| IPI00420527.2 | | R.VS*LQALEAETEAGTDAEAVIQR.H | | |  |  |  |
| IPI00265987.3 | Q9WU62 | R.ASWGLQDS*PGSTDSPWQER.V | 245 | SWGLQDSPGSTDS | Inner centromere protein | Incenp | 880 |
| IPI00265987.3 | Q9WU62 | R.S*LISQDSQVPLASK.Y | 284 | IRSVRRSLISQDS | Inner centromere protein | Incenp | 880 |
| IPI00458918.2 | Q5SZT7 | R.IGELGAPEVWGLS*PK.I | 141 | PEVWGLSPKIPEP | NKAP-like protein | Nkapl | 395 |
| IPI00309262.2 | P49615 | K.IGEGTYGT*VFK.A | 17 | GEGTYGTVFKAKN | Cyclin-dependent-like kinase 5 (EC 2.7.11.1) (CR6 protein kinase) (CRK6) (Cell division protein kinase 5) (Serine/threonine-protein kinase PSSALRE) (Tau protein kinase II catalytic subunit) (TPKII catalytic subunit) | Cdk5 Cdkn5 Crk6 | 292 |
| IPI00626480.3 | A2AIV8 | R.NSQELSLPQDLEEDAQLS*DK.G | 451 | EEDAQLSDKGVLA | Caspase recruitment domain-containing protein 9 | Card9 | 536 |
| IPI00751399.1 | Q6P5G6 | R.SESLIDASEDS*QLEAAIR.A | 266 | IDASEDSQLEAAI | UBX domain-containing protein 7 | Ubxn7 Kiaa0794 Ubxd7 | 467 |
| IPI00775806.1 | Q9Z2D6 | K.VGDTS*LDPNDFDFTVTGR.G | 149 | EKVGDTSLDPNDF | Methyl-CpG-binding protein 2 (MeCp-2 protein) (MeCp2) | Mecp2 | 484 |
| IPI00654422.1 | P97855 | K.STS*PAPADVAPAQEDLR.T | 231 | DVQKSTSPAPADV | Ras GTPase-activating protein-binding protein 1 (G3BP-1) (EC 3.6.4.12) (EC 3.6.4.13) (ATP-dependent DNA helicase VIII) (GAP SH3 domain-binding protein 1) (HDH-VIII) | G3bp1 G3bp | 465 |
| IPI00124240.1 | P11440 | K.IGEGTY*GVVYK.G | 15 | KIGEGTYGVVYKG | Cyclin-dependent kinase 1 (CDK1) (EC 2.7.11.22) (EC 2.7.11.23) (Cell division control protein 2 homolog) (Cell division protein kinase 1) (p34 protein kinase) | Cdk1 Cdc2 Cdc2a Cdkn1 | 297 |
| IPI00458240.1 | Q91VL8 | K.YLLGNAPVS*PSSQK.L | 200 | LGNAPVSPSSQKL | Telomeric repeat-binding factor 2-interacting protein 1 (TERF2-interacting telomeric protein 1) (TRF2-interacting telomeric protein 1) (Repressor/activator protein 1 homolog) (RAP1 homolog) | Terf2ip Rap1 MNCb-0448 MNCb-0628 | 393 |
| IPI00125960.1 | Q62433 | R.T*ASGSSVTSLEGTR.S | 328 | RLMRSRTASGSSV | Protein NDRG1 (N-myc downstream-regulated gene 1 protein) (Protein Ndr1) | Ndrg1 Ndr1 Ndrl Tdd5 | 394 |
| IPI00659338.1 | | R.VSS*GVYEPVVIESH.- |  |  |  |  |  |
| IPI00625592.1 | P08775 | K.YTPTSPSYSPS*SPEYTPASPK.Y | 1849 | SPSYSPSSPEYTP | DNA-directed RNA polymerase II subunit RPB1 (RNA polymerase II subunit B1) (EC 2.7.7.6) (DNA-directed RNA polymerase II subunit A) (DNA-directed RNA polymerase III largest subunit) | Polr2a Rpii215 Rpo2-1 | 1970 |
| IPI00625592.1 | P08775 | K.YTPTSPS*YS*PSSPEYTPASPK.Y | 1845 | YTPTSPSYSPSSP | DNA-directed RNA polymerase II subunit RPB1 (RNA polymerase II subunit B1) (EC 2.7.7.6) (DNA-directed RNA polymerase II subunit A) (DNA-directed RNA polymerase III largest subunit) | Polr2a Rpii215 Rpo2-1 | 1970 |
| IPI00625592.1 | P08775 | K.YTPTSPSY*SPSSPEYTPAS*PK.Y | 1846 | TPTSPSYSPSSPE | DNA-directed RNA polymerase II subunit RPB1 (RNA polymerase II subunit B1) (EC 2.7.7.6) (DNA-directed RNA polymerase II subunit A) (DNA-directed RNA polymerase III largest subunit) | Polr2a Rpii215 Rpo2-1 | 1970 |
| IPI00625592.1 | P08775 | K.YSPTS*PTYSPTTPK.Y | 1878 | PKYSPTSPTYSPT | DNA-directed RNA polymerase II subunit RPB1 (RNA polymerase II subunit B1) (EC 2.7.7.6) (DNA-directed RNA polymerase II subunit A) (DNA-directed RNA polymerase III largest subunit) | Polr2a Rpii215 Rpo2-1 | 1970 |
| IPI00625592.1 | P08775 | K.YSPTSPTY*SPTTPK.Y | 1881 | SPTSPTYSPTTPK | DNA-directed RNA polymerase II subunit RPB1 (RNA polymerase II subunit B1) (EC 2.7.7.6) (DNA-directed RNA polymerase II subunit A) (DNA-directed RNA polymerase III largest subunit) | Polr2a Rpii215 Rpo2-1 | 1970 |
| IPI00625592.1 | P08775 | K.YSPTSPTYSPT*SPVYTPTSPK.Y | 1898 | SPTYSPTSPVYTP | DNA-directed RNA polymerase II subunit RPB1 (RNA polymerase II subunit B1) (EC 2.7.7.6) (DNA-directed RNA polymerase II subunit A) (DNA-directed RNA polymerase III largest subunit) | Polr2a Rpii215 Rpo2-1 | 1970 |
| IPI00625592.1 | P08775 | K.YSPTSPT*YSPTSPVYTPT*SPK.Y | 1894 | YSPTSPTYSPTSP | DNA-directed RNA polymerase II subunit RPB1 (RNA polymerase II subunit B1) (EC 2.7.7.6) (DNA-directed RNA polymerase II subunit A) (DNA-directed RNA polymerase III largest subunit) | Polr2a Rpii215 Rpo2-1 | 1970 |
| IPI00625592.1 | P08775 | K.YSPTSPT*YSPTSPVYTPTS*PK.Y | 1894 | YSPTSPTYSPTSP | DNA-directed RNA polymerase II subunit RPB1 (RNA polymerase II subunit B1) (EC 2.7.7.6) (DNA-directed RNA polymerase II subunit A) (DNA-directed RNA polymerase III largest subunit) | Polr2a Rpii215 Rpo2-1 | 1970 |
| IPI00625592.1 | P08775 | K.YSPTSPTYS*PTSPVYTPT*SPK.Y | 1896 | PTSPTYSPTSPVY | DNA-directed RNA polymerase II subunit RPB1 (RNA polymerase II subunit B1) (EC 2.7.7.6) (DNA-directed RNA polymerase II subunit A) (DNA-directed RNA polymerase III largest subunit) | Polr2a Rpii215 Rpo2-1 | 1970 |
| IPI00625592.1 | P08775 | K.YSPTSPTYSPT*SPK.Y | 1919 | SPTYSPTSPKYSP | DNA-directed RNA polymerase II subunit RPB1 (RNA polymerase II subunit B1) (EC 2.7.7.6) (DNA-directed RNA polymerase II subunit A) (DNA-directed RNA polymerase III largest subunit) | Polr2a Rpii215 Rpo2-1 | 1970 |
| IPI00625592.1 | P08775 | K.YSPTS*PTYSPTSPK.Y | 1913 | PKYSPTSPTYSPT | DNA-directed RNA polymerase II subunit RPB1 (RNA polymerase II subunit B1) (EC 2.7.7.6) (DNA-directed RNA polymerase II subunit A) (DNA-directed RNA polymerase III largest subunit) | Polr2a Rpii215 Rpo2-1 | 1970 |
| IPI00128196.1 | Q9Z2D1 | K.SASAISSDSISTSADNFS*PDLR.V | 58 | TSADNFSPDLRVL | Myotubularin-related protein 2 (Phosphatidylinositol-3,5-bisphosphate 3-phosphatase) (EC 3.1.3.95) (Phosphatidylinositol-3-phosphate phosphatase) (EC 3.1.3.64) | Mtmr2 | 643 |
| IPI00752541.1 | Q8BGD9 | K.SPPY*TAFLGNLPYDVTEDSIK.D | 96 | LPKSPPYTAFLGN | Eukaryotic translation initiation factor 4B (eIF-4B) | Eif4b | 611 |
| IPI00752541.1 | Q8BGD9 | K.S*PPYTAFLGNLPYDVTEDSIK.D | 93 | RSRLPKSPPYTAF | Eukaryotic translation initiation factor 4B (eIF-4B) | Eif4b | 611 |
| IPI00648562.1 | Q925J9 | R.LSSS*DSIGPDVTDILSDIAEEASK.L | 772 | MVRLSSSDSIGPD | Mediator of RNA polymerase II transcription subunit 1 (Mediator complex subunit 1) (Peroxisome proliferator-activated receptor-binding protein) (PBP) (PPAR-binding protein) (Thyroid hormone receptor-associated protein complex 220 kDa component) (Trap220) (Thyroid receptor-interacting protein 2) (TR-interacting protein 2) (TRIP-2) | Med1 Crsp210 Drip205 Pbp Pparbp Trap220 Trip2 | 1575 |
| IPI00648562.1 | Q925J9 | R.SQT*PPGVAT*PPIPK.I | 1051 | SSGRSQTPPGVAT | Mediator of RNA polymerase II transcription subunit 1 (Mediator complex subunit 1) (Peroxisome proliferator-activated receptor-binding protein) (PBP) (PPAR-binding protein) (Thyroid hormone receptor-associated protein complex 220 kDa component) (Trap220) (Thyroid receptor-interacting protein 2) (TR-interacting protein 2) (TRIP-2) | Med1 Crsp210 Drip205 Pbp Pparbp Trap220 Trip2 | 1575 |
| IPI00648562.1 | Q925J9 | K.NYGSPLISGS*TPK.H | 1441 | SPLISGSTPKHER | Mediator of RNA polymerase II transcription subunit 1 (Mediator complex subunit 1) (Peroxisome proliferator-activated receptor-binding protein) (PBP) (PPAR-binding protein) (Thyroid hormone receptor-associated protein complex 220 kDa component) (Trap220) (Thyroid receptor-interacting protein 2) (TR-interacting protein 2) (TRIP-2) | Med1 Crsp210 Drip205 Pbp Pparbp Trap220 Trip2 | 1575 |
| IPI00648562.1 | Q925J9 | K.NYGS*PLISGSTPK.H | 1435 | SSKNYGSPLISGS | Mediator of RNA polymerase II transcription subunit 1 (Mediator complex subunit 1) (Peroxisome proliferator-activated receptor-binding protein) (PBP) (PPAR-binding protein) (Thyroid hormone receptor-associated protein complex 220 kDa component) (Trap220) (Thyroid receptor-interacting protein 2) (TR-interacting protein 2) (TRIP-2) | Med1 Crsp210 Drip205 Pbp Pparbp Trap220 Trip2 | 1575 |
| IPI00649424.2 | P24788 | R.DLLSDLQDIS*DSER.K | 270 | SDLQDISDSERKT | Cyclin-dependent kinase 11B (Cell division cycle 2-like protein kinase 1) (Cell division protein kinase 11) (Cyclin-dependent kinase 11) (EC 2.7.11.22) (Galactosyltransferase-associated protein kinase p58/GTA) (PITSLRE serine/threonine-protein kinase CDC2L1) | Cdk11b Cdc2l1 Cdk11 | 784 |
| IPI00649424.2 | P24788 | K.AYT*PVVVTLWYR.A | 584 | SPLKAYTPVVVTL | Cyclin-dependent kinase 11B (Cell division cycle 2-like protein kinase 1) (Cell division protein kinase 11) (Cyclin-dependent kinase 11) (EC 2.7.11.22) (Galactosyltransferase-associated protein kinase p58/GTA) (PITSLRE serine/threonine-protein kinase CDC2L1) | Cdk11b Cdc2l1 Cdk11 | 784 |
| IPI00654295.2 | Q8BXQ8 | R.FSLS*PSLGPQASR.F | 234 | QRRFSLSPSLGPQ | Protein FAM53C | Fam53c | 393 |
| IPI00222035.2 | Q3TBD2 | R.AGS*PNPQSSSGELPR.K | 23 | KKNRAGSPNPQSS | Rho GTPase-activating protein 45 (Minor histocompatibility protein HA-1) | Arhgap45 Hmha1 | 1116 |
| IPI00222035.2 | Q3TBD2 | K.S*WPISISDTEVGLDTSSGDLK.K | 618 | GHQVHKSWPISIS | Rho GTPase-activating protein 45 (Minor histocompatibility protein HA-1) | Arhgap45 Hmha1 | 1116 |
| IPI00222035.2 | Q3TBD2 | K.SWPISISDTEVGLDT*SSGDLK.K | 632 | TEVGLDTSSGDLK | Rho GTPase-activating protein 45 (Minor histocompatibility protein HA-1) | Arhgap45 Hmha1 | 1116 |
| IPI00222035.2 | Q3TBD2 | K.SWPISIS*DTEVGLDTSSGDLK.K | 624 | SWPISISDTEVGL | Rho GTPase-activating protein 45 (Minor histocompatibility protein HA-1) | Arhgap45 Hmha1 | 1116 |
| IPI00222035.2 | Q3TBD2 | K.SWPISISDTEVGLDTS*SGDLK.K | 633 | EVGLDTSSGDLKK | Rho GTPase-activating protein 45 (Minor histocompatibility protein HA-1) | Arhgap45 Hmha1 | 1116 |
| IPI00163030.1 | Q3UPF5 | R.AS*QEFLEDGDPDGLFSR.N | 324 | PSQMRASQEFLED | Zinc finger CCCH-type antiviral protein 1 (ADP-ribosyltransferase diphtheria toxin-like 13) (ARTD13) | Zc3hav1 | 946 |
| IPI00163030.1 | Q3UPF5 | R.TS*AAGFPLVAAQR.N | 350 | SSTSRTSAAGFPL | Zinc finger CCCH-type antiviral protein 1 (ADP-ribosyltransferase diphtheria toxin-like 13) (ARTD13) | Zc3hav1 | 946 |
| IPI00761357.2 | Q5F2E7 | K.DYEIENQNPLASPTNT*LLGSAK.E | 630 | LASPTNTLLGSAK | Nuclear fragile X mental retardation-interacting protein 2 (82 kDa FMRP-interacting protein) (82-FIP) (FMRP-interacting protein 2) | Nufip2 Kiaa1321 | 692 |
| IPI00677977.1 | O35304 | R.YPT*ESEDVKIGVLFAS*K.A | 117 | LRPRYPTESEDVK | Vesicular acetylcholine transporter (VAChT) (Solute carrier family 18 member 3) | Slc18a3 Vacht | 530 |
| IPI00754344.2 | Q8BT07 | K.SPS*AALNDSLVECPK.C | 428 | TSPKSPSAALNDS | Centrosomal protein of 55 kDa (Cep55) | Cep55 | 462 |
| IPI00318314.3 | Q8CFN5 | R.NS*PGLLVSPGNLNK.N | 222 | YGNPRNSPGLLVS | Myocyte-specific enhancer factor 2C (Myocyte enhancer factor 2C) | Mef2c | 474 |
| IPI00310880.4 | Q3TWW8 | R.SHS*PLPAPPSK.A | 303 | SQSRSHSPLPAPP | Serine/arginine-rich splicing factor 6 (Pre-mRNA-splicing factor SRP55) (Splicing factor, arginine/serine-rich 6) | Srsf6 Sfrs6 Srp55 | 339 |
| IPI00110979.1 | E9Q6W4 | R.QLAPGSPSTSAS*SR.G | 275 | SPSTSASSRGVSP | Zinc finger protein 296 (Hepatocellular carcinoma-associated antigen MHCA108) | Znf296 Zfp296 | 445 |
| IPI00227880.1 | P28867 | R.AST*FCGTPDYIAPEILQGLK.Y | 505 | GEGRASTFCGTPD | Protein kinase C delta type (EC 2.7.11.13) (Tyrosine-protein kinase PRKCD) (EC 2.7.10.2) (nPKC-delta) [Cleaved into: Protein kinase C delta type regulatory subunit; Protein kinase C delta type catalytic subunit (Sphingosine-dependent protein kinase-1) (SDK1)] | Prkcd Pkcd | 674 |
| IPI00380308.3 | Q64092 | R.AASDPLLSSVS*PAVSK.A | 553 | PLLSSVSPAVSKA | Transcription factor E3 | Tfe3 Tcfe3 | 572 |
| IPI00120516.2 | Q9ES52 | K.STLS*PDQQLTAWSYDQLPK.D | 935 | HGKSTLSPDQQLT | Phosphatidylinositol 3,4,5-trisphosphate 5-phosphatase 1 (EC 3.1.3.86) (Inositol polyphosphate-5-phosphatase of 145 kDa) (SIP-145) (SH2 domain-containing inositol 5'-phosphatase 1) (SH2 domain-containing inositol phosphatase 1) (SHIP-1) (p150Ship) | Inpp5d 7a33 Ship Ship1 | 1191 |
| IPI00120516.2 | Q9ES52 | R.GEGPPT*PPSQPPLS*PK.K | 964 | RGEGPPTPPSQPP | Phosphatidylinositol 3,4,5-trisphosphate 5-phosphatase 1 (EC 3.1.3.86) (Inositol polyphosphate-5-phosphatase of 145 kDa) (SIP-145) (SH2 domain-containing inositol 5'-phosphatase 1) (SH2 domain-containing inositol phosphatase 1) (SHIP-1) (p150Ship) | Inpp5d 7a33 Ship Ship1 | 1191 |
| IPI00460449.1 | Q6Y5D8 | R.TSPDTTFAEPTCLSAS*PPNAPPR.Q | 591 | PTCLSASPPNAPP | Rho GTPase-activating protein 10 (PH and SH3 domain-containing rhoGAP protein) (PS-GAP) (PSGAP) (Rho-type GTPase-activating protein 10) | Arhgap10 | 786 |
| IPI00659577.1 | Q6ZPR6 | R.SDSS*GGYTLSDVIQSPPSAGLLK.S | 994 | RKRSDSSGGYTLS | Inhibitor of Bruton tyrosine kinase (IBtk) | Ibtk Kiaa1417 | 1352 |
| IPI00461475.4 | Q3U3E2 | R.TSPT*VATQTGASVTSTR.G | 104 | AARTSPTVATQTG | Protein FAM117B (Amyotrophic lateral sclerosis 2 chromosomal region candidate gene 13 protein homolog) | Fam117b Als2cr13 | 584 |
| IPI00652742.1 | Q9R0N8 | K.QKS*VDGDDAKS*EAAK.S | 217 | ELYKQKSVDGDDA | Synaptotagmin-6 (Synaptotagmin VI) (SytVI) | Syt6 | 511 |
| IPI00395177.1 | Q5SSL4 | R.SQGGGDS*VSPTPPEGLAPGVEAGK.G | 70 | SQGGGDSVSPTPP | Active breakpoint cluster region-related protein | Abr | 859 |
| IPI00395177.1 | Q5SSL4 | R.SQGGGDSVS*PTPPEGLAPGVEAGK.G | 72 | GGGDSVSPTPPEG | Active breakpoint cluster region-related protein | Abr | 859 |
| IPI00278134.1 | Q8R1S4 | K.GSDYSWSYQT*PPSS*PSTTMSR.K | 262 | YSWSYQTPPSSPS | Metastasis suppressor protein 1 (Missing in metastasis protein) | Mtss1 Mim | 759 |
| IPI00222813.1 | Q8BLH7 | R.NAWNPSGEGTS*PGETYR.R | 564 | PSGEGTSPGETYR | HIRA-interacting protein 3 | Hirip3 | 601 |
| IPI00758218.1 | Q9D6Z1 | K.EELAS*DLEEMATSSAK.R | 513 | PKEELASDLEEMA | Nucleolar protein 56 (Nucleolar protein 5A) | Nop56 Nol5a | 580 |
| IPI00654366.1 | Q3U1T9 | R.VSS*GGLTESLFILK.E | 633 | PRKRVSSGGLTES | DENN domain-containing protein 1B (Connecdenn 2) | Dennd1b | 766 |
| IPI00648273.1 | Q8R1A4 | R.SLSNS*NPDISGTPTSPDDEVR.S | 900 | SRSLSNSNPDISG | Dedicator of cytokinesis protein 7 (Protein moonlight) | Dock7 Gm430 Kiaa1771 Mnlt | 2130 |
| IPI00648273.1 | Q8R1A4 | R.SPSGSAFGS*QENLR.W | 1428 | SGSAFGSQENLRW | Dedicator of cytokinesis protein 7 (Protein moonlight) | Dock7 Gm430 Kiaa1771 Mnlt | 2130 |
| IPI00762360.1 | Q6P542 | K.GGNVFEALIQDDS*EEEEEEEENR.V | 138 | ALIQDDSEEEEEE | ATP-binding cassette sub-family F member 1 | Abcf1 | 837 |
| IPI00749534.1 | | R.AAPS*GCSPGLALPSCS*HAVSAPR.G | | |  |  |  |
| IPI00749534.1 | | R.AAPS*GCSPGLALPS*CSHAVSAPR.G | | |  |  |  |
| IPI00469000.4 | Q8C145 | K.ESASS*SEVTSAVYNAVSEGTR.F | 191 | IKESASSSEVTSA | Zinc transporter ZIP6 (Endoplasmic reticulum membrane-linked protein) (Ermelin) (Solute carrier family 39 member 6) (Zrt- and Irt-like protein 6) (ZIP-6) | Slc39a6 Zip6 | 765 |
| IPI00758052.2 | Q9WUT3 | R.GFS*FVASSLVQEPSQQDVPK.A | 377 | HLFRGFSFVASSL | Ribosomal protein S6 kinase alpha-2 (S6K-alpha-2) (EC 2.7.11.1) (90 kDa ribosomal protein S6 kinase 2) (p90-RSK 2) (p90RSK2) (MAP kinase-activated protein kinase 1c) (MAPK-activated protein kinase 1c) (MAPKAP kinase 1c) (MAPKAPK-1c) (Protein-tyrosine kinase Mpk-9) (Ribosomal S6 kinase 3) (RSK-3) (pp90RSK3) | Rps6ka2 Mapkapk1c Rsk3 | 733 |
| IPI00648542.1 | B1ASU9 | K.ISDYFEFAGGSGPGT*SPGR.S | 98 | GGSGPGTSPGRSV | Serine/threonine-protein kinase tousled-like 2 | Tlk2 | 750 |
| IPI00648542.1 | B1ASU9 | K.ISDYFEFAGGSGPGTS*PGR.S | 99 | GSGPGTSPGRSVP | Serine/threonine-protein kinase tousled-like 2 | Tlk2 | 750 |
| IPI00755286.1 | Q08639 | K.VFIDQNLS*PGK.G | 23 | FIDQNLSPGKGVV | Transcription factor Dp-1 (DRTF1-polypeptide 1) (E2F dimerization partner 1) | Tfdp1 | 410 |
| IPI00405756.7 | | R.TVS*VPSEGQFPEYPQEGTTK.L | |  |  |  |  |
| IPI00405756.7 | | K.SLTNPT*PIQQQLR.R |  |  |  |  |  |
| IPI00468525.4 | Q61188 | K.ESSIIAPVPTEDVDT*PPR.K | 487 | PTEDVDTPPRKKK | Histone-lysine N-methyltransferase EZH2 (EC 2.1.1.43) (ENX-1) (Enhancer of zeste homolog 2) | Ezh2 Enx1h | 746 |
| IPI00338636.1 | | K.YFS*LDLTHDEVPEFVV.- | |  |  |  |  |
| IPI00229697.2 | Q8CI08 | R.LSGWEEEEESWLYSS*PK.K | 134 | ESWLYSSPKKKLT | SLAIN motif-containing protein 2 | Slain2 Kiaa1458 | 581 |
| IPI00114375.2 | O08553 | R.GLYDGPVCEVSVT*PK.T | 509 | VCEVSVTPKTVTP | Dihydropyrimidinase-related protein 2 (DRP-2) (Unc-33-like phosphoprotein 2) (ULIP-2) | Dpysl2 Crmp2 Ulip2 | 572 |
| IPI00761443.1 | Q8R0S2 | R.NS*WDSPAFSNDVIR.K | 510 | HKETRNSWDSPAF | IQ motif and SEC7 domain-containing protein 1 | Iqsec1 Kiaa0763 | 961 |
| IPI00762033.1 | Q3UQN2 | K.VSIGNITLS*PAVSR.H | 387 | IGNITLSPAVSRH | F-BAR domain only protein 2 | Fcho2 | 809 |
| IPI00751153.1 | Q62441 | K.DAPIS*PASVASSSSTPSSK.S | 292 | KKDAPISPASVAS | Transducin-like enhancer protein 4 (Grg-4) (Groucho-related protein 4) | Tle4 Grg4 | 773 |
| IPI00311069.3 | Q8R4F1 | R.YPS*PLEANITLSWNK.S | 117 | TWSRYPSPLEANI | Netrin-G2 (Laminet-2) | Ntng2 Lmnt2 | 589 |
| IPI00421085.1 | Q6NVF9 | R.T*PLSEAEFEEIMNR.N | 407 | EMDTARTPLSEAE | Cleavage and polyadenylation specificity factor subunit 6 | Cpsf6 | 551 |
| IPI00118895.1 | Q61235 | R.SPS*LGSDLTFATR.T | 375 | SGCRSPSLGSDLT | Beta-2-syntrophin (59 kDa dystrophin-associated protein A1 basic component 2) (Syntrophin-3) (SNT3) (Syntrophin-like) (SNTL) | Sntb2 Snt2b2 | 520 |
| IPI00380392.1 | | K.TLQPSNPTEGSNPDTLFQFS*D.- | |  |  |  |  |
| IPI00459268.2 | Q8C0P0 | R.T*PGQVLSLISSLGFFT*PVGEK.D | 206 | KQDYSRTPGQVLS | Serine/threonine-protein kinase greatwall (GW) (GWL) (EC 2.7.11.1) (Microtubule-associated serine/threonine-protein kinase-like) (MAST-L) | Mastl Gw Gwl | 865 |
| IPI00556768.1 | Q569Z6 | R.IDIS*PSTFR.K | 679 | HRRIDISPSTFRK | Thyroid hormone receptor-associated protein 3 (Thyroid hormone receptor-associated protein complex 150 kDa component) (Trap150) | Thrap3 Trap150 | 951 |
| IPI00556768.1 | Q569Z6 | R.IDISPST*FR.K | 682 | IDISPSTFRKHGL | Thyroid hormone receptor-associated protein 3 (Thyroid hormone receptor-associated protein complex 150 kDa component) (Trap150) | Thrap3 Trap150 | 951 |
| IPI00230479.3 | Q61037 | K.SSS*SPELQTLQDILGDLGDK.I | 1388 | PLSKSSSSPELQT | Tuberin (Tuberous sclerosis 2 protein homolog) | Tsc2 | 1814 |
| IPI00230479.3 | Q61037 | K.SSSS*PELQTLQDILGDLGDK.I | 1389 | LSKSSSSPELQTL | Tuberin (Tuberous sclerosis 2 protein homolog) | Tsc2 | 1814 |
| IPI00230479.3 | Q61037 | R.SQS*GILDGEAATWSATGEESR.I | 1421 | AKVRSQSGILDGE | Tuberin (Tuberous sclerosis 2 protein homolog) | Tsc2 | 1814 |
| IPI00670545.2 | Q6ZQ58 | K.GLSAS*LPDLDSESWIEVK.K | 525 | PKGLSASLPDLDS | La-related protein 1 (La ribonucleoprotein domain family member 1) | Larp1 Kiaa0731 Larp | 1072 |
| IPI00670545.2 | Q6ZQ58 | K.GLS*ASLPDLDSESWIEVK.K | 523 | TLPKGLSASLPDL | La-related protein 1 (La ribonucleoprotein domain family member 1) | Larp1 Kiaa0731 Larp | 1072 |
| IPI00670545.2 | Q6ZQ58 | R.SLPTTVPES*PNYR.N | 751 | PTTVPESPNYRNA | La-related protein 1 (La ribonucleoprotein domain family member 1) | Larp1 Kiaa0731 Larp | 1072 |
| IPI00462728.2 | D3YTT6 | R.TYS*APAINAIQGGAFESPK.K | 250 | VLHRTYSAPAINA | Oxysterol-binding protein | Osbpl3 | 886 |
| IPI00652958.1 | Q80XQ2 | K.TISSS*PSIESLPGGR.E | 563 | SKTISSSPSIESL | TBC1 domain family member 5 | Tbc1d5 | 815 |
| IPI00652958.1 | Q80XQ2 | K.TISSSPS*IESLPGGR.E | 565 | TISSSPSIESLPG | TBC1 domain family member 5 | Tbc1d5 | 815 |
| IPI00406091.1 | Q9ERH4 | R.TSIAVIT*PFK.L | 323 | TSIAVITPFKLMT | Nucleolar and spindle-associated protein 1 (NuSAP) | Nusap1 | 427 |
| IPI00119242.1 | Q6ZQ03 | K.ATGGLCLLGAYADS*DDDESDVSEK.T | 120 | LGAYADSDDDESD | Formin-binding protein 4 (Formin-binding protein 30) | Fnbp4 Fbp30 Kiaa1014 | 1031 |
| IPI00474721.1 | Q8K4L0 | R.ALPSFPTSECVS*DVEPDTR.E | 74 | PTSECVSDVEPDT | ATP-dependent RNA helicase DDX54 (EC 3.6.4.13) (DEAD box protein 54) | Ddx54 | 874 |
| IPI00273851.2 | Q8C0V0 | K.ISDYFEYQGGNGS*SPVR.G | 158 | YQGGNGSSPVRGI | Serine/threonine-protein kinase tousled-like 1 (EC 2.7.11.1) (Tousled-like kinase 1) | Tlk1 | 766 |
| IPI00273851.2 | Q8C0V0 | K.ISDYFEYQGGNGSS*PVR.G | 159 | QGGNGSSPVRGIP | Serine/threonine-protein kinase tousled-like 1 (EC 2.7.11.1) (Tousled-like kinase 1) | Tlk1 | 766 |
| IPI00407698.1 | Q8BL80 | R.TS*SLDGPAAAVLSR.T | 396 | LPTHRTSSLDGPA | Rho GTPase-activating protein 22 (Rho-type GTPase-activating protein 22) (p68RacGAP) | Arhgap22 | 702 |
| IPI00785240.1 | Q8BTI8 | R.SS*SPVTELTARS*PVK.Q | 1067 | GSLSRSSSPVTEL | Serine/arginine repetitive matrix protein 2 | Srrm2 Kiaa0324 | 2703 |
| IPI00785240.1 | Q8BTI8 | K.NSGPVSEVNTGFS*PEVK.E | 1305 | EVNTGFSPEVKEE | Serine/arginine repetitive matrix protein 2 | Srrm2 Kiaa0324 | 2703 |
| IPI00785240.1 | Q8BTI8 | K.VS*SPVLETVQQR.T | 1359 | FSSQKVSSPVLET | Serine/arginine repetitive matrix protein 2 | Srrm2 Kiaa0324 | 2703 |
| IPI00785240.1 | Q8BTI8 | K.VSS*PVLETVQQR.T | 1360 | SSQKVSSPVLETV | Serine/arginine repetitive matrix protein 2 | Srrm2 Kiaa0324 | 2703 |
| IPI00785240.1 | Q8BTI8 | K.RVPS*PTPVPK.E | 2535 | ALKRVPSPTPVPK | Serine/arginine repetitive matrix protein 2 | Srrm2 Kiaa0324 | 2703 |
| IPI00621729.2 | Q01320 | K.FTVDLDS*DEDFS*GLDEK.D | 1328 | FTVDLDSDEDFSG | DNA topoisomerase 2-alpha (EC 5.99.1.3) (DNA topoisomerase II, alpha isozyme) | Top2a Top-2 Top2 | 1528 |
| IPI00621729.2 | Q01320 | K.DEDEDFLPLDAT*PPK.A | 1350 | FLPLDATPPKAKI | DNA topoisomerase 2-alpha (EC 5.99.1.3) (DNA topoisomerase II, alpha isozyme) | Top2a Top-2 Top2 | 1528 |
| IPI00671530.2 | Q80TV8 | R.SRS*DIDVNAAASAK.S | 600 | SLQRSRSDIDVNA | CLIP-associating protein 1 (Cytoplasmic linker-associated protein 1) | Clasp1 Kiaa0622 | 1535 |
| IPI00671530.2 | Q80TV8 | R.VLSTS*TDLEAAVADALK.K | 794 | MRVLSTSTDLEAA | CLIP-associating protein 1 (Cytoplasmic linker-associated protein 1) | Clasp1 Kiaa0622 | 1535 |
| IPI00671530.2 | Q80TV8 | R.VLST*STDLEAAVADALK.K | 793 | AMRVLSTSTDLEA | CLIP-associating protein 1 (Cytoplasmic linker-associated protein 1) | Clasp1 Kiaa0622 | 1535 |
| IPI00671530.2 | Q80TV8 | R.VLSTST*DLEAAVADALK.K | 795 | RVLSTSTDLEAAV | CLIP-associating protein 1 (Cytoplasmic linker-associated protein 1) | Clasp1 Kiaa0622 | 1535 |
| IPI00228810.2 | Q9EPL0 | K.CEIVGKDALS*ALARAS*TK.Q | 171 | VGKDALSALARAS | Xylosyltransferase 2 (EC 2.4.2.26) (Peptide O-xylosyltransferase 2) (Xylosyltransferase II) | Xylt2 | 865 |
| IPI00230719.7 | P28028 | R.AGFQTEDFSLYACAS*PK.T | 787 | SLYACASPKTPIQ | Serine/threonine-protein kinase B-raf (EC 2.7.11.1) (Proto-oncogene B-Raf) | Braf B-raf | 804 |
| IPI00473268.2 | Q61624 | K.GGLLTSEEDSGFSTS*PK.D | 306 | DSGFSTSPKDNSL | Zinc finger protein 148 (Beta enolase repressor factor 1) (G-rich box-binding protein) (Transcription factor BFCOL1) (Transcription factor ZBP-89) (Zinc finger DNA-binding protein 89) | Znf148 Zbp89 Zfp148 | 794 |
| IPI00165794.1 | Q8VCB1 | R.ASGSLVDT*SY*KTLR.F | 624 | SGSLVDTSYKTLR | Nucleoporin NDC1 (Transmembrane protein 48) | Ndc1 Tmem48 | 673 |
| IPI00652440.1 | P70419 | K.TPTFAGGLFS*ISK.K | 368 | FAGGLFSISKKYF | Polypeptide N-acetylgalactosaminyltransferase 3 (EC 2.4.1.41) (Polypeptide GalNAc transferase 3) (GalNAc-T3) (pp-GaNTase 3) (Protein-UDP acetylgalactosaminyltransferase 3) (UDP-GalNAc:polypeptide N-acetylgalactosaminyltransferase 3) | Galnt3 | 633 |
| IPI00331135.3 | Q63943 | R.LLS*PQQPALQR.N | 180 | TDPRLLSPQQPAL | Myocyte-specific enhancer factor 2D | Mef2d | 514 |
| IPI00758246.1 | Q69ZW3 | R.TSGS*DDPGLSSSTDSAQALASLGK.K | 676 | SARTSGSDDPGLS | EH domain-binding protein 1 | Ehbp1 Kiaa0903 | 1231 |
| IPI00755161.1 | Q3B7Z2 | R.TGS*NISGASSDVSLDEQYK.H | 377 | GHKRTGSNISGAS | Oxysterol-binding protein 1 | Osbp Kiaa4220 | 805 |
| IPI00230631.4 | O70293 | K.DVLDIEQFS*TVK.G | 484 | LDIEQFSTVKGVD | G protein-coupled receptor kinase 6 (EC 2.7.11.16) (G protein-coupled receptor kinase GRK6) | Grk6 Gprk6 | 576 |
| IPI00649501.1 | Q8K2Y9 | R.GIITDS*FGR.H | 393 | RGIITDSFGRHRR | Cerebral cavernous malformations protein 2 homolog (Malcavernin) (Osmosensing scaffold for MEKK3) | Ccm2 Osm | 453 |
| IPI00659672.1 | | K.DAQS*PLLEVDAASVK.Y | |  |  |  |  |
| IPI00659672.1 | | R.SCDYQFPSS*PSTDTLK.G | |  |  |  |  |
| IPI00758171.1 | O54826 | K.SFDNS*PGELGSSSLPTAGYK.R | 436 | PKSFDNSPGELGS | Protein AF-10 | Mllt10 Af10 | 1068 |
| IPI00467354.3 | Q499E5 | K.TRST*TLRRAWPSS*DFS*DR.A | 7 | MKKTRSTTLRRAW | Storkhead-box protein 2 | Stox2 Kiaa1392 | 926 |
| IPI00651897.1 | P49452 | K.ASFLDDFTESLTSST*QK.K | 61 | ESLTSSTQKKKAN | Centromere protein C (CENP-C) (Centromere autoantigen C) (Centromere protein C 1) (CENP-C 1) | Cenpc Cenpc1 | 906 |
| IPI00753582.1 | | K.T*IQFVNPAGDLVNMNEK.K | |  |  |  |  |
| IPI00406371.3 | P47856 | R.VDS*TTCLFPVEEK.A | 259 | GLSRVDSTTCLFP | Glutamine--fructose-6-phosphate aminotransferase [isomerizing] 1 (EC 2.6.1.16) (D-fructose-6-phosphate amidotransferase 1) (Glutamine:fructose-6-phosphate amidotransferase 1) (GFAT 1) (GFAT1) (Hexosephosphate aminotransferase 1) | Gfpt1 Gfpt | 697 |
| IPI00671412.2 | Q69ZZ6 | K.ALGVISNFQSS*PK.Y | 410 | ISNFQSSPKYGSE | Transmembrane and coiled-coil domains protein 1 | Tmcc1 Kiaa0779 | 649 |
| IPI00553798.2 | E9Q616 | K.GPSFNVAS*PESDFGVSLK.G | 5325 | PSFNVASPESDFG | AHNAK nucleoprotein (desmoyokin) | Ahnak | 5656 |
| IPI00553798.2 | E9Q616 | K.GGVTGS*PEASISGS*KGDLK.S | 5504 | KGGVTGSPEASIS | AHNAK nucleoprotein (desmoyokin) | Ahnak | 5656 |
| IPI00553798.2 | E9Q616 | K.ASLGSLEGEVEAEAS*SPK.G | 5535 | EVEAEASSPKGKF | AHNAK nucleoprotein (desmoyokin) | Ahnak | 5656 |
| IPI00553798.2 | E9Q616 | K.ASLGSLEGEVEAEASS*PK.G | 5536 | VEAEASSPKGKFS | AHNAK nucleoprotein (desmoyokin) | Ahnak | 5656 |
| IPI00553798.2 | E9Q616 | K.ASLGS*LEGEVEAEASSPK.G | 5525 | SKASLGSLEGEVE | AHNAK nucleoprotein (desmoyokin) | Ahnak | 5656 |
| IPI00553798.2 | E9Q616 | K.AS*LGSLEGEVEAEASSPK.G | 5522 | LKSSKASLGSLEG | AHNAK nucleoprotein (desmoyokin) | Ahnak | 5656 |
| IPI00553798.2 | E9Q616 | K.AS*LGSLEGEVEAEAS*SPK.G | 5522 | LKSSKASLGSLEG | AHNAK nucleoprotein (desmoyokin) | Ahnak | 5656 |
| IPI00553798.2 | E9Q616 | K.ASLGS*LEGEVEAEASS*PK.G | 5525 | SKASLGSLEGEVE | AHNAK nucleoprotein (desmoyokin) | Ahnak | 5656 |
| IPI00553798.2 | E9Q616 | K.AS*LGS*LEGEVEAEASSPK.G | 5522 | LKSSKASLGSLEG | AHNAK nucleoprotein (desmoyokin) | Ahnak | 5656 |
| IPI00553798.2 | E9Q616 | K.AS*LGSLEGEVEAEASS*PK.G | 5522 | LKSSKASLGSLEG | AHNAK nucleoprotein (desmoyokin) | Ahnak | 5656 |
| IPI00553798.2 | E9Q616 | K.ASLGS*LEGEVEAEAS*SPK.G | 5525 | SKASLGSLEGEVE | AHNAK nucleoprotein (desmoyokin) | Ahnak | 5656 |
| IPI00553798.2 | E9Q616 | K.AS*LGS*LEGEVEAEASS*PK.G | 5522 | LKSSKASLGSLEG | AHNAK nucleoprotein (desmoyokin) | Ahnak | 5656 |
| IPI00553798.2 | E9Q616 | R.EFSAPSTPTGT*LEFAGGDAK.G | 5571 | PSTPTGTLEFAGG | AHNAK nucleoprotein (desmoyokin) | Ahnak | 5656 |
| IPI00553798.2 | E9Q616 | R.EFSAPSTPT*GTLEFAGGDAK.G | 5569 | SAPSTPTGTLEFA | AHNAK nucleoprotein (desmoyokin) | Ahnak | 5656 |
| IPI00553798.2 | E9Q616 | R.EFS*APSTPTGTLEFAGGDAK.G | 5563 | SDEREFSAPSTPT | AHNAK nucleoprotein (desmoyokin) | Ahnak | 5656 |
| IPI00553798.2 | E9Q616 | R.EFS*APS*TPTGTLEFAGGDAK.G | 5563 | SDEREFSAPSTPT | AHNAK nucleoprotein (desmoyokin) | Ahnak | 5656 |
| IPI00553798.2 | E9Q616 | R.EFS*APST*PTGTLEFAGGDAK.G | 5563 | SDEREFSAPSTPT | AHNAK nucleoprotein (desmoyokin) | Ahnak | 5656 |
| IPI00553798.2 | E9Q616 | R.EFS*APSTPTGT*LEFAGGDAK.G | 5563 | SDEREFSAPSTPT | AHNAK nucleoprotein (desmoyokin) | Ahnak | 5656 |
| IPI00553798.2 | E9Q616 | K.FGT*FGGLGSK.S | 5590 | GKLKFGTFGGLGS | AHNAK nucleoprotein (desmoyokin) | Ahnak | 5656 |
| IPI00553798.2 | E9Q616 | K.GHYEVTGS*DDEAGK.L | 5607 | HYEVTGSDDEAGK | AHNAK nucleoprotein (desmoyokin) | Ahnak | 5656 |
| IPI00553798.2 | E9Q616 | K.GHYEVT*GSDDEAGK.L | 5605 | KGHYEVTGSDDEA | AHNAK nucleoprotein (desmoyokin) | Ahnak | 5656 |
| IPI00467789.3 | B2RRD7 | R.GAGWLS*EDEDSPLDALDLVWAK.C | 1074 | RGAGWLSEDEDSP | Peregrin (Bromodomain and PHD finger-containing protein 1) | Brpf1 | 1212 |
| IPI00669126.2 | | K.Y*NDEVDVVAS*RMFLENK.T | |  |  |  |  |
| IPI00226729.1 | Q8BXK8 | R.YLTGTYVQEES*PEGGR.F | 101 | TYVQEESPEGGRF | Arf-GAP with GTPase, ANK repeat and PH domain-containing protein 1 (AGAP-1) (Centaurin-gamma-2) (Cnt-g2) | Agap1 Centg2 Kiaa1099 | 857 |
| IPI00226439.3 | P70121 | K.RIT*VHHNSAEGTS*EEK.E | 197 | VENKRITVHHNSA | Zinc fingers and homeoboxes protein 1 | Zhx1 | 873 |
| IPI00229072.1 | Q60953 | R.LATSS*PEQSWPSTFK.A | 515 | DRLATSSPEQSWP | Protein PML | Pml | 885 |
| IPI00757040.2 | Q80XI6 | R.NVFEVGAGDSPT*FPR.F | 527 | GAGDSPTFPRFRA | Mitogen-activated protein kinase kinase kinase 11 (EC 2.7.11.25) (Mixed lineage kinase 3) | Map3k11 Mlk3 | 850 |
| IPI00654283.1 | O88874 | K.KPS*PQPS*PPR.Q | 325 | QQPKKPSPQPSPP | Cyclin-K | Ccnk | 554 |
| IPI00403031.4 | Q8K124 | R.SSS*LGDLLR.E | 395 | FHPRSSSLGDLLR | Pleckstrin homology domain-containing family O member 2 (PH domain-containing family O member 2) (Pleckstrin homology domain-containing family Q member 1) (PH domain-containing family Q member 1) | Plekho2 Plekhq1 | 495 |
| IPI00658532.2 | Q8CHG7 | R.YS*IPDLAVDVEQVIGLEK.V | 501 | KKASRYSIPDLAV | Rap guanine nucleotide exchange factor 2 (Cyclic nucleotide ras GEF) (CNrasGEF) (Neural RAP guanine nucleotide exchange protein) (nRap GEP) (PDZ domain-containing guanine nucleotide exchange factor 1) (PDZ-GEF1) (RA-GEF-1) (Ras/Rap1-associating GEF-1) | Rapgef2 Kiaa0313 Pdzgef1 | 1496 |
| IPI00658532.2 | Q8CHG7 | K.DLPPFGINS*PQALK.K | 1079 | PPFGINSPQALKK | Rap guanine nucleotide exchange factor 2 (Cyclic nucleotide ras GEF) (CNrasGEF) (Neural RAP guanine nucleotide exchange protein) (nRap GEP) (PDZ domain-containing guanine nucleotide exchange factor 1) (PDZ-GEF1) (RA-GEF-1) (Ras/Rap1-associating GEF-1) | Rapgef2 Kiaa0313 Pdzgef1 | 1496 |
| IPI00461860.3 | Q62018 | K.GGEFDEFVNDDT*DDDLPVSK.K | 925 | EFVNDDTDDDLPV | RNA polymerase-associated protein CTR9 homolog (SH2 domain-binding protein 1) (Tetratricopeptide repeat-containing, SH2-binding phosphoprotein of 150 kDa) (TPR-containing, SH2-binding phosphoprotein of 150 kDa) (p150TSP) | Ctr9 Kiaa0155 Sh2bp1 | 1173 |
| IPI00123868.2 | Q9WTU0 | K.DSDYVYPSLES*DEDNPVFK.S | 876 | VYPSLESDEDNPV | Lysine-specific demethylase PHF2 (EC 1.14.11.-) (GRC5) (PHD finger protein 2) | Phf2 Kiaa0662 | 1096 |
| IPI00757089.1 | E9Q6P5 | R.VEQALSEVASS*LQSSAPK.Q | 678 | LSEVASSLQSSAP | Tetratricopeptide repeat protein 7B (TPR repeat protein 7B) | Ttc7b | 843 |
| IPI00387213.1 | P35831 | K.S*PAEVTDIGFGNR.C | 748 | KDQITKSPAEVTD | Tyrosine-protein phosphatase non-receptor type 12 (EC 3.1.3.48) (MPTP-PEST) (Protein-tyrosine phosphatase P19) (P19-PTP) | Ptpn12 | 775 |
| IPI00387462.1 | O88942 | R.AS*ITEESWLGAR.G | 247 | STSPRASITEESW | Nuclear factor of activated T-cells, cytoplasmic 1 (NF-ATc1) (NFATc1) (NFAT transcription complex cytosolic component) (NF-ATc) (NFATc) | Nfatc1 Nfat2 Nfatc | 717 |
| IPI00317794.4 | P09405 | K.NLS*FNITEDELK.E | 403 | LLAKNLSFNITED | Nucleolin (Protein C23) | Ncl Nuc | 707 |
| IPI00126338.4 | Q61033 | R.LVSAAAS*PSLIR.E | 308 | LVSAAASPSLIRE | Lamina-associated polypeptide 2, isoforms alpha/zeta (Thymopoietin isoforms alpha/zeta) (TP alpha/zeta) | Tmpo Lap2 | 693 |
| IPI00319524.7 | Q920B0 | R.S*LDDIAMDLTETGTQR.G | 372 | KIPSARSLDDIAM | FERM domain-containing protein 4B (GRP1-binding protein GRSP1) (Golgi-associated band 4.1-like protein) (GOBLIN) | Frmd4b | 1035 |
| IPI00652738.2 | Q61846 | K.GTNVFGS*LER.G | 521 | GTNVFGSLERGLD | Maternal embryonic leucine zipper kinase (EC 2.7.11.1) (Protein kinase PK38) (mPK38) (Tyrosine-protein kinase MELK) (EC 2.7.10.2) | Melk Kiaa0175 Pk38 | 643 |
| IPI00399498.1 | Q8CH77 | K.SLT*NLSFLTDSEK.K | 95 | RKQKSLTNLSFLT | Neuron navigator 1 (Pore membrane and/or filament-interacting-like protein 3) | Nav1 Kiaa1151 Pomfil3 | 1875 |
| IPI00399498.1 | Q8CH77 | K.S*LTNLSFLTDSEK.K | 93 | NLRKQKSLTNLSF | Neuron navigator 1 (Pore membrane and/or filament-interacting-like protein 3) | Nav1 Kiaa1151 Pomfil3 | 1875 |
| IPI00399498.1 | Q8CH77 | R.SLAESGLNWFS*ESEEK.T | 477 | SGLNWFSESEEKT | Neuron navigator 1 (Pore membrane and/or filament-interacting-like protein 3) | Nav1 Kiaa1151 Pomfil3 | 1875 |
| IPI00664790.2 | E9PWW9 | K.VGS*PLDYSLVDLPSTNGQS*PGK.A | 1359 | FENKVGSPLDYSL | Remodeling and spacing factor 1 | Rsf1 | 1441 |
| IPI00625058.2 | Q8CGE9 | R.S*LDDLESATVSDGELTGADLK.D | 661 | RFSITRSLDDLES | Regulator of G-protein signaling 12 (RGS12) | Rgs12 | 1381 |
| IPI00121418.1 | P13405 | R.ILVSIGESFGT*SEK.F | 834 | IGESFGTSEKFQK | Retinoblastoma-associated protein (pRb) (Rb) (pp105) | Rb1 Rb-1 | 921 |
| IPI00312116.4 | P35991 | K.LSYY*EYDFER.G | 40 | VHKLSYYEYDFER | Tyrosine-protein kinase BTK (EC 2.7.10.2) (Agammaglobulinemia tyrosine kinase) (ATK) (B-cell progenitor kinase) (BPK) (Bruton tyrosine kinase) (Kinase EMB) | Btk Bpk | 659 |
| IPI00626851.1 | Q9R0G7 | R.AYLQSITPQGYS*DSEER.E | 1122 | ITPQGYSDSEERE | Zinc finger E-box-binding homeobox 2 (Smad-interacting protein 1) (Zinc finger homeobox protein 1b) | Zeb2 Sip1 Zfhx1b Zfx1b | 1215 |
| IPI00128218.2 | B1AR09 | K.GSSLQSS*PDFAAFPK.L | 340 | GSSLQSSPDFAAF | Myeloid/lymphoid or mixed-lineage leukemia; translocated to, 6 | Mllt6 | 1079 |
| IPI00453800.4 | Q8K327 | K.SSCGS*PPDLWK.S | 464 | QKSSCGSPPDLWK | Chromosome alignment-maintaining phosphoprotein 1 (Zinc finger protein 828) | Champ1 D8Ertd457e Kiaa1802 Zfp828 Znf828 | 802 |
| IPI00330606.4 | Q80U28 | R.ATLS*DSEIETNSATSAIFGK.A | 1237 | SSRATLSDSEIET | MAP kinase-activating death domain protein (Rab3 GDP/GTP exchange factor) | Madd Kiaa0358 | 1577 |
| IPI00461237.1 | O70305 | R.TS*PAGGTWSSVVSGVPR.L | 636 | PPVARTSPAGGTW | Ataxin-2 (Spinocerebellar ataxia type 2 protein homolog) | Atxn2 Atx2 Sca2 | 1285 |
| IPI00408190.1 | O08784 | K.AGAVTSSASLSS*PALAK.G | 593 | SSASLSSPALAKG | Treacle protein (Treacher Collins syndrome protein homolog) | Tcof1 | 1320 |
| IPI00622815.1 | Q61165 | R.IGS*DPLAYEPK.A | 707 | SRARIGSDPLAYE | Sodium/hydrogen exchanger 1 (Na(+)/H(+) exchanger 1) (NHE-1) (Solute carrier family 9 member 1) | Slc9a1 Nhe1 | 820 |
| IPI00676937.2 | Q8BZN6 | K.GNFNST*VNNTVTVR.S | 196 | KGNFNSTVNNTVT | Dedicator of cytokinesis protein 10 (Zizimin-3) | Dock10 Kiaa0694 Ziz3 | 2150 |
| IPI00676937.2 | Q8BZN6 | K.DLYPFTVNTS*NQGSR.D | 1232 | PFTVNTSNQGSRD | Dedicator of cytokinesis protein 10 (Zizimin-3) | Dock10 Kiaa0694 Ziz3 | 2150 |
| IPI00551367.1 | P70388 | K.LFDVCGS*QDLESDLGR.L | 635 | LFDVCGSQDLESD | DNA repair protein RAD50 (mRad50) (EC 3.6.-.-) | Rad50 | 1312 |
| IPI00420949.2 | | R.AYQHGGVTGLSQY*.- |  |  |  |  |  |
| IPI00411097.1 | Q6NV83 | K.LYSILQGDS*PTK.W | 485 | SILQGDSPTKWRT | U2 snRNP-associated SURP motif-containing protein (140 kDa Ser/Arg-rich domain protein) (U2-associated protein SR140) | U2surp Sr140 | 1029 |
| IPI00411097.1 | Q6NV83 | K.LYSILQGDSPT*K.W | 487 | LQGDSPTKWRTED | U2 snRNP-associated SURP motif-containing protein (140 kDa Ser/Arg-rich domain protein) (U2-associated protein SR140) | U2surp Sr140 | 1029 |
| IPI00661969.1 | | R.SLS*PGGAALGYR.D |  |  |  |  |  |
| IPI00750715.2 | Q80UU1 | R.LWDIPLT*T*R.R | 351 | LWDIPLTTRRPTF | Ankyrin repeat and zinc finger domain-containing protein 1 | Ankzf1 D1Ertd161e | 748 |
| IPI00625056.1 | | R.TSS*EDNLYLAVLR.A |  |  |  |  |  |
| IPI00625056.1 | | R.SS*SVGSSSSYPISSAGPR.T | |  |  |  |  |
| IPI00625056.1 | | R.SSS*VGSSSSYPISSAGPR.T | |  |  |  |  |
| IPI00625056.1 | | K.GYYSPYSVSGSGST*AGSR.T | |  |  |  |  |
| IPI00625056.1 | | K.GYYS*PYSVSGSGSTAGSR.T | |  |  |  |  |
| IPI00625056.1 | | K.GYYSPYSVSGSGS*TAGSR.T | |  |  |  |  |
| IPI00471007.1 | E9Q0A3 | R.LST*GSFPEDLLESDSSR.S | 591 | GTQRLSTGSFPED | Rho guanine nucleotide exchange factor (GEF) 11 (Fragment) | Arhgef11 | 1475 |
| IPI00755614.1 | Q4QRL3 | R.VQS*SLCLGDETLAGGQR.R | 1384 | PMRRVQSSLCLGD | Coiled-coil domain-containing protein 88B (Gipie) (Hook-related protein 3) (HkRP3) | Ccdc88b Ccdc88 | 1481 |
| IPI00468120.1 | Q6PDH0 | R.LS*PAYSLGSLTGASPR.Q | 541 | SFSGRLSPAYSLG | Pleckstrin homology-like domain family B member 1 (Protein LL5-alpha) | Phldb1 Kiaa0638 Ll5a | 1371 |
| IPI00407863.5 | Q8BRT1 | R.S*RSDIDVNAAAGAK.A | 374 | PGSLQRSRSDIDV | CLIP-associating protein 2 (Cytoplasmic linker-associated protein 2) | Clasp2 Kiaa0627 | 1286 |
| IPI00407863.5 | Q8BRT1 | R.SRS*DIDVNAAAGAK.A | 376 | SLQRSRSDIDVNA | CLIP-associating protein 2 (Cytoplasmic linker-associated protein 2) | Clasp2 Kiaa0627 | 1286 |
| IPI00274516.1 | Q8BWJ3 | K.S*LNLVDSPQPLLK.T | 729 | LSGHRKSLNLVDS | Phosphorylase b kinase regulatory subunit alpha, liver isoform (Phosphorylase kinase alpha L subunit) | Phka2 | 1235 |
| IPI00620585.2 | P59672 | K.SPS*FASEWDEIEK.I | 679 | RLEKSPSFASEWD | Ankyrin repeat and SAM domain-containing protein 1A (Odin) | Anks1a Anks1 Kiaa0229 Odin | 1150 |
| IPI00461460.1 | A0A087WP65 | K.ASS*FSGISVLTR.G | 94 | AVTKASSFSGISV | R3H domain-containing 1 (Fragment) | R3hdm1 | 183 |
| IPI00623570.3 | G5E870 | R.SES*PPAELPSLR.R | 312 | TKKRSESPPAELP | E3 ubiquitin-protein ligase TRIP12 (EC 2.3.2.26) (HECT-type E3 ubiquitin transferase TRIP12) (Thyroid receptor-interacting protein 12) (TR-interacting protein 12) (TRIP-12) | Trip12 | 2025 |
| IPI00623570.3 | G5E870 | K.QLLLYLTS*K.N | 1206 | LLLYLTSKNEKDA | E3 ubiquitin-protein ligase TRIP12 (EC 2.3.2.26) (HECT-type E3 ubiquitin transferase TRIP12) (Thyroid receptor-interacting protein 12) (TR-interacting protein 12) (TRIP-12) | Trip12 | 2025 |
| IPI00135132.1 | Q9QXL1 | K.AVS*AECLGPPLDSSTK.N | 1150 | AQMKAVSAECLGP | Kinesin-like protein KIF21B (Kinesin-like protein KIF6) | Kif21b Kif6 | 1668 |
| IPI00719994.2 | | R.NLS*GSTLYPVSNIPR.S | |  |  |  |  |
| IPI00338954.3 | P59808 | R.TCS*FGGFDLTNR.S | 400 | SHGRTCSFGGFDL | SAM and SH3 domain-containing protein 1 | Sash1 | 1230 |
| IPI00652634.1 | Q64700 | K.IFYYFSNS*PSK.R | 1076 | FYYFSNSPSKRLR | Retinoblastoma-like protein 2 (130 kDa retinoblastoma-associated protein) (p130) (Retinoblastoma-related protein 2) (RBR-2) (pRb2) | Rbl2 | 1135 |
| IPI00337844.4 | Q9ERU9 | R.YIASVQGSAPS*PR.E | 21 | VQGSAPSPREKSM | E3 SUMO-protein ligase RanBP2 (EC 2.3.2.-) (Ran-binding protein 2) (RanBP2) | Ranbp2 | 3053 |
| IPI00337844.4 | Q9ERU9 | R.FGESTTGFNFS*FK.S | 2107 | TTGFNFSFKSALS | E3 SUMO-protein ligase RanBP2 (EC 2.3.2.-) (Ran-binding protein 2) (RanBP2) | Ranbp2 | 3053 |
| IPI00117274.1 | Q8C3J5 | K.VEEEPISPGS*TLPEVK.L | 1686 | EPISPGSTLPEVK | Dedicator of cytokinesis protein 2 (Protein Hch) | Dock2 | 1828 |
| IPI00622008.2 | Q60592 | R.NQSLGQS*APSLTAGLK.E | 88 | NQSLGQSAPSLTA | Microtubule-associated serine/threonine-protein kinase 2 (EC 2.7.11.1) | Mast2 Mast205 | 1734 |
| IPI00453689.1 | Q8C0T5 | K.GS*GFSLDVIDGPISQR.E | 255 | TGGGKGSGFSLDV | Signal-induced proliferation-associated 1-like protein 1 (SIPA1-like protein 1) | Sipa1l1 Kiaa0440 | 1782 |
| IPI00225306.2 | Q8BTY2 | R.GS*LLQIPVK.T | 960 | LPFERGSLLQIPV | Sodium bicarbonate cotransporter 3 (Solute carrier family 4 member 7) | Slc4a7 Nbc3 | 1034 |
| IPI00319830.7 | Q62261 | R.AQT*LPTSVVTITSESSPGK.R | 2327 | ASSRAQTLPTSVV | Spectrin beta chain, non-erythrocytic 1 (Beta-II spectrin) (Embryonic liver fodrin) (Fodrin beta chain) | Sptbn1 Elf Spnb-2 Spnb2 Sptb2 | 2363 |
| IPI00410738.3 | Q99PP2 | R.EEGPLS*PFLGQLDEDYR.T | 246 | REEGPLSPFLGQL | Zinc finger protein 318 (Testicular zinc finger protein) | Znf318 Tzf Zfp318 | 2237 |
| IPI00229801.5 | P70399 | R.STPFIVPSSPT*EQGGR.K | 384 | IVPSSPTEQGGRK | TP53-binding protein 1 (53BP1) (p53-binding protein 1) (p53BP1) | Tp53bp1 Trp53bp1 | 1969 |
| IPI00515528.2 | E9Q784 | K.AAVVAS*PLLDQQR.N | 242 | KAAVVASPLLDQQ | Zinc finger CCCH domain-containing protein 13 | Zc3h13 | 1729 |
| IPI00622645.2 | A2AHC3 | R.TS*PQAPGLVASIR.S | 560 | PQMPRTSPQAPGL | Calmodulin-regulated spectrin-associated protein 1 | Camsap1 | 1581 |
| IPI00120734.1 | Q9JIL5 | R.TLSDFNSLISS*PR.L | 1378 | FNSLISSPRLGRE | Tubby-related protein 4 (Tubby superfamily protein) (Tubby-like protein 4) | Tulp4 Tusp | 1547 |
| IPI00648909.1 | | R.S*NSAWQIYLQR.R |  |  |  |  |  |
| IPI00266988.6 | A2A480 | K.LNFDMTAS*PK.I | 401 | NFDMTASPKILLS | Zinc finger, MYND-type-containing 8 (Fragment) | Zmynd8 | 492 |
| IPI00462727.5 | O35071 | R.YPPYTT*PPR.M | 1080 | RYPPYTTPPRMRR | Kinesin-like protein KIF1C | Kif1c | 1100 |
| IPI00654074.2 | Q921C3 | K.LTS*DAEDLSLESVCTR.S | 1927 | DKMKLTSDAEDLS | Bromodomain and WD repeat-containing protein 1 (WD repeat-containing protein 9) | Brwd1 Wdr9 | 2304 |
| IPI00654344.1 | Q91VW5 | R.TSS*FTDQLDDVTPNR.E | 41 | TRSRTSSFTDQLD | Golgin subfamily A member 4 (tGolgin-1) | Golga4 | 2238 |
| IPI00122594.4 | Q8CJF7 | K.NLS*FDELYPLGAEK.L | 1541 | EEAKNLSFDELYP | Protein ELYS (Embryonic large molecule derived from yolk sac) (Protein MEL-28) (Putative AT-hook-containing transcription factor 1) | Ahctf1 Elys | 2243 |
| IPI00664305.1 | A2AIV2 | R.SFLSEPSS*PGR.S | 1578 | FLSEPSSPGRSKT | Protein virilizer homolog | Virma Kiaa1429 | 1811 |
| IPI00751623.1 | | R.LLIEGNY*K.A |  |  |  |  |  |
| IPI00322707.5 | Q61687 | K.LTPVSLS*NSPIK.G | 588 | LTPVSLSNSPIKG | Transcriptional regulator ATRX (EC 3.6.4.12) (ATP-dependent helicase ATRX) (HP1 alpha-interacting protein) (HP1-BP38 protein) (Heterochromatin protein 2) (X-linked nuclear protein) | Atrx Hp1bp2 Xnp | 2476 |
| IPI00310168.4 | Q9QX47 | K.ESAQAVAVALS*PK.E | 1723 | AVAVALSPKESSE | Protein SON (Negative regulatory element-binding protein) (NRE-binding protein) | Son Nrebp | 2444 |
| IPI00230618.1 | Q9Z1T6 | R.S*ASITNLSLDR.S | 305 | SPARNRSASITNL | 1-phosphatidylinositol 3-phosphate 5-kinase (Phosphatidylinositol 3-phosphate 5-kinase) (EC 2.7.1.150) (FYVE finger-containing phosphoinositide kinase) (PIKfyve) (Phosphatidylinositol 3-phosphate 5-kinase type III) (PIPkin-III) (Type III PIP kinase) (p235) | Pikfyve Kiaa0981 Pip5k3 | 2097 |
| IPI00754183.1 | E9PVX6 | K.LESIENLT*GLR.K | 1428 | ESIENLTGLRKQS | Proliferation marker protein Ki-67 (Antigen identified by monoclonal antibody Ki-67 homolog) (Antigen KI-67 homolog) (Antigen Ki67 homolog) | Mki67 | 3177 |
| IPI00421052.1 | Q6KCD5 | K.AITSLLGGGS*PK.N | 2652 | SLLGGGSPKNNTA | Nipped-B-like protein (Delangin homolog) (SCC2 homolog) | Nipbl Scc2 | 2798 |
| IPI00604969.3 | A2ASS6 | R.SDTGLY*S*IT*AVNNLGTASK.E | 17983 | RSDTGLYSITAVN | Titin (EC 2.7.11.1) (Connectin) | Ttn | 35213 |
| IPI00551236.2 | P54227 | R.AS*GQAFELILSPR.S | 16 | ELEKRASGQAFEL | Stathmin (Leukemia-associated gene protein) (Leukemia-associated phosphoprotein p18) (Metablastin) (Oncoprotein 18) (Op18) (Phosphoprotein p19) (pp19) (Prosolin) (Protein Pr22) (pp17) | Stmn1 Lag Lap18 Pr22 | 149 |
| IPI00551236.2 | P54227 | R.AS*GQAFELILS*PR.S | 16 | ELEKRASGQAFEL | Stathmin (Leukemia-associated gene protein) (Leukemia-associated phosphoprotein p18) (Metablastin) (Oncoprotein 18) (Op18) (Phosphoprotein p19) (pp19) (Prosolin) (Protein Pr22) (pp17) | Stmn1 Lag Lap18 Pr22 | 149 |
| IPI00331515.2 | P70257 | K.S*IDDSEMESPVDDVFYPGTGR.S | 280 | STKRPKSIDDSEM | Nuclear factor 1 X-type (NF1-X) (Nuclear factor 1/X) (CCAAT-box-binding transcription factor) (CTF) (Nuclear factor I/X) (NF-I/X) (NFI-X) (TGGCA-binding protein) | Nfix | 488 |
| IPI00331515.2 | P70257 | K.SIDDS*EMESPVDDVFYPGTGR.S | 284 | PKSIDDSEMESPV | Nuclear factor 1 X-type (NF1-X) (Nuclear factor 1/X) (CCAAT-box-binding transcription factor) (CTF) (Nuclear factor I/X) (NF-I/X) (NFI-X) (TGGCA-binding protein) | Nfix | 488 |
| IPI00331515.2 | P70257 | R.S*PAAGSSQSSGWPNDVDAGPASLK.K | 301 | YPGTGRSPAAGSS | Nuclear factor 1 X-type (NF1-X) (Nuclear factor 1/X) (CCAAT-box-binding transcription factor) (CTF) (Nuclear factor I/X) (NF-I/X) (NFI-X) (TGGCA-binding protein) | Nfix | 488 |
| IPI00331515.2 | P70257 | K.LDFCSALSSQGSS*PR.M | 341 | LSSQGSSPRMAFT | Nuclear factor 1 X-type (NF1-X) (Nuclear factor 1/X) (CCAAT-box-binding transcription factor) (CTF) (Nuclear factor I/X) (NF-I/X) (NFI-X) (TGGCA-binding protein) | Nfix | 488 |
| IPI00331515.2 | P70257 | K.LDFCSALSSQGS*SPR.M | 340 | ALSSQGSSPRMAF | Nuclear factor 1 X-type (NF1-X) (Nuclear factor 1/X) (CCAAT-box-binding transcription factor) (CTF) (Nuclear factor I/X) (NF-I/X) (NFI-X) (TGGCA-binding protein) | Nfix | 488 |
| IPI00474783.3 | Q5SWU9 | R.FIIGS*VSEDNS*EDEISNLVK.L | 23 | SRFIIGSVSEDNS | Acetyl-CoA carboxylase 1 (ACC1) (EC 6.4.1.2) (ACC-alpha) (Acetyl-CoA carboxylase 265) [Includes: Biotin carboxylase (EC 6.3.4.14)] | Acaca Acac Gm738 | 2345 |
| IPI00473984.1 | Q8BJ16 | R.ST*AQWFGVSGDWEGK.R | 44 | YLFCRSTAQWFGV | Inactive rhomboid protein | Rhbdf2 Rhbdl6 | 325 |
| IPI00473984.1 | Q8BJ16 | R.SFAYPS*FLEEDAVDGADTFDSSFFSK.E | 198 | RSFAYPSFLEEDA | Inactive rhomboid protein | Rhbdf2 Rhbdl6 | 325 |
| IPI00469331.2 | Q8R4R6 | R.SIYDDISS*PGLGSTPLTSR.R | 100 | IYDDISSPGLGST | Nucleoporin NUP35 (35 kDa nucleoporin) (Mitotic phosphoprotein 44) (MP-44) (Nuclear pore complex protein Nup53) (Nucleoporin NUP53) | Nup35 Mp44 Nup53 | 325 |
| IPI00469331.2 | Q8R4R6 | R.SIYDDISS*PGLGST*PLTSR.R | 100 | IYDDISSPGLGST | Nucleoporin NUP35 (35 kDa nucleoporin) (Mitotic phosphoprotein 44) (MP-44) (Nuclear pore complex protein Nup53) (Nucleoporin NUP53) | Nup35 Mp44 Nup53 | 325 |
| IPI00469331.2 | Q8R4R6 | R.GVLS*SPSLAFTT*PIR.T | 257 | SDRGVLSSPSLAF | Nucleoporin NUP35 (35 kDa nucleoporin) (Mitotic phosphoprotein 44) (MP-44) (Nuclear pore complex protein Nup53) (Nucleoporin NUP53) | Nup35 Mp44 Nup53 | 325 |
| IPI00469331.2 | Q8R4R6 | R.GVLSS*PSLAFTT*PIR.T | 258 | DRGVLSSPSLAFT | Nucleoporin NUP35 (35 kDa nucleoporin) (Mitotic phosphoprotein 44) (MP-44) (Nuclear pore complex protein Nup53) (Nucleoporin NUP53) | Nup35 Mp44 Nup53 | 325 |
| IPI00118849.1 | Q61214 | R.IYQY*IQSR.F | 321 | GQRIYQYIQSRFY | Dual specificity tyrosine-phosphorylation-regulated kinase 1A (EC 2.7.12.1) (Dual specificity YAK1-related kinase) (MP86) (Protein kinase minibrain homolog) (MNBH) | Dyrk1a Dyrk | 763 |
| IPI00420527.2 | | R.TLS*PSSGYSSQSGTPTLPPK.G | |  |  |  |  |
| IPI00420527.2 | | R.TLSPS*SGYSSQSGTPTLPPK.G | |  |  |  |  |
| IPI00420527.2 | | R.SPGASVSS*SLTSLCSSSSDPTPLDR.S | | |  |  |  |
| IPI00420527.2 | | K.VPAPFS*PPPSK.S |  |  |  |  |  |
| IPI00308854.3 | Q8BLK9 | R.TFGLS*LSTDSSAVGAVASDSEPSR.V | 196 | SRTFGLSLSTDSS | Ribosomal protein S6 kinase delta-1 (S6K-delta-1) (EC 2.7.11.1) (52 kDa ribosomal protein S6 kinase) | Rps6kc1 | 1056 |
| IPI00308854.3 | Q8BLK9 | K.GVDLLLEGVQGES*SPTR.R | 280 | EGVQGESSPTRRE | Ribosomal protein S6 kinase delta-1 (S6K-delta-1) (EC 2.7.11.1) (52 kDa ribosomal protein S6 kinase) | Rps6kc1 | 1056 |
| IPI00308854.3 | Q8BLK9 | K.GVDLLLEGVQGESS*PTR.R | 281 | GVQGESSPTRREA | Ribosomal protein S6 kinase delta-1 (S6K-delta-1) (EC 2.7.11.1) (52 kDa ribosomal protein S6 kinase) | Rps6kc1 | 1056 |
| IPI00308854.3 | Q8BLK9 | K.GVDLLLEGVQGESSPT*R.R | 283 | QGESSPTRREAVK | Ribosomal protein S6 kinase delta-1 (S6K-delta-1) (EC 2.7.11.1) (52 kDa ribosomal protein S6 kinase) | Rps6kc1 | 1056 |
| IPI00308854.3 | Q8BLK9 | R.VFSGGEDLEAVSS*PPTSESLSR.S | 577 | DLEAVSSPPTSES | Ribosomal protein S6 kinase delta-1 (S6K-delta-1) (EC 2.7.11.1) (52 kDa ribosomal protein S6 kinase) | Rps6kc1 | 1056 |
| IPI00308854.3 | Q8BLK9 | R.VFSGGEDLEAVS*SPPTSESLSR.S | 576 | EDLEAVSSPPTSE | Ribosomal protein S6 kinase delta-1 (S6K-delta-1) (EC 2.7.11.1) (52 kDa ribosomal protein S6 kinase) | Rps6kc1 | 1056 |
| IPI00308854.3 | Q8BLK9 | R.VFS*GGEDLEAVSS*PPTSESLSR.S | 567 | THLRVFSGGEDLE | Ribosomal protein S6 kinase delta-1 (S6K-delta-1) (EC 2.7.11.1) (52 kDa ribosomal protein S6 kinase) | Rps6kc1 | 1056 |
| IPI00308854.3 | Q8BLK9 | R.SS*PDQFLFSSLR.S | 779 | AVDHRSSPDQFLF | Ribosomal protein S6 kinase delta-1 (S6K-delta-1) (EC 2.7.11.1) (52 kDa ribosomal protein S6 kinase) | Rps6kc1 | 1056 |
| IPI00222531.6 | Q7TNF9 | R.VFEEAT*SPGPDLAFLTSCPDK.N | 353 | RVFEEATSPGPDL | Protein FAM117A | Fam117a | 451 |
| IPI00395068.3 | P19426 | K.S*LYESFVSSSDR.L | 131 | RRPQRKSLYESFV | Negative elongation factor E (NELF-E) (RNA-binding protein RD) | Nelfe D17h6s45 Rd Rdbp | 375 |
| IPI00395068.3 | P19426 | K.SLY*ESFVSSSDR.L | 133 | PQRKSLYESFVSS | Negative elongation factor E (NELF-E) (RNA-binding protein RD) | Nelfe D17h6s45 Rd Rdbp | 375 |
| IPI00274639.4 | Q9ESL4 | R.SDSS*ADCQWLDTLR.M | 568 | GSRSDSSADCQWL | Mitogen-activated protein kinase kinase kinase 20 (EC 2.7.11.25) (Human cervical cancer suppressor gene 4 protein) (HCCS-4) (Leucine zipper- and sterile alpha motif kinase ZAK) (Leucine zipper- and sterile alpha motif-containing kinase) (MLK-like mitogen-activated protein triple kinase) (Mitogen-activated protein kinase kinase kinase MLT) (Mixed lineage kinase-related kinase) (MLK-related kinase) (MRK) (Sterile alpha motif- and leucine zipper-containing kinase AZK) | Map3k20 Mltk Zak | 802 |
| IPI00274639.4 | Q9ESL4 | R.SQSNPILGS*PFFPYFANQDSYAAAVR.R | 599 | SNPILGSPFFPYF | Mitogen-activated protein kinase kinase kinase 20 (EC 2.7.11.25) (Human cervical cancer suppressor gene 4 protein) (HCCS-4) (Leucine zipper- and sterile alpha motif kinase ZAK) (Leucine zipper- and sterile alpha motif-containing kinase) (MLK-like mitogen-activated protein triple kinase) (Mitogen-activated protein kinase kinase kinase MLT) (Mixed lineage kinase-related kinase) (MLK-related kinase) (MRK) (Sterile alpha motif- and leucine zipper-containing kinase AZK) | Map3k20 Mltk Zak | 802 |
| IPI00169921.2 | Q8K2J7 | R.SLMSVS*GIESVNGDVPATPVK.R | 250 | RSLMSVSGIESVN | RELT-like protein 1 | Rell1 | 272 |
| IPI00653346.1 | | R.ELAGS*VESLPSSPWNS*PR.V | |  |  |  |  |
| IPI00480432.2 | Q8R361 | K.YDLESAS*AILPSSALEDPELGSLGK.M | 209 | YDLESASAILPSS | Rab11 family-interacting protein 5 (Rab11-FIP5) (Rab11-interacting protein Rip11) | Rab11fip5 D6Ertd32e Rip11 | 645 |
| IPI00480432.2 | Q8R361 | R.TS*LSTALSSGLER.L | 539 | RKQPRTSLSTALS | Rab11 family-interacting protein 5 (Rab11-FIP5) (Rab11-interacting protein Rip11) | Rab11fip5 D6Ertd32e Rip11 | 645 |
| IPI00652438.2 | A6H5X4 | R.LVDETASES*DYEGIETLLFDCGLFK.D | 240 | DETASESDYEGIE | PHD finger protein 11 (PHD finger protein 11-like) (PHD finger protein 11D) | Phf11 D14Ertd668e Phf11d Phf11l | 337 |
| IPI00475159.1 | Q9CR86 | R.GNVVPSPLPT*R.R | 46 | VPSPLPTRRTRTF | Calcium-regulated heat stable protein 1 (Calcium-regulated heat-stable protein of 24 kDa) (CRHSP-24) | Carhsp1 | 148 |
| IPI00170092.2 | Q8K3I9 | R.GPSPS*S*PTPPPAAAPAEQAPR.A | 98 | ARGPSPSSPTPPP | Glucocorticoid-induced transcript 1 protein (Glucocorticoid-induced gene 18 protein) (Testhymin) (Thymocyte/spermatocyte selection protein 1) | Glcci1 Gig18 Tssn1 | 537 |
| IPI00170092.2 | Q8K3I9 | R.GPS*PSS*PTPPPAAAPAEQAPR.A | 96 | GGARGPSPSSPTP | Glucocorticoid-induced transcript 1 protein (Glucocorticoid-induced gene 18 protein) (Testhymin) (Thymocyte/spermatocyte selection protein 1) | Glcci1 Gig18 Tssn1 | 537 |
| IPI00170092.2 | Q8K3I9 | R.TSSLDT*ITGPYLTGQWPR.D | 166 | RTSSLDTITGPYL | Glucocorticoid-induced transcript 1 protein (Glucocorticoid-induced gene 18 protein) (Testhymin) (Thymocyte/spermatocyte selection protein 1) | Glcci1 Gig18 Tssn1 | 537 |
| IPI00132973.1 | Q9D0R8 | R.TET*PPPLASLNVSK.L | 75 | INDRTETPPPLAS | Protein LSM12 homolog | Lsm12 | 195 |
| IPI00229697.2 | Q8CI08 | R.SGAVQGAGLLGPGS*PAR.V | 48 | GLLGPGSPARVGV | SLAIN motif-containing protein 2 | Slain2 Kiaa1458 | 581 |
| IPI00229697.2 | Q8CI08 | K.SVS*PLVWCR.Q | 147 | PMQKSVSPLVWCR | SLAIN motif-containing protein 2 | Slain2 Kiaa1458 | 581 |
| IPI00317271.3 | | R.TDEIYIAGS*PLTPR.R |  |  |  |  |  |
| IPI00317271.3 | | R.TDEIYIAGSPLT*PR.R |  |  |  |  |  |
| IPI00317271.3 | | R.SITS*PTTLYDR.Y |  |  |  |  |  |
| IPI00317271.3 | | R.SNSTLPVPQPSS*APPT*PTR.L | |  |  |  |  |
| IPI00317271.3 | | K.YSQANSQTDT*PPLS*PYPFVR.T | |  |  |  |  |
| IPI00117305.2 | P70181 | R.DLLTEGQSFSS*LDEEALGSR.H | 448 | EGQSFSSLDEEAL | Phosphatidylinositol 4-phosphate 5-kinase type-1 beta (PIP5K1-beta) (PtdIns(4)P-5-kinase 1 beta) (EC 2.7.1.68) (Phosphatidylinositol 4-phosphate 5-kinase type I alpha) (PIP5KIalpha) (Phosphatidylinositol 4-phosphate 5-kinase type I beta) (PIP5KIbeta) | Pip5k1b Pip5k1a | 539 |
| IPI00117305.2 | P70181 | R.DLLTEGQSFS*SLDEEALGSR.H | 447 | TEGQSFSSLDEEA | Phosphatidylinositol 4-phosphate 5-kinase type-1 beta (PIP5K1-beta) (PtdIns(4)P-5-kinase 1 beta) (EC 2.7.1.68) (Phosphatidylinositol 4-phosphate 5-kinase type I alpha) (PIP5KIalpha) (Phosphatidylinositol 4-phosphate 5-kinase type I beta) (PIP5KIbeta) | Pip5k1b Pip5k1a | 539 |
| IPI00117305.2 | P70181 | R.DLLTEGQS*FSS*LDEEALGSR.H | 445 | LLTEGQSFSSLDE | Phosphatidylinositol 4-phosphate 5-kinase type-1 beta (PIP5K1-beta) (PtdIns(4)P-5-kinase 1 beta) (EC 2.7.1.68) (Phosphatidylinositol 4-phosphate 5-kinase type I alpha) (PIP5KIalpha) (Phosphatidylinositol 4-phosphate 5-kinase type I beta) (PIP5KIbeta) | Pip5k1b Pip5k1a | 539 |
| IPI00135443.2 | Q64511 | K.YTFDFS*EEEDDDAAAADDSNDLEELK.V | 1363 | KYTFDFSEEEDDD | DNA topoisomerase 2-beta (EC 5.99.1.3) (DNA topoisomerase II, beta isozyme) | Top2b | 1612 |
| IPI00135443.2 | Q64511 | K.SQDFGNLFSFPSY*SQK.S | 1440 | LFSFPSYSQKSED | DNA topoisomerase 2-beta (EC 5.99.1.3) (DNA topoisomerase II, beta isozyme) | Top2b | 1612 |
| IPI00135443.2 | Q64511 | K.SQDFGNLFSFPSYS*QK.S | 1441 | FSFPSYSQKSEDD | DNA topoisomerase 2-beta (EC 5.99.1.3) (DNA topoisomerase II, beta isozyme) | Top2b | 1612 |
| IPI00135443.2 | Q64511 | K.IVETINS*DSDS*EFGIPK.K | 1509 | IVETINSDSDSEF | DNA topoisomerase 2-beta (EC 5.99.1.3) (DNA topoisomerase II, beta isozyme) | Top2b | 1612 |
| IPI00135443.2 | Q64511 | K.IVETINSDS*DS*EFGIPK.K | 1511 | ETINSDSDSEFGI | DNA topoisomerase 2-beta (EC 5.99.1.3) (DNA topoisomerase II, beta isozyme) | Top2b | 1612 |
| IPI00229362.1 | P70255 | K.SPFNSPSPQDS*PR.L | 343 | SPSPQDSPRLSSF | Nuclear factor 1 C-type (NF1-C) (Nuclear factor 1/C) (CCAAT-box-binding transcription factor) (CTF) (Nuclear factor I/C) (NF-I/C) (NFI-C) (TGGCA-binding protein) | Nfic | 439 |
| IPI00775881.1 | Q6WVG3 | R.SPS*GGAAGPLLTPSQSLDGSR.R | 189 | LASRSPSGGAAGP | BTB/POZ domain-containing protein KCTD12 (Pfetin) (Predominantly fetal expressed T1 domain) | Kctd12 Pfet1 | 327 |
| IPI00117089.3 | Q61084 | R.HLS*VSSQNPGR.S | 166 | PRSRHLSVSSQNP | Mitogen-activated protein kinase kinase kinase 3 (EC 2.7.11.25) (MAPK/ERK kinase kinase 3) (MEK kinase 3) (MEKK 3) | Map3k3 Mekk3 | 626 |
| IPI00117089.3 | Q61084 | R.SLS*TNGENMGVAVQYLDPR.G | 316 | PSSRSLSTNGENM | Mitogen-activated protein kinase kinase kinase 3 (EC 2.7.11.25) (MAPK/ERK kinase kinase 3) (MEK kinase 3) (MEKK 3) | Map3k3 Mekk3 | 626 |
| IPI00117089.3 | Q61084 | K.S*PSAPINWR.R | 355 | RNVPTKSPSAPIN | Mitogen-activated protein kinase kinase kinase 3 (EC 2.7.11.25) (MAPK/ERK kinase kinase 3) (MEK kinase 3) (MEKK 3) | Map3k3 Mekk3 | 626 |
| IPI00172174.3 | | R.YGVYEDENYEVGS*DDEEIPFK.C | | |  |  |  |
| IPI00129918.1 | P97801 | R.GTGQSDDS*DIWDDTALIK.A | 28 | TGQSDDSDIWDDT | Survival motor neuron protein | Smn1 Smn | 288 |
| IPI00129918.1 | P97801 | R.GTGQS*DDS*DIWDDTALIK.A | 25 | RRGTGQSDDSDIW | Survival motor neuron protein | Smn1 Smn | 288 |
| IPI00659338.1 | | R.SSS*LSDLTHR.R |  |  |  |  |  |
| IPI00674306.3 | | R.GLASGSAET*LPANFR.V | |  |  |  |  |
| IPI00128196.1 | Q9Z2D1 | K.SSS*CESLGAQLPAAR.L | 6 | _MEKSSSCESLGA | Myotubularin-related protein 2 (Phosphatidylinositol-3,5-bisphosphate 3-phosphatase) (EC 3.1.3.95) (Phosphatidylinositol-3-phosphate phosphatase) (EC 3.1.3.64) | Mtmr2 | 643 |
| IPI00226379.4 | Q8C708 | R.QGS*LEPDWGLQPR.V | 195 | WRPRQGSLEPDWG | Transmembrane protein C16orf54 homolog | 0 | 225 |
| IPI00132175.1 | Q9CPY3 | K.SGS*DLPNSFSEIWPR.T | 34 | SKRKSGSDLPNSF | Sororin (Cell division cycle-associated protein 5) | Cdca5 | 264 |
| IPI00268247.4 | Q8R080 | K.VPTLS*PLTQQPQT*PEQR.G | 398 | PKVPTLSPLTQQP | G2 and S phase-expressed protein 1 (GTSE-1) (Protein B99) | Gtse1 B99 | 741 |
| IPI00268247.4 | Q8R080 | R.ALVS*PLCVPAR.R | 541 | MPRALVSPLCVPA | G2 and S phase-expressed protein 1 (GTSE-1) (Protein B99) | Gtse1 B99 | 741 |
| IPI00268247.4 | Q8R080 | K.LDQLTIT*PEAGGR.D | 656 | LDQLTITPEAGGR | G2 and S phase-expressed protein 1 (GTSE-1) (Protein B99) | Gtse1 B99 | 741 |
| IPI00652962.1 | Q8C080 | R.EFLCLDDPPGPFDS*LEESR.A | 222 | PPGPFDSLEESRA | Sorting nexin-16 | Snx16 | 344 |
| IPI00137712.1 | | R.TFS*PQKNESSQK.E |  |  |  |  |  |
| IPI00649980.1 | O08609 | R.GAPQLS*PES*PLLSGAR.G | 45 | RGAPQLSPESPLL | Max-like protein X (Max-like bHLHZip protein) (Protein BigMax) (Transcription factor-like protein 4) | Mlx Tcfl4 | 298 |
| IPI00134599.1 | P62908 | K.DEILPTT*PISEQK.G | 221 | DEILPTTPISEQK | 40S ribosomal protein S3 (EC 4.2.99.18) | Rps3 | 243 |
| IPI00758052.2 | Q9WUT3 | R.AYS*FCGTIEYMAPEVVNR.R | 218 | HDKRAYSFCGTIE | Ribosomal protein S6 kinase alpha-2 (S6K-alpha-2) (EC 2.7.11.1) (90 kDa ribosomal protein S6 kinase 2) (p90-RSK 2) (p90RSK2) (MAP kinase-activated protein kinase 1c) (MAPK-activated protein kinase 1c) (MAPKAP kinase 1c) (MAPKAPK-1c) (Protein-tyrosine kinase Mpk-9) (Ribosomal S6 kinase 3) (RSK-3) (pp90RSK3) | Rps6ka2 Mapkapk1c Rsk3 | 733 |
| IPI00758052.2 | Q9WUT3 | R.GFS*FVASS*LVQEPSQQDVPK.A | 377 | HLFRGFSFVASSL | Ribosomal protein S6 kinase alpha-2 (S6K-alpha-2) (EC 2.7.11.1) (90 kDa ribosomal protein S6 kinase 2) (p90-RSK 2) (p90RSK2) (MAP kinase-activated protein kinase 1c) (MAPK-activated protein kinase 1c) (MAPKAP kinase 1c) (MAPKAPK-1c) (Protein-tyrosine kinase Mpk-9) (Ribosomal S6 kinase 3) (RSK-3) (pp90RSK3) | Rps6ka2 Mapkapk1c Rsk3 | 733 |
| IPI00228590.2 | C0HKD9 | R.SLAALDALNT*DDENDEEEYEAWK.V | 267 | ALDALNTDDENDE | Microfibrillar-associated protein 1B (Spliceosome B complex protein MFAP1B) | Mfap1b | 439 |
| IPI00265987.3 | Q9WU62 | R.ASWGLQDSPGS*TDSPWQER.V | 248 | LQDSPGSTDSPWQ | Inner centromere protein | Incenp | 880 |
| IPI00265987.3 | Q9WU62 | R.AS*WGLQDS*PGSTDSPWQER.V | 239 | LKIARASWGLQDS | Inner centromere protein | Incenp | 880 |
| IPI00265987.3 | Q9WU62 | R.TSS*AVWNSPPLK.A | 862 | YHKRTSSAVWNSP | Inner centromere protein | Incenp | 880 |
| IPI00387580.1 | Q9QYB5 | R.VTQILQS*PAFR.E | 64 | VTQILQSPAFRED | Gamma-adducin (Adducin-like protein 70) | Add3 Addl | 706 |
| IPI00387580.1 | Q9QYB5 | K.SDVEIPATVTAFSFEDDSAPLS*PLK.F | 423 | DDSAPLSPLKFMA | Gamma-adducin (Adducin-like protein 70) | Add3 Addl | 706 |
| IPI00752452.1 | Q8BQS4 | K.ILQSQDFSLDS*SAEEEGLR.L | 299 | QDFSLDSSAEEEG | Protein FAM102B | Fam102b | 339 |
| IPI00762360.1 | Q6P542 | K.QLSVPAS*DEEDEVPAPIPR.G | 107 | QLSVPASDEEDEV | ATP-binding cassette sub-family F member 1 | Abcf1 | 837 |
| IPI00124064.3 | Q8VDZ4 | K.SIGSASPGPGQPPLS*SPTR.G | 693 | PGQPPLSSPTRGG | Palmitoyltransferase ZDHHC5 (EC 2.3.1.225) (Zinc finger DHHC domain-containing protein 5) (DHHC-5) | Zdhhc5 Kiaa1748 | 715 |
| IPI00652802.2 | Q8BXK8 | K.LDPPPS*PHANR.K | 521 | KLDPPPSPHANRK | Arf-GAP with GTPase, ANK repeat and PH domain-containing protein 1 (AGAP-1) (Centaurin-gamma-2) (Cnt-g2) | Agap1 Centg2 Kiaa1099 | 857 |
| IPI00652802.2 | Q8BXK8 | R.LT*SQSEAMALQSIR.N | 604 | KNKSRLTSQSEAM | Arf-GAP with GTPase, ANK repeat and PH domain-containing protein 1 (AGAP-1) (Centaurin-gamma-2) (Cnt-g2) | Agap1 Centg2 Kiaa1099 | 857 |
| IPI00460449.1 | Q6Y5D8 | R.MS*PFPLS*PAASIVDK.L | 710 | PPSPRMSPFPLSP | Rho GTPase-activating protein 10 (PH and SH3 domain-containing rhoGAP protein) (PS-GAP) (PSGAP) (Rho-type GTPase-activating protein 10) | Arhgap10 | 786 |
| IPI00458613.1 | Q6PIU9 | R.AS*DDLGEPDVFATAPFR.S | 115 | PINQRASDDLGEP | Uncharacterized protein FLJ45252 homolog | 0 | 354 |
| IPI00648095.1 | Q3TVI4 | R.TQS*PGGCSVEAVLAR.K | 75 | RGSRTQSPGGCSV | Protein HEXIM2 | Hexim2 | 313 |
| IPI00114333.2 | P18654 | K.AYS*FCGTVEYMAPEVVNR.R | 227 | HEKKAYSFCGTVE | Ribosomal protein S6 kinase alpha-3 (S6K-alpha-3) (EC 2.7.11.1) (90 kDa ribosomal protein S6 kinase 3) (p90-RSK 3) (p90RSK3) (MAP kinase-activated protein kinase 1b) (MAPK-activated protein kinase 1b) (MAPKAP kinase 1b) (MAPKAPK-1b) (Ribosomal S6 kinase 2) (RSK-2) (pp90RSK2) | Rps6ka3 Mapkapk1b Rps6ka-rs1 Rsk2 | 740 |
| IPI00114333.2 | P18654 | K.FS*LSGGYWNSVSDTAK.D | 639 | IGSGKFSLSGGYW | Ribosomal protein S6 kinase alpha-3 (S6K-alpha-3) (EC 2.7.11.1) (90 kDa ribosomal protein S6 kinase 3) (p90-RSK 3) (p90RSK3) (MAP kinase-activated protein kinase 1b) (MAPK-activated protein kinase 1b) (MAPKAP kinase 1b) (MAPKAPK-1b) (Ribosomal S6 kinase 2) (RSK-2) (pp90RSK2) | Rps6ka3 Mapkapk1b Rps6ka-rs1 Rsk2 | 740 |
| IPI00761357.2 | Q5F2E7 | K.DYEIENQNPLAS*PTNTLLGSAK.E | 626 | NQNPLASPTNTLL | Nuclear fragile X mental retardation-interacting protein 2 (82 kDa FMRP-interacting protein) (82-FIP) (FMRP-interacting protein 2) | Nufip2 Kiaa1321 | 692 |
| IPI00761357.2 | Q5F2E7 | R.NDS*WGSFDLR.A | 649 | GLERNDSWGSFDL | Nuclear fragile X mental retardation-interacting protein 2 (82 kDa FMRP-interacting protein) (82-FIP) (FMRP-interacting protein 2) | Nufip2 Kiaa1321 | 692 |
| IPI00128126.1 | Q91WG5 | K.TVFPFSYQES*PPR.S | 113 | PFSYQESPPRSPR | 5'-AMP-activated protein kinase subunit gamma-2 (AMPK gamma2) (AMPK subunit gamma-2) | Prkag2 | 566 |
| IPI00128126.1 | Q91WG5 | R.IYASSS*PPDTGQR.F | 196 | RIYASSSPPDTGQ | 5'-AMP-activated protein kinase subunit gamma-2 (AMPK gamma2) (AMPK subunit gamma-2) | Prkag2 | 566 |
| IPI00659860.2 | Q91WK0 | R.GSGDT*SSLIDPDTSLSELR.E | 114 | RRGSGDTSSLIDP | Leucine-rich repeat flightless-interacting protein 2 (LRR FLII-interacting protein 2) | Lrrfip2 | 415 |
| IPI00762427.1 | P70268 | R.LIPSAVATGTFS*PNAS*PGAEIR.H | 536 | VATGTFSPNASPG | Serine/threonine-protein kinase N1 (EC 2.7.11.13) (Protein kinase C-like 1) (Protein kinase C-like PKN) (Protein-kinase C-related kinase 1) (Serine-threonine protein kinase N) | Pkn1 Pkn Prk1 Prkcl1 | 946 |
| IPI00762427.1 | P70268 | R.TDVSNFDEEFTGEAPTLS*PPR.D | 920 | GEAPTLSPPRDAR | Serine/threonine-protein kinase N1 (EC 2.7.11.13) (Protein kinase C-like 1) (Protein kinase C-like PKN) (Protein-kinase C-related kinase 1) (Serine-threonine protein kinase N) | Pkn1 Pkn Prk1 Prkcl1 | 946 |
| IPI00649373.1 | Q68FF6 | K.SLS*SPTDNLELSAR.S | 370 | QQGKSLSSPTDNL | ARF GTPase-activating protein GIT1 (ARF GAP GIT1) (G protein-coupled receptor kinase-interactor 1) (GRK-interacting protein 1) | Git1 | 770 |
| IPI00649373.1 | Q68FF6 | R.SMDS*SDLSDGAVTLQEYLELK.K | 422 | RARSMDSSDLSDG | ARF GTPase-activating protein GIT1 (ARF GAP GIT1) (G protein-coupled receptor kinase-interactor 1) (GRK-interacting protein 1) | Git1 | 770 |
| IPI00225322.1 | Q9QYC0 | R.AAVVTS*PPPTTAPHK.E | 12 | RAAVVTSPPPTTA | Alpha-adducin (Erythrocyte adducin subunit alpha) | Add1 | 735 |
| IPI00225322.1 | Q9QYC0 | K.SPPDQSAVPNT*PPSTPVK.L | 610 | QSAVPNTPPSTPV | Alpha-adducin (Erythrocyte adducin subunit alpha) | Add1 | 735 |
| IPI00622487.2 | Q8BQR4 | R.ILDEDSWS*DGDQEPITVDQTWR.G | 149 | LDEDSWSDGDQEP | KAT8 regulatory NSL complex subunit 2 (NSL complex protein NSL2) (Non-specific lethal 2 homolog) | Kansl2 Nsl2 | 486 |
| IPI00346015.5 | | K.TFEISEPVIPLSQS*QQR.L | |  |  |  |  |
| IPI00785384.1 | | R.SSS*PVTELTARS*PVK.Q | |  |  |  |  |
| IPI00785384.1 | | R.SRT*PPSAPSQSR.M |  |  |  |  |  |
| IPI00785384.1 | | R.S*PVPSAFSDQSR.S |  |  |  |  |  |
| IPI00785384.1 | | R.SVAQTT*PVAGSQSLSSGTVAK.S | |  |  |  |  |
| IPI00785384.1 | | R.SLS*YSPVER.R |  |  |  |  |  |
| IPI00785384.1 | | R.RQPS*PQPSPR.D |  |  |  |  |  |
| IPI00785384.1 | | R.RQPS*PQPS*PR.D |  |  |  |  |  |
| IPI00762507.1 | Q8R0S2 | R.S*LSESYELSSDLQDK.Q | 104 | AIKRSRSLSESYE | IQ motif and SEC7 domain-containing protein 1 | Iqsec1 Kiaa0763 | 961 |
| IPI00762507.1 | Q8R0S2 | R.SLS*ESYELSSDLQDK.Q | 106 | KRSRSLSESYELS | IQ motif and SEC7 domain-containing protein 1 | Iqsec1 Kiaa0763 | 961 |
| IPI00762507.1 | Q8R0S2 | K.SLAES*IDDALNCR.S | 251 | VKSLAESIDDALN | IQ motif and SEC7 domain-containing protein 1 | Iqsec1 Kiaa0763 | 961 |
| IPI00223890.1 | Q8C0P0 | R.DY*SFEEPNTEDLFVLPK.C | 586 | DKSIRDYSFEEPN | Serine/threonine-protein kinase greatwall (GW) (GWL) (EC 2.7.11.1) (Microtubule-associated serine/threonine-protein kinase-like) (MAST-L) | Mastl Gw Gwl | 865 |
| IPI00752684.1 | D3Z6Q9 | R.ASGSGSCNAPGS*PEGSSQLCSPR.A | 430 | SCNAPGSPEGSSQ | Bridging integrator 2 | Bin2 | 489 |
| IPI00759847.1 | Q9QYG0 | R.SRTASLT*S*AASIDGSR.S | 334 | SRTASLTSAASID | Protein NDRG2 (N-myc downstream-regulated gene 2 protein) (Protein Ndr2) | Ndrg2 Kiaa1248 Ndr2 | 371 |
| IPI00759847.1 | Q9QYG0 | R.T*ASLTSAASIDGSR.S | 330 | RLSRSRTASLTSA | Protein NDRG2 (N-myc downstream-regulated gene 2 protein) (Protein Ndr2) | Ndrg2 Kiaa1248 Ndr2 | 371 |
| IPI00380392.1 | | R.LFQGYS*FVAPS*ILFK.R | |  |  |  |  |
| IPI00121829.1 | P05627 | K.NSDLLT*SPDVGLLK.L | 62 | KNSDLLTSPDVGL | Transcription factor AP-1 (AH119) (Activator protein 1) (AP1) (Proto-oncogene c-Jun) (V-jun avian sarcoma virus 17 oncogene homolog) (Jun A) | Jun | 334 |
| IPI00121829.1 | P05627 | K.NSDLLTS*PDVGLLK.L | 63 | NSDLLTSPDVGLL | Transcription factor AP-1 (AH119) (Activator protein 1) (AP1) (Proto-oncogene c-Jun) (V-jun avian sarcoma virus 17 oncogene homolog) (Jun A) | Jun | 334 |
| IPI00667941.1 | B2RXC2 | R.AALS*PGSVFSPGR.G | 42 | SGRAALSPGSVFS | Kinase (EC 2.7.-.-) | Itpkb mCG_6217 | 942 |
| IPI00667941.1 | B2RXC2 | R.ILS*PPGPEEAQR.K | 125 | VAARILSPPGPEE | Kinase (EC 2.7.-.-) | Itpkb mCG_6217 | 942 |
| IPI00667941.1 | B2RXC2 | K.EGVAPLLGPAS*PTR.L | 247 | PLLGPASPTRLGT | Kinase (EC 2.7.-.-) | Itpkb mCG_6217 | 942 |
| IPI00134747.3 | Q9D2E2 | R.SLQS*QPGTQTLAEAEDGPPTK.Q | 352 | RKRSLQSQPGTQT | Target of EGR1 protein 1 | Toe1 | 511 |
| IPI00755540.1 | | R.GCDS*PDPDTSYVLT*PHTEEK.Y | |  |  |  |  |
| IPI00224575.1 | P61979 | R.GSY*GDLGGPIITTQVTIPK.D | 380 | AGGRGSYGDLGGP | Heterogeneous nuclear ribonucleoprotein K (hnRNP K) | Hnrnpk Hnrpk | 463 |
| IPI00330763.3 | Q80XP8 | K.VS*SLSPEQEQGLWK.Q | 190 | SGHHKVSSLSPEQ | Protein FAM76B | Fam76b | 339 |
| IPI00330763.3 | Q80XP8 | K.VSS*LSPEQEQGLWK.Q | 191 | GHHKVSSLSPEQE | Protein FAM76B | Fam76b | 339 |
| IPI00320587.1 | Q01147 | K.ILNDLS*SDAPGVPR.I | 142 | KILNDLSSDAPGV | Cyclic AMP-responsive element-binding protein 1 (CREB-1) (cAMP-responsive element-binding protein 1) | Creb1 Creb-1 | 341 |
| IPI00119799.1 | O54918 | R.S*PLFIFVR.R | 73 | GPFATRSPLFIFV | Bcl-2-like protein 11 (Bcl2-L-11) (Bcl2-interacting mediator of cell death) | Bcl2l11 Bim | 196 |
| IPI00352179.2 | Q64701 | K.EAVT*TPVASATQSVSR.L | 384 | QEKEAVTTPVASA | Retinoblastoma-like protein 1 (107 kDa retinoblastoma-associated protein) (p107) (pRb1) | Rbl1 | 1063 |
| IPI00153375.1 | Q8R1G6 | R.FSS*LDLEEDSEVFK.M | 210 | SSPRFSSLDLEED | PDZ and LIM domain protein 2 (PDZ-LIM protein mystique) | Pdlim2 | 349 |
| IPI00153375.1 | Q8R1G6 | R.FS*SLDLEEDSEVFK.M | 209 | PSSPRFSSLDLEE | PDZ and LIM domain protein 2 (PDZ-LIM protein mystique) | Pdlim2 | 349 |
| IPI00471007.1 | E9Q0A3 | R.FPS*ISESLVNR.N | 232 | GTERFPSISESLV | Rho guanine nucleotide exchange factor (GEF) 11 (Fragment) | Arhgef11 | 1475 |
| IPI00471007.1 | E9Q0A3 | R.NSVLSDPGLDSPQT*SPVILAR.V | 254 | GLDSPQTSPVILA | Rho guanine nucleotide exchange factor (GEF) 11 (Fragment) | Arhgef11 | 1475 |
| IPI00471007.1 | E9Q0A3 | R.NSVLSDPGLDSPQT*S*PVILAR.V | 254 | GLDSPQTSPVILA | Rho guanine nucleotide exchange factor (GEF) 11 (Fragment) | Arhgef11 | 1475 |
| IPI00471007.1 | E9Q0A3 | R.LSTGSFPEDLLES*DSSR.S | 601 | PEDLLESDSSRSE | Rho guanine nucleotide exchange factor (GEF) 11 (Fragment) | Arhgef11 | 1475 |
| IPI00471007.1 | E9Q0A3 | R.LS*TGSFPEDLLESDSSR.S | 590 | PGTQRLSTGSFPE | Rho guanine nucleotide exchange factor (GEF) 11 (Fragment) | Arhgef11 | 1475 |
| IPI00471007.1 | E9Q0A3 | R.S*LENPTPPFTPK.M | 664 | SSLSTRSLENPTP | Rho guanine nucleotide exchange factor (GEF) 11 (Fragment) | Arhgef11 | 1475 |
| IPI00648562.1 | Q925J9 | K.DNPAQDFSTLYGSS*PLER.Q | 664 | STLYGSSPLERQN | Mediator of RNA polymerase II transcription subunit 1 (Mediator complex subunit 1) (Peroxisome proliferator-activated receptor-binding protein) (PBP) (PPAR-binding protein) (Thyroid hormone receptor-associated protein complex 220 kDa component) (Trap220) (Thyroid receptor-interacting protein 2) (TR-interacting protein 2) (TRIP-2) | Med1 Crsp210 Drip205 Pbp Pparbp Trap220 Trip2 | 1575 |
| IPI00648562.1 | Q925J9 | K.LPSTSDDCPPIGT*PVR.D | 805 | DCPPIGTPVRDSS | Mediator of RNA polymerase II transcription subunit 1 (Mediator complex subunit 1) (Peroxisome proliferator-activated receptor-binding protein) (PBP) (PPAR-binding protein) (Thyroid hormone receptor-associated protein complex 220 kDa component) (Trap220) (Thyroid receptor-interacting protein 2) (TR-interacting protein 2) (TRIP-2) | Med1 Crsp210 Drip205 Pbp Pparbp Trap220 Trip2 | 1575 |
| IPI00648562.1 | Q925J9 | R.S*QTPPGVAT*PPIPK.I | 1049 | PGSSGRSQTPPGV | Mediator of RNA polymerase II transcription subunit 1 (Mediator complex subunit 1) (Peroxisome proliferator-activated receptor-binding protein) (PBP) (PPAR-binding protein) (Thyroid hormone receptor-associated protein complex 220 kDa component) (Trap220) (Thyroid receptor-interacting protein 2) (TR-interacting protein 2) (TRIP-2) | Med1 Crsp210 Drip205 Pbp Pparbp Trap220 Trip2 | 1575 |
| IPI00648562.1 | Q925J9 | K.NYGSPLISGST*PK.H | 1442 | PLISGSTPKHERG | Mediator of RNA polymerase II transcription subunit 1 (Mediator complex subunit 1) (Peroxisome proliferator-activated receptor-binding protein) (PBP) (PPAR-binding protein) (Thyroid hormone receptor-associated protein complex 220 kDa component) (Trap220) (Thyroid receptor-interacting protein 2) (TR-interacting protein 2) (TRIP-2) | Med1 Crsp210 Drip205 Pbp Pparbp Trap220 Trip2 | 1575 |
| IPI00672213.2 | Q80TV8 | K.VSSSSGSPAFSSAAALPPGSYAS*LGR.I | 636 | PPGSYASLGRIRT | CLIP-associating protein 1 (Cytoplasmic linker-associated protein 1) | Clasp1 Kiaa0622 | 1535 |
| IPI00758171.1 | O54826 | K.SSSGSSVQS*PQDFLSFTDSDLR.S | 370 | SGSSVQSPQDFLS | Protein AF-10 | Mllt10 Af10 | 1068 |
| IPI00453800.4 | Q8K327 | K.GQESSS*DQEQVDVESIDFSK.E | 643 | KGQESSSDQEQVD | Chromosome alignment-maintaining phosphoprotein 1 (Zinc finger protein 828) | Champ1 D8Ertd457e Kiaa1802 Zfp828 Znf828 | 802 |
| IPI00321871.5 | P42128 | R.SLVSPIPS*PTGTISVPNS*CPASPR.G | 229 | LVSPIPSPTGTIS | Forkhead box protein K1 (Myocyte nuclear factor) (MNF) | Foxk1 Mnf | 719 |
| IPI00153986.2 | Q3THK3 | K.GTS*RPGTPS*AEAASTSSTLR.A | 385 | GSSKGTSRPGTPS | General transcription factor IIF subunit 1 (Transcription initiation factor IIF subunit alpha) (TFIIF-alpha) | Gtf2f1 | 508 |
| IPI00165832.1 | Q8VDY9 | K.SVNEILGLAESS*PK.E | 307 | LGLAESSPKEPKV | Caspase activity and apoptosis inhibitor 1 (Conserved anti-apoptotic protein) (CAAP) | Caap1 Caap | 356 |
| IPI00229455.1 | Q60591 | R.DAGLS*PEQPALALAGVAAS*PR.F | 136 | GRDAGLSPEQPAL | Nuclear factor of activated T-cells, cytoplasmic 2 (NF-ATc2) (NFATc2) (NFAT pre-existing subunit) (NF-ATp) (T-cell transcription factor NFAT1) | Nfatc2 Nfat1 Nfatp | 927 |
| IPI00656221.1 | O55201 | R.DVTNLTVGGFTPMS*PR.I | 664 | GGFTPMSPRISSP | Transcription elongation factor SPT5 (DRB sensitivity-inducing factor large subunit) (DSIF large subunit) | Supt5h Supt5 | 1082 |
| IPI00656221.1 | O55201 | R.TPAQSGAWDPNNPNT*PSR.A | 822 | DPNNPNTPSRAEE | Transcription elongation factor SPT5 (DRB sensitivity-inducing factor large subunit) (DSIF large subunit) | Supt5h Supt5 | 1082 |
| IPI00471423.1 | Q9DBR4 | K.NLS*PAAVINLTSEK.L | 123 | DPNKNLSPAAVIN | Amyloid-beta A4 precursor protein-binding family B member 2 | Apbb2 | 760 |
| IPI00471423.1 | Q9DBR4 | K.S*FLNYYADLETSAR.E | 160 | QPRRTKSFLNYYA | Amyloid-beta A4 precursor protein-binding family B member 2 | Apbb2 | 760 |
| IPI00752578.1 | Q60710 | R.TPPST*PPATANLSADDDFQNTDLR.T | 25 | PRTPPSTPPATAN | Deoxynucleoside triphosphate triphosphohydrolase SAMHD1 (dNTPase) (EC 3.1.5.-) (Interferon-gamma-inducible protein Mg11) (SAM domain and HD domain-containing protein 1) (mSAMHD1) | Samhd1 Mg11 | 658 |
| IPI00752578.1 | Q60710 | R.T*PPS*TPPATANLSADDDFQNTDLR.T | 21 | CDGSPRTPPSTPP | Deoxynucleoside triphosphate triphosphohydrolase SAMHD1 (dNTPase) (EC 3.1.5.-) (Interferon-gamma-inducible protein Mg11) (SAM domain and HD domain-containing protein 1) (mSAMHD1) | Samhd1 Mg11 | 658 |
| IPI00752578.1 | Q60710 | R.TPPS*T*PPATANLSADDDFQNTDLR.T | 24 | SPRTPPSTPPATA | Deoxynucleoside triphosphate triphosphohydrolase SAMHD1 (dNTPase) (EC 3.1.5.-) (Interferon-gamma-inducible protein Mg11) (SAM domain and HD domain-containing protein 1) (mSAMHD1) | Samhd1 Mg11 | 658 |
| IPI00403329.1 | Q9Z2Q2 | R.ATSPS*NNVDEVQIPEISLSK.R | 59 | PRATSPSNNVDEV | Lysine-rich nucleolar protein 1 (Testis-specific gene 118 protein) | Knop1 Tsg118 | 478 |
| IPI00653383.2 | Q60769 | K.SQEFVETGLCYDT*R.N | 161 | TGLCYDTRNWNDE | Tumor necrosis factor alpha-induced protein 3 (TNF alpha-induced protein 3) (EC 2.3.2.-) (EC 3.4.19.12) (Putative DNA-binding protein A20) (Zinc finger protein A20) | Tnfaip3 Tnfip3 | 775 |
| IPI00653383.2 | Q60769 | R.TGNVSPSGCLS*QAAR.T | 577 | SPSGCLSQAARTP | Tumor necrosis factor alpha-induced protein 3 (TNF alpha-induced protein 3) (EC 2.3.2.-) (EC 3.4.19.12) (Putative DNA-binding protein A20) (Zinc finger protein A20) | Tnfaip3 Tnfip3 | 775 |
| IPI00659186.1 | Q8BH57 | R.ASGDYDNDCT*NPITPLCTQPDQVIK.G | 343 | DYDNDCTNPITPL | WD repeat-containing protein 48 (USP1-associated factor 1) | Wdr48 Kiaa1449 Uaf1 | 676 |
| IPI00403761.1 | Q8R4Z4 | R.FSASS*LSASGPESGVTTDR.K | 173 | GRFSASSLSASGP | ETS translocation variant 3 (ETS domain transcriptional repressor PE1) (PE-1) (Mitogenic Ets transcriptional suppressor) | Etv3 Mets Pe1 | 513 |
| IPI00403761.1 | Q8R4Z4 | R.FSASSLSASGPES*GVTTDR.K | 181 | SASGPESGVTTDR | ETS translocation variant 3 (ETS domain transcriptional repressor PE1) (PE-1) (Mitogenic Ets transcriptional suppressor) | Etv3 Mets Pe1 | 513 |
| IPI00751399.1 | Q6P5G6 | R.SES*LIDASEDSQLEAAIR.A | 258 | KCARSESLIDASE | UBX domain-containing protein 7 | Ubxn7 Kiaa0794 Ubxd7 | 467 |
| IPI00751399.1 | Q6P5G6 | R.SES*LIDASEDS*QLEAAIR.A | 258 | KCARSESLIDASE | UBX domain-containing protein 7 | Ubxn7 Kiaa0794 Ubxd7 | 467 |
| IPI00654422.1 | P97855 | K.ST*SPAPADVAPAQEDLR.T | 230 | DDVQKSTSPAPAD | Ras GTPase-activating protein-binding protein 1 (G3BP-1) (EC 3.6.4.12) (EC 3.6.4.13) (ATP-dependent DNA helicase VIII) (GAP SH3 domain-binding protein 1) (HDH-VIII) | G3bp1 G3bp | 465 |
| IPI00658532.2 | Q8CHG7 | R.Y*SIPDLAVDVEQVIGLEK.V | 500 | IKKASRYSIPDLA | Rap guanine nucleotide exchange factor 2 (Cyclic nucleotide ras GEF) (CNrasGEF) (Neural RAP guanine nucleotide exchange protein) (nRap GEP) (PDZ domain-containing guanine nucleotide exchange factor 1) (PDZ-GEF1) (RA-GEF-1) (Ras/Rap1-associating GEF-1) | Rapgef2 Kiaa0313 Pdzgef1 | 1496 |
| IPI00658532.2 | Q8CHG7 | K.ILSLS*EEGSLER.H | 1090 | KKILSLSEEGSLE | Rap guanine nucleotide exchange factor 2 (Cyclic nucleotide ras GEF) (CNrasGEF) (Neural RAP guanine nucleotide exchange protein) (nRap GEP) (PDZ domain-containing guanine nucleotide exchange factor 1) (PDZ-GEF1) (RA-GEF-1) (Ras/Rap1-associating GEF-1) | Rapgef2 Kiaa0313 Pdzgef1 | 1496 |
| IPI00658532.2 | Q8CHG7 | R.ASWAS*STGYWGEDSEGDTGTIK.R | 1327 | SRASWASSTGYWG | Rap guanine nucleotide exchange factor 2 (Cyclic nucleotide ras GEF) (CNrasGEF) (Neural RAP guanine nucleotide exchange protein) (nRap GEP) (PDZ domain-containing guanine nucleotide exchange factor 1) (PDZ-GEF1) (RA-GEF-1) (Ras/Rap1-associating GEF-1) | Rapgef2 Kiaa0313 Pdzgef1 | 1496 |
| IPI00658532.2 | Q8CHG7 | R.AS*WASSTGYWGEDSEGDTGTIK.R | 1324 | QAQSRASWASSTG | Rap guanine nucleotide exchange factor 2 (Cyclic nucleotide ras GEF) (CNrasGEF) (Neural RAP guanine nucleotide exchange protein) (nRap GEP) (PDZ domain-containing guanine nucleotide exchange factor 1) (PDZ-GEF1) (RA-GEF-1) (Ras/Rap1-associating GEF-1) | Rapgef2 Kiaa0313 Pdzgef1 | 1496 |
| IPI00658532.2 | Q8CHG7 | R.ASWASSTGYWGEDS*EGDTGTIK.R | 1336 | GYWGEDSEGDTGT | Rap guanine nucleotide exchange factor 2 (Cyclic nucleotide ras GEF) (CNrasGEF) (Neural RAP guanine nucleotide exchange protein) (nRap GEP) (PDZ domain-containing guanine nucleotide exchange factor 1) (PDZ-GEF1) (RA-GEF-1) (Ras/Rap1-associating GEF-1) | Rapgef2 Kiaa0313 Pdzgef1 | 1496 |
| IPI00229599.1 | Q8CH77 | K.GQLTNIVS*PTAATTPR.I | 998 | QLTNIVSPTAATT | Neuron navigator 1 (Pore membrane and/or filament-interacting-like protein 3) | Nav1 Kiaa1151 Pomfil3 | 1875 |
| IPI00229599.1 | Q8CH77 | K.GQLTNIVSPT*AATTPR.I | 1000 | TNIVSPTAATTPR | Neuron navigator 1 (Pore membrane and/or filament-interacting-like protein 3) | Nav1 Kiaa1151 Pomfil3 | 1875 |
| IPI00229599.1 | Q8CH77 | K.SASSYSDIEEIATPDSSAPS*SPK.L | 1250 | PDSSAPSSPKLQH | Neuron navigator 1 (Pore membrane and/or filament-interacting-like protein 3) | Nav1 Kiaa1151 Pomfil3 | 1875 |
| IPI00625592.1 | P08775 | K.YTPTS*PSYSPSSPEYTPAS*PK.Y | 1843 | PKYTPTSPSYSPS | DNA-directed RNA polymerase II subunit RPB1 (RNA polymerase II subunit B1) (EC 2.7.7.6) (DNA-directed RNA polymerase II subunit A) (DNA-directed RNA polymerase III largest subunit) | Polr2a Rpii215 Rpo2-1 | 1970 |
| IPI00625592.1 | P08775 | K.YTPTSPSYS*PSSPEY*TPASPK.Y | 1847 | PTSPSYSPSSPEY | DNA-directed RNA polymerase II subunit RPB1 (RNA polymerase II subunit B1) (EC 2.7.7.6) (DNA-directed RNA polymerase II subunit A) (DNA-directed RNA polymerase III largest subunit) | Polr2a Rpii215 Rpo2-1 | 1970 |
| IPI00625592.1 | P08775 | K.YSPTSPTYSPTSPVY*TPTSPK.Y | 1902 | SPTSPVYTPTSPK | DNA-directed RNA polymerase II subunit RPB1 (RNA polymerase II subunit B1) (EC 2.7.7.6) (DNA-directed RNA polymerase II subunit A) (DNA-directed RNA polymerase III largest subunit) | Polr2a Rpii215 Rpo2-1 | 1970 |
| IPI00625592.1 | P08775 | K.YSPTSPTY*SPTSPVYTPTSPK.Y | 1895 | SPTSPTYSPTSPV | DNA-directed RNA polymerase II subunit RPB1 (RNA polymerase II subunit B1) (EC 2.7.7.6) (DNA-directed RNA polymerase II subunit A) (DNA-directed RNA polymerase III largest subunit) | Polr2a Rpii215 Rpo2-1 | 1970 |
| IPI00625592.1 | P08775 | K.YSPTSPTYSPTS*PVYTPTSPK.Y | 1899 | PTYSPTSPVYTPT | DNA-directed RNA polymerase II subunit RPB1 (RNA polymerase II subunit B1) (EC 2.7.7.6) (DNA-directed RNA polymerase II subunit A) (DNA-directed RNA polymerase III largest subunit) | Polr2a Rpii215 Rpo2-1 | 1970 |
| IPI00625592.1 | P08775 | K.YSPTSPTYSPTS*PVYTPTS*PK.Y | 1899 | PTYSPTSPVYTPT | DNA-directed RNA polymerase II subunit RPB1 (RNA polymerase II subunit B1) (EC 2.7.7.6) (DNA-directed RNA polymerase II subunit A) (DNA-directed RNA polymerase III largest subunit) | Polr2a Rpii215 Rpo2-1 | 1970 |
| IPI00625592.1 | P08775 | K.YSPT*SPTYSPTSPVYTPT*SPK.Y | 1891 | TPKYSPTSPTYSP | DNA-directed RNA polymerase II subunit RPB1 (RNA polymerase II subunit B1) (EC 2.7.7.6) (DNA-directed RNA polymerase II subunit A) (DNA-directed RNA polymerase III largest subunit) | Polr2a Rpii215 Rpo2-1 | 1970 |
| IPI00625592.1 | P08775 | K.YSPTS*PTYSPTSPVYTPTS*PK.Y | 1892 | PKYSPTSPTYSPT | DNA-directed RNA polymerase II subunit RPB1 (RNA polymerase II subunit B1) (EC 2.7.7.6) (DNA-directed RNA polymerase II subunit A) (DNA-directed RNA polymerase III largest subunit) | Polr2a Rpii215 Rpo2-1 | 1970 |
| IPI00625592.1 | P08775 | K.YSPT*SPTYSPTSPVYTPTS*PK.Y | 1891 | TPKYSPTSPTYSP | DNA-directed RNA polymerase II subunit RPB1 (RNA polymerase II subunit B1) (EC 2.7.7.6) (DNA-directed RNA polymerase II subunit A) (DNA-directed RNA polymerase III largest subunit) | Polr2a Rpii215 Rpo2-1 | 1970 |
| IPI00625592.1 | P08775 | K.YSPTSPTYS*PTSPK.Y | 1917 | PTSPTYSPTSPKY | DNA-directed RNA polymerase II subunit RPB1 (RNA polymerase II subunit B1) (EC 2.7.7.6) (DNA-directed RNA polymerase II subunit A) (DNA-directed RNA polymerase III largest subunit) | Polr2a Rpii215 Rpo2-1 | 1970 |
| IPI00625592.1 | P08775 | K.YSPTSPTYSPTS*PK.Y | 1920 | PTYSPTSPKYSPT | DNA-directed RNA polymerase II subunit RPB1 (RNA polymerase II subunit B1) (EC 2.7.7.6) (DNA-directed RNA polymerase II subunit A) (DNA-directed RNA polymerase III largest subunit) | Polr2a Rpii215 Rpo2-1 | 1970 |
| IPI00625592.1 | P08775 | K.YSPTSPT*YSPTSPK.Y | 1915 | YSPTSPTYSPTSP | DNA-directed RNA polymerase II subunit RPB1 (RNA polymerase II subunit B1) (EC 2.7.7.6) (DNA-directed RNA polymerase II subunit A) (DNA-directed RNA polymerase III largest subunit) | Polr2a Rpii215 Rpo2-1 | 1970 |
| IPI00468516.3 | Q6PGL7 | R.GGLFS*NGQGLFDDEDESDLFK.E | 353 | SRGGLFSNGQGLF | WASH complex subunit 2 | Washc2 D6Wsu116e Fam21 Kiaa0592 | 1334 |
| IPI00468516.3 | Q6PGL7 | K.EGLLPASDQEAGGPSDIFSSSS*PLDK.G | 783 | DIFSSSSPLDKGA | WASH complex subunit 2 | Washc2 D6Wsu116e Fam21 Kiaa0592 | 1334 |
| IPI00648998.3 | | K.AYS*FCGTVEYMAPEVVNR.Q | |  |  |  |  |
| IPI00648998.3 | | R.KLPS*TTL.- |  |  |  |  |  |
| IPI00671412.2 | Q69ZZ6 | K.FGS*ADNIPNLK.D | 378 | IRNKFGSADNIPN | Transmembrane and coiled-coil domains protein 1 | Tmcc1 Kiaa0779 | 649 |
| IPI00380130.2 | P25444 | K.TYSYLT*PDLWK.E | 252 | KTYSYLTPDLWKE | 40S ribosomal protein S2 (40S ribosomal protein S4) (Protein LLRep3) | Rps2 Llrep3 Rps4 | 293 |
| IPI00626851.1 | Q9R0G7 | K.NANCTS*DFEEYFAK.R | 142 | KNANCTSDFEEYF | Zinc finger E-box-binding homeobox 2 (Smad-interacting protein 1) (Zinc finger homeobox protein 1b) | Zeb2 Sip1 Zfhx1b Zfx1b | 1215 |
| IPI00626851.1 | Q9R0G7 | K.GLTS*PINPYK.D | 647 | SEKGLTSPINPYK | Zinc finger E-box-binding homeobox 2 (Smad-interacting protein 1) (Zinc finger homeobox protein 1b) | Zeb2 Sip1 Zfhx1b Zfx1b | 1215 |
| IPI00330102.4 | Q68FD7 | R.WS*VEESFNLSDESCGPNPGIVR.K | 306 | GVFPRWSVEESFN | Folliculin-interacting protein 1 | Fnip1 Kiaa1961 | 1165 |
| IPI00330102.4 | Q68FD7 | K.VTFLIGDSMS*PDSDTELR.S | 760 | LIGDSMSPDSDTE | Folliculin-interacting protein 1 | Fnip1 Kiaa1961 | 1165 |
| IPI00118101.1 | Q99N57 | K.DAVFDGSSCIS*PTIVQQFGYQR.R | 29 | DGSSCISPTIVQQ | RAF proto-oncogene serine/threonine-protein kinase (EC 2.7.11.1) (Proto-oncogene c-RAF) (cRaf) (Raf-1) | Raf1 Craf | 648 |
| IPI00752541.1 | Q8BGD9 | K.SPPYT*AFLGNLPYDVTEDSIK.D | 97 | PKSPPYTAFLGNL | Eukaryotic translation initiation factor 4B (eIF-4B) | Eif4b | 611 |
| IPI00128615.1 | Q8VHR5 | R.LQQQAALS*PTTAPAVSSVSK.Q | 487 | QQQAALSPTTAPA | Transcriptional repressor p66-beta (GATA zinc finger domain-containing protein 2B) (p66/p68) | Gatad2b | 594 |
| IPI00224563.1 | Q8BS90 | R.GS*LEGQFSSSPIQNS*VKK.Y | 223 | PCSARGSLEGQFS | Protein aurora borealis | Bora | 525 |
| IPI00115094.1 | O55028 | R.STS*ATDTHHVELAR.E | 33 | LRARSTSATDTHH | [3-methyl-2-oxobutanoate dehydrogenase [lipoamide]] kinase, mitochondrial (EC 2.7.11.4) (Branched-chain alpha-ketoacid dehydrogenase kinase) (BCKD-kinase) (BCKDHKIN) | Bckdk | 412 |
| IPI00480235.1 | Q8C0E3 | R.SGALAS*PTDPFQSR.L | 591 | RSGALASPTDPFQ | E3 ubiquitin-protein ligase TRIM47 (EC 2.3.2.27) (Tripartite motif-containing protein 47) | Trim47 | 641 |
| IPI00311851.5 | Q8VDV3 | K.TLVITSTPAS*PNR.E | 169 | ITSTPASPNRELH | Guanine nucleotide exchange factor for Rab-3A (Rab-3A-interacting-like protein 1) (Rab3A-interacting-like protein 1) (Rabin3-like 1) | Rab3il1 | 383 |
| IPI00649424.2 | P24788 | K.AY*TPVVVTLWYR.A | 583 | GSPLKAYTPVVVT | Cyclin-dependent kinase 11B (Cell division cycle 2-like protein kinase 1) (Cell division protein kinase 11) (Cyclin-dependent kinase 11) (EC 2.7.11.22) (Galactosyltransferase-associated protein kinase p58/GTA) (PITSLRE serine/threonine-protein kinase CDC2L1) | Cdk11b Cdc2l1 Cdk11 | 784 |
| IPI00410959.1 | Q9DC28 | R.GAPVNVSS*SDLTGR.Q | 383 | APVNVSSSDLTGR | Casein kinase I isoform delta (CKI-delta) (CKId) (EC 2.7.11.1) (Tau-protein kinase CSNK1D) (EC 2.7.11.26) | Csnk1d Hckid | 415 |
| IPI00410959.1 | Q9DC28 | R.GAPVNVSSS*DLTGR.Q | 384 | PVNVSSSDLTGRQ | Casein kinase I isoform delta (CKI-delta) (CKId) (EC 2.7.11.1) (Tau-protein kinase CSNK1D) (EC 2.7.11.26) | Csnk1d Hckid | 415 |
| IPI00410953.1 | Q8BGR5 | R.S*WTALLSASS*PGGR.T | 335 | LVSSTRSWTALLS | Protein FAM53B | Fam53b | 422 |
| IPI00758043.1 | Q8BWA4 | R.FIQELSGSS*PK.R | 120 | QELSGSSPKRRRA | Uncharacterized protein | Tfap4 Tcfap4 | 334 |
| IPI00465706.2 | Q8BIE6 | R.SLS*EIAIDLTETGTLK.T | 320 | HAARSLSEIAIDL | FERM domain-containing protein 4A | Frmd4a Frmd4 Kiaa1294 | 1020 |
| IPI00465706.2 | Q8BIE6 | K.LLGSENDTGS*PDFYTPR.T | 712 | SENDTGSPDFYTP | FERM domain-containing protein 4A | Frmd4a Frmd4 Kiaa1294 | 1020 |
| IPI00227351.1 | Q80TJ7 | K.DAEYIYPSLES*DDDDPALK.S | 820 | IYPSLESDDDDPA | Histone lysine demethylase PHF8 (EC 1.14.11.27) (PHD finger protein 8) | Phf8 Kiaa1111 | 1023 |
| IPI00227351.1 | Q80TJ7 | R.VAS*IETGLAAAAAK.L | 867 | EGTRVASIETGLA | Histone lysine demethylase PHF8 (EC 1.14.11.27) (PHD finger protein 8) | Phf8 Kiaa1111 | 1023 |
| IPI00223371.1 | Q8VH51 | R.TDASSASS*FLDSDELER.T | 337 | DASSASSFLDSDE | RNA-binding protein 39 (Coactivator of activating protein 1 and estrogen receptors) (Coactivator of AP-1 and ERs) (RNA-binding motif protein 39) (RNA-binding region-containing protein 2) (Transcription coactivator CAPER) | Rbm39 Caper Rnpc2 | 530 |
| IPI00458001.1 | Q7TQH0 | K.GPPQS*PVFEGVYNNSR.M | 109 | GKGPPQSPVFEGV | Ataxin-2-like protein | Atxn2l A2lp | 1049 |
| IPI00458001.1 | Q7TQH0 | K.LQPSSS*PETGLDPFPSR.I | 562 | KLQPSSSPETGLD | Ataxin-2-like protein | Atxn2l A2lp | 1049 |
| IPI00420481.5 | A2APB8 | K.LQQGFVT*PLK.A | 72 | LQQGFVTPLKAVD | Targeting protein for Xklp2 | Tpx2 | 745 |
| IPI00420481.5 | A2APB8 | K.SSELPLTVPVS*PK.F | 737 | PLTVPVSPKFSTR | Targeting protein for Xklp2 | Tpx2 | 745 |
| IPI00453808.3 | Q6ZPL9 | K.DEGS*DIDDEDMEELLNDTR.L | 544 | RKKDEGSDIDDED | ATP-dependent RNA helicase DDX55 (EC 3.6.4.13) (DEAD box protein 55) | Ddx55 Kiaa1595 | 600 |
| IPI00757751.1 | Q61083 | R.GSDIDNPTLTVTDIS*PPSR.S | 344 | LTVTDISPPSRSP | Mitogen-activated protein kinase kinase kinase 2 (EC 2.7.11.25) (MAPK/ERK kinase kinase 2) (MEK kinase 2) (MEKK 2) | Map3k2 Mekk2 | 619 |
| IPI00120089.1 | Q9DBS5 | R.AAS*LNYLNQPNAAPLQVSR.G | 590 | SMKRAASLNYLNQ | Kinesin light chain 4 (KLC 4) (Kinesin-like protein 8) | Klc4 Knsl8 | 619 |
| IPI00457474.1 | Q811D8 | K.TNEDALS*FNDALMVEHAR.R | 304 | TNEDALSFNDALM | Butyrophilin-like | LOC100045026 | 582 |
| IPI00123967.1 | Q62073 | R.SIQDLT*VTGTEPGQVSSR.S | 417 | RSIQDLTVTGTEP | Mitogen-activated protein kinase kinase kinase 7 (EC 2.7.11.25) (Transforming growth factor-beta-activated kinase 1) (TGF-beta-activated kinase 1) | Map3k7 Tak1 | 579 |
| IPI00123967.1 | Q62073 | R.S*IQDLTVTGTEPGQVSSR.S | 412 | GQPRRRSIQDLTV | Mitogen-activated protein kinase kinase kinase 7 (EC 2.7.11.25) (Transforming growth factor-beta-activated kinase 1) (TGF-beta-activated kinase 1) | Map3k7 Tak1 | 579 |
| IPI00554927.1 | | R.GDS*LAYGLR.S |  |  |  |  |  |
| IPI00661969.1 | | R.AATS*AVTAYEPLDSLDR.R | |  |  |  |  |
| IPI00624206.1 | Q8CFA1 | K.DLLLSEIPNSTSSVCS*R.K | 439 | STSSVCSRKTSMG | Interleukin-1 receptor-associated kinase-like 2 (IRAK-2) (mu-IRAK-2) | Irak2 | 622 |
| IPI00139382.1 | P58801 | R.ASSCS*LAVISPFLVEK.G | 414 | RRASSCSLAVISP | Receptor-interacting serine/threonine-protein kinase 2 (EC 2.7.11.1) (Tyrosine-protein kinase RIPK2) (EC 2.7.10.2) | Ripk2 | 539 |
| IPI00659672.1 | | K.S*PGEQLVCLPPVEAFPNDPR.V | |  |  |  |  |
| IPI00659672.1 | | R.VVS*ISSSDFSAK.E |  |  |  |  |  |
| IPI00120516.2 | Q9ES52 | R.GEGPPTPPSQPPLS*PK.K | 972 | PSQPPLSPKKFSS | Phosphatidylinositol 3,4,5-trisphosphate 5-phosphatase 1 (EC 3.1.3.86) (Inositol polyphosphate-5-phosphatase of 145 kDa) (SIP-145) (SH2 domain-containing inositol 5'-phosphatase 1) (SH2 domain-containing inositol phosphatase 1) (SHIP-1) (p150Ship) | Inpp5d 7a33 Ship Ship1 | 1191 |
| IPI00119632.3 | Q9DBE9 | K.DGFS*GIEDDADEALEISQAQLLYK.S | 532 | FSKDGFSGIEDDA | pre-rRNA processing protein FTSJ3 (EC 2.1.1.-) (2'-O-ribose RNA methyltransferase SPB1 homolog) (Protein ftsJ homolog 3) (Putative rRNA methyltransferase 3) | Ftsj3 | 838 |
| IPI00659577.1 | Q6ZPR6 | R.SDSSGGY*TLSDVIQSPPSAGLLK.S | 997 | SDSSGGYTLSDVI | Inhibitor of Bruton tyrosine kinase (IBtk) | Ibtk Kiaa1417 | 1352 |
| IPI00127071.3 | Q91VN6 | R.SEDEDEDDEDY*VPYVPLR.Q | 33 | DEDDEDYVPYVPL | Probable ATP-dependent RNA helicase DDX41 (EC 3.6.4.13) (DEAD box protein 41) | Ddx41 | 622 |
| IPI00320527.3 | Q8K1H7 | R.FAEGVASLSDY*ECSR.Q | 33 | VASLSDYECSRQS | T-complex protein 11-like protein 2 | Tcp11l2 | 517 |
| IPI00124171.1 | P97360 | R.ISY*TPPES*PVASHR.S | 17 | KQERISYTPPESP | Transcription factor ETV6 (ETS translocation variant 6) (ETS-related protein Tel1) (Tel) | Etv6 Tel Tel1 | 485 |
| IPI00323397.1 | P23804 | R.SLS*FDPSLGLCELR.E | 185 | KRRRSLSFDPSLG | E3 ubiquitin-protein ligase Mdm2 (EC 2.3.2.27) (Double minute 2 protein) (Oncoprotein Mdm2) (RING-type E3 ubiquitin transferase Mdm2) (p53-binding protein Mdm2) | Mdm2 | 489 |
| IPI00323397.1 | P23804 | R.S*LSFDPSLGLCELR.E | 183 | HRKRRRSLSFDPS | E3 ubiquitin-protein ligase Mdm2 (EC 2.3.2.27) (Double minute 2 protein) (Oncoprotein Mdm2) (RING-type E3 ubiquitin transferase Mdm2) (p53-binding protein Mdm2) | Mdm2 | 489 |
| IPI00308559.1 | Q8BIZ6 | R.S*GDALTTVVVK.Q | 18 | GRRRHRSGDALTT | Smad nuclear-interacting protein 1 | Snip1 | 383 |
| IPI00395177.1 | Q5SSL4 | R.SQGGGDSVSPT*PPEGLAPGVEAGK.G | 74 | GDSVSPTPPEGLA | Active breakpoint cluster region-related protein | Abr | 859 |
| IPI00278134.1 | Q8R1S4 | K.GSDYSWSYQT*PPS*SPSTTMSR.K | 262 | YSWSYQTPPSSPS | Metastasis suppressor protein 1 (Missing in metastasis protein) | Mtss1 Mim | 759 |
| IPI00230639.1 | Q99PG2 | K.IALNLEGCALS*PTSQEPR.E | 403 | LEGCALSPTSQEP | Opioid growth factor receptor (OGFr) (Zeta-type opioid receptor) | Ogfr | 633 |
| IPI00420378.2 | Q8K4B2 | K.TPFECSQS*EVTFLGLDR.N | 525 | PFECSQSEVTFLG | Interleukin-1 receptor-associated kinase 3 (IRAK-3) (EC 2.7.11.1) (IL-1 receptor-associated kinase M) (IRAK-M) | Irak3 | 609 |
| IPI00420378.2 | Q8K4B2 | K.TPFECS*QSEVTFLGLDR.N | 523 | KTPFECSQSEVTF | Interleukin-1 receptor-associated kinase 3 (IRAK-3) (EC 2.7.11.1) (IL-1 receptor-associated kinase M) (IRAK-M) | Irak3 | 609 |
| IPI00136863.1 | P31649 | K.NQPEPTAPATPMTS*LLR.L | 591 | PATPMTSLLRLTE | Sodium- and chloride-dependent GABA transporter 2 (GAT-2) (Sodium- and chloride-dependent GABA transporter 3) (GAT-3) (Solute carrier family 6 member 13) | Slc6a13 Gabt2 Gabt3 Gat-3 Gat2 Gat3 | 602 |
| IPI00222813.1 | Q8BLH7 | R.NAWNPSGEGT*SPGETYR.R | 563 | NPSGEGTSPGETY | HIRA-interacting protein 3 | Hirip3 | 601 |
| IPI00230677.1 | Q9JIX8 | K.SQSPS*PPPLPEDLEK.A | 391 | EKSQSPSPPPLPE | Apoptotic chromatin condensation inducer in the nucleus (Acinus) | Acin1 Acinus | 1338 |
| IPI00230677.1 | Q9JIX8 | K.SQS*PSPPPLPEDLEK.A | 389 | LEEKSQSPSPPPL | Apoptotic chromatin condensation inducer in the nucleus (Acinus) | Acin1 Acinus | 1338 |
| IPI00230677.1 | Q9JIX8 | R.SLS*PLSGTTDTK.A | 479 | QLLRSLSPLSGTT | Apoptotic chromatin condensation inducer in the nucleus (Acinus) | Acin1 Acinus | 1338 |
| IPI00230677.1 | Q9JIX8 | R.TAQVPS*PPR.G | 1003 | RTAQVPSPPRGKI | Apoptotic chromatin condensation inducer in the nucleus (Acinus) | Acin1 Acinus | 1338 |
| IPI00420949.2 | | K.SQIDVALS*QDSTYQGER.A | |  |  |  |  |
| IPI00310764.6 | Q60875 | R.LES*FESLR.G | 646 | GLFRLESFESLRG | Rho guanine nucleotide exchange factor 2 (Guanine nucleotide exchange factor H1) (GEF-H1) (LBC'S first cousin) (Lymphoid blast crisis-like 1) (Oncogene LFC) (Rhobin) | Arhgef2 Kiaa0651 Lbcl1 Lfc | 985 |
| IPI00310764.6 | Q60875 | R.S*LPAGDALYLSFNPPQPSR.G | 885 | LDPRRRSLPAGDA | Rho guanine nucleotide exchange factor 2 (Guanine nucleotide exchange factor H1) (GEF-H1) (LBC'S first cousin) (Lymphoid blast crisis-like 1) (Oncogene LFC) (Rhobin) | Arhgef2 Kiaa0651 Lbcl1 Lfc | 985 |
| IPI00310764.6 | Q60875 | R.SLPAGDALY*LSFNPPQPSR.G | 893 | PAGDALYLSFNPP | Rho guanine nucleotide exchange factor 2 (Guanine nucleotide exchange factor H1) (GEF-H1) (LBC'S first cousin) (Lymphoid blast crisis-like 1) (Oncogene LFC) (Rhobin) | Arhgef2 Kiaa0651 Lbcl1 Lfc | 985 |
| IPI00137361.1 | P70218 | R.SDPS*LWNPAAPEPGQPPLVPPR.K | 455 | TARSDPSLWNPAA | Mitogen-activated protein kinase kinase kinase kinase 1 (EC 2.7.11.1) (Hematopoietic progenitor kinase) (HPK) (MAPK/ERK kinase kinase kinase 1) (MEK kinase kinase 1) (MEKKK 1) | Map4k1 Hpk1 | 827 |
| IPI00469000.4 | Q8C145 | K.ES*ASSSEVTSAVYNAVSEGTR.F | 188 | RRNIKESASSSEV | Zinc transporter ZIP6 (Endoplasmic reticulum membrane-linked protein) (Ermelin) (Solute carrier family 39 member 6) (Zrt- and Irt-like protein 6) (ZIP-6) | Slc39a6 Zip6 | 765 |
| IPI00153284.1 | Q8R149 | R.YLS*GTDAGLEGGPEAGR.K | 18 | YLKRYLSGTDAGL | BUD13 homolog | Bud13 | 637 |
| IPI00750302.1 | P70459 | R.GDVGPGESGGPLT*PR.R | 529 | ESGGPLTPRRVSS | ETS domain-containing transcription factor ERF | Erf | 551 |
| IPI00649804.2 | Q9DBC7 | R.EDEIS*PPPPNPVVK.G | 83 | SREDEISPPPPNP | cAMP-dependent protein kinase type I-alpha regulatory subunit [Cleaved into: cAMP-dependent protein kinase type I-alpha regulatory subunit, N-terminally processed] | Prkar1a | 381 |
| IPI00230620.2 | Q61127 | K.LS*PLPGGPGAGDPR.I | 171 | ELGEKLSPLPGGP | NGFI-A-binding protein 2 (EGR-1-binding protein 2) | Nab2 | 525 |
| IPI00464302.7 | Q3UE31 | R.VTSFSTPPT*PER.N | 293 | SFSTPPTPERNNR | Uncharacterized protein KIAA0930 homolog | 0 | 404 |
| IPI00471230.1 | Q9JIY0 | R.CAS*LEEILSQR.D | 270 | EKSRCASLEEILS | Pleckstrin homology domain-containing family O member 1 (PH domain-containing family O member 1) | Plekho1 | 408 |
| IPI00515528.2 | E9Q784 | R.T*PS*PPPPILEDIILGK.K | 263 | KKKGPRTPSPPPP | Zinc finger CCCH domain-containing protein 13 | Zc3h13 | 1729 |
| IPI00515528.2 | E9Q784 | K.ENTAFSDWS*DEDVPDR.T | 1144 | TAFSDWSDEDVPD | Zinc finger CCCH domain-containing protein 13 | Zc3h13 | 1729 |
| IPI00515528.2 | E9Q784 | K.ENTAFS*DWS*DEDVPDR.T | 1141 | KENTAFSDWSDED | Zinc finger CCCH domain-containing protein 13 | Zc3h13 | 1729 |
| IPI00228233.1 | Q571I4 | R.TIYLSS*PDSAVGVQWPR.G | 277 | RTIYLSSPDSAVG | Inactive tyrosine-protein kinase PRAG1 (Notch activation complex kinase) (PEAK1-related kinase-activating pseudokinase 1) (Sugen kinase 223) (Tyrosine-protein kinase SgK223) | Prag1 D8Ertd82e Nack Sgk223 | 1373 |
| IPI00228233.1 | Q571I4 | K.VSQSSAESLS*PSFR.G | 526 | SSAESLSPSFRGA | Inactive tyrosine-protein kinase PRAG1 (Notch activation complex kinase) (PEAK1-related kinase-activating pseudokinase 1) (Sugen kinase 223) (Tyrosine-protein kinase SgK223) | Prag1 D8Ertd82e Nack Sgk223 | 1373 |
| IPI00377615.1 | A0PJJ1 | R.SFS*LDEPPLFIPDNIATVK.K | 660 | RSSRSFSLDEPPL | Phf3 protein (Fragment) | Phf3 | 855 |
| IPI00620256.3 | P48678 | R.SVGGS*GGGSFGDNLVTR.S | 633 | FRSVGGSGGGSFG | Prelamin-A/C [Cleaved into: Lamin-A/C] | Lmna Lmn1 | 665 |
| IPI00130423.2 | Q9Z1S8 | K.SSLTGS*ETDNEDVYTFK.M | 282 | KSSLTGSETDNED | GRB2-associated-binding protein 2 (GRB2-associated binder 2) (Growth factor receptor bound protein 2-associated protein 2) (PH domain-containing adaptor molecule p97) | Gab2 | 665 |
| IPI00620564.1 | Q6A0A2 | R.NLST*DASTNTVPVVGPR.E | 571 | KERNLSTDASTNT | La-related protein 4B (La ribonucleoprotein domain family member 4B) (La ribonucleoprotein domain family member 5) (La-related protein 5) | Larp4b D13Wsu64e Kiaa0217 Larp5 | 741 |
| IPI00119501.1 | Q9D824 | R.DHS*PTPSVFNS*DEER.Y | 479 | ERERDHSPTPSVF | Pre-mRNA 3'-end-processing factor FIP1 (FIP1-like 1 protein) | Fip1l1 | 581 |
| IPI00775941.1 | Q3U2S4 | R.ATS*PLVSLYPALECR.A | 503 | GGDRATSPLVSLY | OTU domain-containing protein 5 (EC 3.4.19.12) (Deubiquitinating enzyme A) (DUBA) | Otud5 DXImx46e Sfc7 | 566 |
| IPI00656283.1 | Q8BQ33 | R.TAQTLLYT*PER.L | 1134 | AQTLLYTPERLQN | Treslin (TopBP1-interacting checkpoint and replication regulator) (TopBP1-interacting, replication-stimulating protein) | Ticrr | 1889 |
| IPI00656283.1 | Q8BQ33 | R.NS*LSAS*PPPGELNWK.E | 1322 | PHQPRNSLSASPP | Treslin (TopBP1-interacting checkpoint and replication regulator) (TopBP1-interacting, replication-stimulating protein) | Ticrr | 1889 |
| IPI00656283.1 | Q8BQ33 | R.SCLS*ASGLQALTQS*PLLFQGR.T | 1826 | MVRSCLSASGLQA | Treslin (TopBP1-interacting checkpoint and replication regulator) (TopBP1-interacting, replication-stimulating protein) | Ticrr | 1889 |
| IPI00648511.1 | A2AB59 | R.ASVGS*GESLETEWGQYWDEESR.R | 297 | SRASVGSGESLET | Rho GTPase-activating protein 27 (CIN85-associated multi-domain-containing Rho GTPase-activating protein 1) (Rho-type GTPase-activating protein 27) | Arhgap27 Camgap1 | 869 |
| IPI00125382.2 | Q9WV70 | K.DLFELDS*SEGEDSTDFFER.G | 673 | DLFELDSSEGEDS | Nucleolar complex protein 2 homolog (Protein NOC2 homolog) (NOC2-like protein) (Novel INHAT repressor) | Noc2l Nir | 747 |
| IPI00125382.2 | Q9WV70 | K.DLFELDS*S*EGEDSTDFFER.G | 673 | DLFELDSSEGEDS | Nucleolar complex protein 2 homolog (Protein NOC2 homolog) (NOC2-like protein) (Novel INHAT repressor) | Noc2l Nir | 747 |
| IPI00648425.1 | A2A9T0 | R.RS*PASPSCPS*PVPQR.R | 385 | SPQQRRSPASPSC | TANK-binding kinase 1-binding protein 1 (TBK1-binding protein 1) (Similar to NAP1 TBK1 adapter) | Tbkbp1 Sintbad | 611 |
| IPI00153270.1 | Q8R0T2 | R.S*PGFVPPS*PEFAPR.S | 93 | PGFESRSPGFVPP | Zinc finger protein 768 | Znf768 Zfp768 | 568 |
| IPI00118895.1 | Q61235 | R.S*PSLGSDLTFATR.T | 373 | SGSGCRSPSLGSD | Beta-2-syntrophin (59 kDa dystrophin-associated protein A1 basic component 2) (Syntrophin-3) (SNT3) (Syntrophin-like) (SNTL) | Sntb2 Snt2b2 | 520 |
| IPI00652225.1 | Q9WTK7 | R.IDS*TEVIYQPR.R | 31 | FIHRIDSTEVIYQ | Serine/threonine-protein kinase STK11 (EC 2.7.11.1) (Liver kinase B1 homolog) (LKB1) (mLKB1) | Stk11 Lkb1 | 436 |
| IPI00651958.1 | Q8K1Y2 | K.SVFPATVSAVLPAPSPCS*S*PK.T | 30 | PAPSPCSSPKTGL | Serine/threonine-protein kinase D3 (EC 2.7.11.13) (Protein kinase C nu type) (nPKC-nu) | Prkd3 Prkcn | 889 |
| IPI00266942.5 | Q3TZZ7 | K.EPTPSIAS*DIS*LPIATQELR.Q | 682 | PTPSIASDISLPI | Extended synaptotagmin-2 (E-Syt2) | Esyt2 D12Ertd551e Fam62b | 845 |
| IPI00762504.1 | Q3U214 | K.VYS*SSEFLAVQPTPTFAER.S | 731 | RFSKVYSSSEFLA | Microtubule-associated serine/threonine-protein kinase 3 (EC 2.7.11.1) | Mast3 Kiaa0561 | 1321 |
| IPI00762504.1 | Q3U214 | K.VYSSS*EFLAVQPTPTFAER.S | 733 | SKVYSSSEFLAVQ | Microtubule-associated serine/threonine-protein kinase 3 (EC 2.7.11.1) | Mast3 Kiaa0561 | 1321 |
| IPI00762504.1 | Q3U214 | K.VYSS*SEFLAVQPTPTFAER.S | 732 | FSKVYSSSEFLAV | Microtubule-associated serine/threonine-protein kinase 3 (EC 2.7.11.1) | Mast3 Kiaa0561 | 1321 |
| IPI00654257.2 | Q7TQK0 | R.S*PVGLGPEGVSSASSAR.K | 596 | PPTVLRSPVGLGP | Cyclin-T2 (CycT2) | Ccnt2 | 723 |
| IPI00651807.1 | Q6PDK2 | R.LCPQPEEPYLS*PQPEEPR.L | 727 | PEEPYLSPQPEEP | Histone-lysine N-methyltransferase 2D (Lysine N-methyltransferase 2D) (EC 2.1.1.43) (ALL1-related protein) (Myeloid/lymphoid or mixed-lineage leukemia protein 2) | Kmt2d Mll2 Mll4 | 5588 |
| IPI00121785.1 | Q921G6 | R.WSGNES*TDDFSELSFR.I | 317 | RWSGNESTDDFSE | Leucine-rich repeat and calponin homology domain-containing protein 4 | Lrch4 | 680 |
| IPI00121785.1 | Q921G6 | R.WS*GNESTDDFSELSFR.I | 313 | SGSKRWSGNESTD | Leucine-rich repeat and calponin homology domain-containing protein 4 | Lrch4 | 680 |
| IPI00331173.8 | Q3U9G9 | K.Y*GLAWEKYCQRVPYR.I | 606 | HQCRRKYGLAWEK | Lamin-B receptor (Integral nuclear envelope inner membrane protein) | Lbr | 626 |
| IPI00137805.2 | O54824 | R.ISS*FENFGSSQLPDR.G | 839 | IRQRISSFENFGS | Pro-interleukin-16 [Cleaved into: Interleukin-16 (IL-16) (Lymphocyte chemoattractant factor) (LCF)] | Il16 | 1322 |
| IPI00127264.3 | | R.TLSIST*QDLSPR.- |  |  |  |  |  |
| IPI00762364.1 | Q922U2 | R.S*LDLDSIIAEVK.A | 326 | SMDNNRSLDLDSI | Keratin, type II cytoskeletal 5 (Cytokeratin-5) (CK-5) (Keratin-5) (K5) (Type-II keratin Kb5) | Krt5 Krt2-5 | 580 |
| IPI00667973.2 | Q6A065 | R.GT*PLYGQPSWWGDAEEDEQR.A | 173 | SAMPRGTPLYGQP | Centrosomal protein of 170 kDa (Cep170) | Cep170 Kiaa0470 | 1588 |
| IPI00667973.2 | Q6A065 | R.FPTDYAS*TSEDEFGSNR.N | 1229 | FPTDYASTSEDEF | Centrosomal protein of 170 kDa (Cep170) | Cep170 Kiaa0470 | 1588 |
| IPI00670545.2 | Q6ZQ58 | R.SLPTTVPESPNY*R.N | 754 | VPESPNYRNARTP | La-related protein 1 (La ribonucleoprotein domain family member 1) | Larp1 Kiaa0731 Larp | 1072 |
| IPI00670545.2 | Q6ZQ58 | R.S*LPTTVPESPNYR.N | 743 | PSTIARSLPTTVP | La-related protein 1 (La ribonucleoprotein domain family member 1) | Larp1 Kiaa0731 Larp | 1072 |
| IPI00468179.3 | A2A482 | K.ELSESVQQQSAPVPLIS*PK.R | 524 | APVPLISPKRQIR | Zinc finger, MYND-type-containing 8 | Zmynd8 | 1206 |
| IPI00338954.3 | P59808 | R.S*CETLEGPEPVESWPR.S | 813 | PVSICRSCETLEG | SAM and SH3 domain-containing protein 1 | Sash1 | 1230 |
| IPI00652147.1 | A1L338 | R.VVDNS*PPALPPK.K | 259 | IRVVDNSPPALPP | Rapgef1 protein | Rapgef1 | 1185 |
| IPI00652147.1 | A1L338 | R.ATS*GSSLPVGINR.Q | 288 | PMSRATSGSSLPV | Rapgef1 protein | Rapgef1 | 1185 |
| IPI00480537.1 | Q8BWW9 | R.TSTFCGT*PEFLAPEVLTETSYTR.A | 819 | TSTFCGTPEFLAP | Serine/threonine-protein kinase N2 (EC 2.7.11.13) (PKN gamma) (Protein kinase C-like 2) (Protein-kinase C-related kinase 2) | Pkn2 Prk2 Prkcl2 | 983 |
| IPI00761572.1 | Q3TYA6 | K.GTVAVGDS*EEDGEDVFEVER.I | 51 | TVAVGDSEEDGED | M-phase phosphoprotein 8 | Mphosph8 Mpp8 | 858 |
| IPI00798508.1 | Q91YE7 | R.GLVAAYSGDS*DNEEELVER.L | 624 | AAYSGDSDNEEEL | RNA-binding protein 5 (Putative tumor suppressor LUCA15) (RNA-binding motif protein 5) | Rbm5 Luca15 | 815 |
| IPI00623834.1 | Q9DBT5 | R.SAPY*EFPEESPIEQLEER.R | 64 | ELRSAPYEFPEES | AMP deaminase 2 (EC 3.5.4.6) (AMP deaminase isoform L) | Ampd2 | 798 |
| IPI00623834.1 | Q9DBT5 | R.S*APYEFPEESPIEQLEER.R | 61 | AESELRSAPYEFP | AMP deaminase 2 (EC 3.5.4.6) (AMP deaminase isoform L) | Ampd2 | 798 |
| IPI00623834.1 | Q9DBT5 | R.SAPYEFPEES*PIEQLEER.R | 70 | YEFPEESPIEQLE | AMP deaminase 2 (EC 3.5.4.6) (AMP deaminase isoform L) | Ampd2 | 798 |
| IPI00752894.1 | Q9CS00 | R.S*PGTATLALSQQQSLQER.L | 148 | SPRRSRSPGTATL | Cactin | Cactin | 772 |
| IPI00406829.1 | Q9D4H2 | R.SSLDIHGDES*S*LDINVLK.D | 425 | DIHGDESSLDINV | GRIP and coiled-coil domain-containing protein 1 (Golgi coiled-coil protein 1) | Gcc1 | 778 |
| IPI00453656.1 | Q8K245 | K.VAPLSSS*LDTSLDFSK.E | 549 | VAPLSSSLDTSLD | UV radiation resistance associated protein | Uvrag Uvrag1 | 698 |
| IPI00663705.1 | Q8BYW1 | R.SS*VGWDATEDPPLSR.T | 380 | APVPRSSVGWDAT | Rho GTPase-activating protein 25 (Rho-type GTPase-activating protein 25) | Arhgap25 Kiaa0053 | 648 |
| IPI00554827.1 | Q5DTP4 | R.LWS*SPAFSPTCPFR.E | 501 | PRDRLWSSPAFSP | MKIAA4082 protein (Fragment) | Dennd4a AI115600 mKIAA4082 | 615 |
| IPI00751106.1 | Q3TAH0 | R.S*VDAYDSYWESR.K | 247 | VTKPSRSVDAYDS | |  |  |
| IPI00460124.1 | Q9DB00 | R.TS*SHASRGLPK.S | 1426 | AAEGRTSSHASRG | GON-4-like protein (GON-4 homolog) | Gon4l Gon4 Kiaa1606 | 2260 |
| IPI00553798.2 | E9Q616 | R.LRS*EDGVEGDLGETQSR.T | 136 | IKPRLRSEDGVEG | AHNAK nucleoprotein (desmoyokin) | Ahnak | 5656 |
| IPI00553798.2 | E9Q616 | R.LPSGSGPAS*PTTGSAVDIR.A | 217 | SGSGPASPTTGSA | AHNAK nucleoprotein (desmoyokin) | Ahnak | 5656 |
| IPI00553798.2 | E9Q616 | R.LPSGS*GPASPTTGSAVDIR.A | 213 | IRLPSGSGPASPT | AHNAK nucleoprotein (desmoyokin) | Ahnak | 5656 |
| IPI00553798.2 | E9Q616 | K.VSVAT*PDVSLEASEGAVK.L | 5169 | PKVSVATPDVSLE | AHNAK nucleoprotein (desmoyokin) | Ahnak | 5656 |
| IPI00553798.2 | E9Q616 | K.VSVATPDVS*LEASEGAVK.L | 5173 | VATPDVSLEASEG | AHNAK nucleoprotein (desmoyokin) | Ahnak | 5656 |
| IPI00553798.2 | E9Q616 | R.EFSAPST*PTGTLEFAGGDAK.G | 5567 | EFSAPSTPTGTLE | AHNAK nucleoprotein (desmoyokin) | Ahnak | 5656 |
| IPI00553798.2 | E9Q616 | R.EFSAPS*TPTGTLEFAGGDAK.G | 5566 | REFSAPSTPTGTL | AHNAK nucleoprotein (desmoyokin) | Ahnak | 5656 |
| IPI00553798.2 | E9Q616 | R.EFSAPST*PTGT*LEFAGGDAK.G | 5567 | EFSAPSTPTGTLE | AHNAK nucleoprotein (desmoyokin) | Ahnak | 5656 |
| IPI00551482.3 | P97868 | K.VEGTEIVKPS*PK.R | 1179 | TEIVKPSPKRKME | E3 ubiquitin-protein ligase RBBP6 (EC 2.3.2.27) (Proliferation potential-related protein) (Protein P2P-R) (RING-type E3 ubiquitin transferase RBBP6) (Retinoblastoma-binding protein 6) (p53-associated cellular protein of testis) | Rbbp6 P2pr Pact | 1790 |
| IPI00551482.3 | P97868 | K.VAGSEGSSSTLVDYTSTSSTGGS*PVR.K | 1278 | TSSTGGSPVRKSE | E3 ubiquitin-protein ligase RBBP6 (EC 2.3.2.27) (Proliferation potential-related protein) (Protein P2P-R) (RING-type E3 ubiquitin transferase RBBP6) (Retinoblastoma-binding protein 6) (p53-associated cellular protein of testis) | Rbbp6 P2pr Pact | 1790 |
| IPI00466187.4 | F8VQB6 | R.AAQEFLES*LNFDEIDECVR.N | 943 | AQEFLESLNFDEI | Unconventional myosin-X (Unconventional myosin-10) | Myo10 | 2062 |
| IPI00466187.4 | F8VQB6 | R.YSVGTYNS*SGAYR.F | 1134 | SVGTYNSSGAYRF | Unconventional myosin-X (Unconventional myosin-10) | Myo10 | 2062 |
| IPI00119242.1 | Q6ZQ03 | K.ATGGLCLLGAY*ADSDDDESDVSEK.T | 117 | LCLLGAYADSDDD | Formin-binding protein 4 (Formin-binding protein 30) | Fnbp4 Fbp30 Kiaa1014 | 1031 |
| IPI00420186.1 | Q60902 | R.STPSHGS*VSSLNSTGS*LSPK.H | 244 | STPSHGSVSSLNS | Epidermal growth factor receptor substrate 15-like 1 (Epidermal growth factor receptor pathway substrate 15-related sequence) (Eps15-rs) (Eps15-related protein) (Eps15R) | Eps15l1 Eps15-rs Eps15R | 907 |
| IPI00278312.5 | Q9Z2Z9 | R.GY*NY*ATCLEGALKIK.E | 548 | LVMGRGYNYATCL | Glutamine--fructose-6-phosphate aminotransferase [isomerizing] 2 (EC 2.6.1.16) (D-fructose-6-phosphate amidotransferase 2) (Glutamine:fructose-6-phosphate amidotransferase 2) (GFAT 2) (GFAT2) (Hexosephosphate aminotransferase 2) | Gfpt2 | 682 |
| IPI00116442.1 | Q3UMU9 | R.VMTVTAVTTTATS*DR.M | 137 | VTTTATSDRMESD | Hepatoma-derived growth factor-related protein 2 (HRP-2) | Hdgfl2 Hdgfrp2 | 669 |
| IPI00116442.1 | Q3UMU9 | R.VMTVTAVTTTAT*SDR.M | 136 | AVTTTATSDRMES | Hepatoma-derived growth factor-related protein 2 (HRP-2) | Hdgfl2 Hdgfrp2 | 669 |
| IPI00407698.1 | Q8BL80 | R.TSS*LDGPAAAVLSR.T | 397 | PTHRTSSLDGPAA | Rho GTPase-activating protein 22 (Rho-type GTPase-activating protein 22) (p68RacGAP) | Arhgap22 | 702 |
| IPI00757933.1 | | K.YGDT*IVEHGDTK.W |  |  |  |  |  |
| IPI00753462.1 | Q9EQ28 | R.QVS*ITGFFQK.K | 454 | KANRQVSITGFFQ | DNA polymerase delta subunit 3 (DNA polymerase delta subunit p66) | Pold3 | 462 |
| IPI00676937.2 | Q8BZN6 | K.DMSQS*PTSSFVR.A | 877 | EKDMSQSPTSSFV | Dedicator of cytokinesis protein 10 (Zizimin-3) | Dock10 Kiaa0694 Ziz3 | 2150 |
| IPI00676937.2 | Q8BZN6 | K.DLYPFTVNTSNQGS*R.D | 1236 | NTSNQGSRDDLST | Dedicator of cytokinesis protein 10 (Zizimin-3) | Dock10 Kiaa0694 Ziz3 | 2150 |
| IPI00676937.2 | Q8BZN6 | K.DLYPFTVNT*SNQGSR.D | 1231 | YPFTVNTSNQGSR | Dedicator of cytokinesis protein 10 (Zizimin-3) | Dock10 Kiaa0694 Ziz3 | 2150 |
| IPI00676937.2 | Q8BZN6 | R.SQT*LPIIR.G | 1440 | KQHRSQTLPIIRG | Dedicator of cytokinesis protein 10 (Zizimin-3) | Dock10 Kiaa0694 Ziz3 | 2150 |
| IPI00719994.2 | | R.LAQT*TPVDSALGSSR.H | |  |  |  |  |
| IPI00124318.4 | P29352 | R.TPESFIVVEEAGEPS*PR.V | 634 | EEAGEPSPRVTES | Tyrosine-protein phosphatase non-receptor type 22 (EC 3.1.3.48) (Hematopoietic cell protein-tyrosine phosphatase 70Z-PEP) (PEST-domain phosphatase) (PEP) | Ptpn22 Ptpn8 | 802 |
| IPI00463155.1 | Q8K1B8 | K.TAS*GDYIDSSWELR.V | 8 | AGMKTASGDYIDS | Fermitin family homolog 3 (Kindlin-3) (Unc-112-related protein 2) | Fermt3 Kind3 Urp2 | 665 |
| IPI00170055.11 | A2AKB9 | R.LPGGPAVS*PAER.A | 67 | PGGPAVSPAERAG | DDB1- and CUL4-associated factor 10 (WD repeat-containing protein 32) | Dcaf10 Wdr32 | 566 |
| IPI00331314.5 | Q3UH60 | K.LLS*PYSPQTQETDSIGQK.E | 50 | KRSKLLSPYSPQT | Disco-interacting protein 2 homolog B (DIP2 homolog B) | Dip2b Kiaa1463 | 1574 |
| IPI00331314.5 | Q3UH60 | K.GTSGS*LADVFANTR.I | 202 | VKGTSGSLADVFA | Disco-interacting protein 2 homolog B (DIP2 homolog B) | Dip2b Kiaa1463 | 1574 |
| IPI00468120.1 | Q6PDH0 | R.S*PSPTLGESLAPR.K | 520 | RGRRTRSPSPTLG | Pleckstrin homology-like domain family B member 1 (Protein LL5-alpha) | Phldb1 Kiaa0638 Ll5a | 1371 |
| IPI00468120.1 | Q6PDH0 | R.LSPAY*SLGSLTGASPR.Q | 544 | GRLSPAYSLGSLT | Pleckstrin homology-like domain family B member 1 (Protein LL5-alpha) | Phldb1 Kiaa0638 Ll5a | 1371 |
| IPI00468120.1 | Q6PDH0 | R.LSPAYS*LGSLTGASPR.Q | 545 | RLSPAYSLGSLTG | Pleckstrin homology-like domain family B member 1 (Protein LL5-alpha) | Phldb1 Kiaa0638 Ll5a | 1371 |
| IPI00468120.1 | Q6PDH0 | R.LS*PAYSLGSLTGAS*PR.Q | 541 | SFSGRLSPAYSLG | Pleckstrin homology-like domain family B member 1 (Protein LL5-alpha) | Phldb1 Kiaa0638 Ll5a | 1371 |
| IPI00468120.1 | Q6PDH0 | R.LSPAY*SLGSLTGAS*PR.Q | 544 | GRLSPAYSLGSLT | Pleckstrin homology-like domain family B member 1 (Protein LL5-alpha) | Phldb1 Kiaa0638 Ll5a | 1371 |
| IPI00127202.3 | Q91VS8 | R.LGGQTAIGVSTLEPEQS*LSPR.M | 34 | TLEPEQSLSPRMQ | FERM, ARHGEF and pleckstrin domain-containing protein 2 (FERM domain including RhoGEF) (FIR) (FERM, RhoGEF and pleckstrin domain-containing protein 2) | Farp2 Kiaa0793 | 1065 |
| IPI00420509.2 | Q6ZPF4 | R.S*IEDLQPPNALSAPFTNSLAR.S | 174 | LRSWSRSIEDLQP | Formin-like protein 3 | Fmnl3 Frl2 Kiaa2014 | 1028 |
| IPI00112963.1 | P26231 | R.TPEELDDS*DFETEDFDVR.S | 641 | PEELDDSDFETED | Catenin alpha-1 (102 kDa cadherin-associated protein) (Alpha E-catenin) (CAP102) | Ctnna1 Catna1 | 906 |
| IPI00754782.1 | | R.S*NLQGHS*RIHT*GDRPYK.C | |  |  |  |  |
| IPI00753239.1 | Q68ED3 | R.VGS*QDVSLEVSQAVGK.M | 545 | TVTRVGSQDVSLE | Non-canonical poly(A) RNA polymerase PAPD5 (EC 2.7.7.19) (PAP-associated domain-containing protein 5) (Terminal nucleotidyltransferase 4B) (Terminal uridylyltransferase 3) (TUTase 3) (Topoisomerase-related function protein 4-2) (TRF4-2) | Tent4b Papd5 | 633 |
| IPI00229798.2 | Q8CI95 | R.SFSLASSGNS*PISQR.R | 186 | LASSGNSPISQRR | Oxysterol-binding protein-related protein 11 (ORP-11) (OSBP-related protein 11) | Osbpl11 | 751 |
| IPI00648543.3 | | R.SQS*TVSSIFSEAWK.R |  |  |  |  |  |
| IPI00312702.4 | Q3UYI5 | R.VIEPPAASCPS*SPR.I | 509 | PAASCPSSPRIRR | Ral guanine nucleotide dissociation stimulator-like 3 (RalGDS-like 3) (RalGDS-related effector protein of M-Ras) (Ras pathway modulator) (RPM) | Rgl3 | 709 |
| IPI00312702.4 | Q3UYI5 | R.VIEPPAASCPSS*PR.I | 510 | AASCPSSPRIRRR | Ral guanine nucleotide dissociation stimulator-like 3 (RalGDS-like 3) (RalGDS-related effector protein of M-Ras) (Ras pathway modulator) (RPM) | Rgl3 | 709 |
| IPI00754162.1 | Q9Z2U4 | R.STS*PVTDPSMPIR.K | 187 | KNNRSTSPVTDPS | ETS-related transcription factor Elf-4 (E74-like factor 4) (Myeloid Elf-1-like factor) | Elf4 Mef | 655 |
| IPI00761774.1 | Q3TBD2 | K.SWPIS*ISDTEVGLDTSSGDLK.K | 622 | HKSWPISISDTEV | Rho GTPase-activating protein 45 (Minor histocompatibility protein HA-1) | Arhgap45 Hmha1 | 1116 |
| IPI00755697.1 | Q8BUR4 | R.FSVSPASPSSQQT*PPPVT*PR.A | 1767 | SPSSQQTPPPVTP | Dedicator of cytokinesis protein 1 (180 kDa protein downstream of CRK) (DOCK180) | Dock1 | 1865 |
| IPI00165717.1 | Q99K74 | R.LLSSS*DDDANILSSPTDR.S | 862 | MRLLSSSDDDANI | Mediator of RNA polymerase II transcription subunit 24 (Mediator complex subunit 24) (Thyroid hormone receptor-associated protein 4) (Thyroid hormone receptor-associated protein complex 100 kDa component) (Trap100) (mTRAP100) | Med24 D11Ertd307e Thrap4 Trap100 | 987 |
| IPI00121431.3 | Q60848 | K.S*INYSELDQFPSELEK.L | 498 | KRRSRKSINYSEL | Lymphocyte-specific helicase (EC 3.6.4.-) (Proliferation-associated SNF2-like protein) | Hells Lsh Pasg | 821 |
| IPI00406371.3 | P47856 | R.VDST*TCLFPVEEK.A | 260 | LSRVDSTTCLFPV | Glutamine--fructose-6-phosphate aminotransferase [isomerizing] 1 (EC 2.6.1.16) (D-fructose-6-phosphate amidotransferase 1) (Glutamine:fructose-6-phosphate amidotransferase 1) (GFAT 1) (GFAT1) (Hexosephosphate aminotransferase 1) | Gfpt1 Gfpt | 697 |
| IPI00229457.3 | Q8CGA3 | K.LCLS*TVDLEVK.C | 297 | GHKLCLSTVDLEV | Large neutral amino acids transporter small subunit 4 (L-type amino acid transporter 4) (Solute carrier family 43 member 2) | Slc43a2 Lat4 | 568 |
| IPI00230523.3 | Q9Z148 | K.LNS*GSLSEDLGSAGGSGDIILEK.G | 285 | KRRKLNSGSLSED | Histone-lysine N-methyltransferase EHMT2 (EC 2.1.1.-) (EC 2.1.1.43) (Euchromatic histone-lysine N-methyltransferase 2) (HLA-B-associated transcript 8) (Histone H3-K9 methyltransferase 3) (H3-K9-HMTase 3) (Protein G9a) | Ehmt2 Bat8 G9a Ng36 | 1263 |
| IPI00227807.2 | P00520 | K.GLGESDALDSEPAVS*PLLPR.K | 569 | DSEPAVSPLLPRK | Tyrosine-protein kinase ABL1 (EC 2.7.10.2) (Abelson murine leukemia viral oncogene homolog 1) (Abelson tyrosine-protein kinase 1) (Proto-oncogene c-Abl) (p150) | Abl1 Abl | 1123 |
| IPI00624169.3 | Q80U62 | R.STSFS*LSGPSWQPQEDR.E | 252 | RRSTSFSLSGPSW | Run domain Beclin-1-interacting and cysteine-rich domain-containing protein (Rubicon) | Rubcn Kiaa0226 | 956 |
| IPI00624169.3 | Q80U62 | R.STS*FSLSGPSWQPQEDR.E | 250 | SERRSTSFSLSGP | Run domain Beclin-1-interacting and cysteine-rich domain-containing protein (Rubicon) | Rubcn Kiaa0226 | 956 |
| IPI00652302.1 | Q8BZ03 | R.LGS*SESLPCTAEELSR.S | 211 | HSVRLGSSESLPC | Serine/threonine-protein kinase D2 (EC 2.7.11.13) (nPKC-D2) | Prkd2 | 875 |
| IPI00121225.1 | Q8VC03 | R.VLGAGSSGPAPAT*PSR.T | 882 | SGPAPATPSRTPS | Echinoderm microtubule-associated protein-like 3 (EMAP-3) | Eml3 | 897 |
| IPI00556829.1 | Q9Z1N2 | R.SSVVPSGGLT*PVYIR.R | 349 | VPSGGLTPVYIRR | Origin recognition complex subunit 1 | Orc1 Orc1l | 840 |
| IPI00321802.5 | Q3UKC1 | R.VPS*WEDNVVCSQPAR.N | 693 | PPVRVPSWEDNVV | Tax1-binding protein 1 homolog | Tax1bp1 | 814 |
| IPI00229072.1 | Q60953 | R.LATS*SPEQSWPSTFK.A | 514 | EDRLATSSPEQSW | Protein PML | Pml | 885 |
| IPI00757040.2 | Q80XI6 | R.NVFEVGAGDS*PTFPR.F | 525 | EVGAGDSPTFPRF | Mitogen-activated protein kinase kinase kinase 11 (EC 2.7.11.25) (Mixed lineage kinase 3) | Map3k11 Mlk3 | 850 |
| IPI00125980.1 | Q9WVH4 | R.SS*DELDAWTDFR.S | 299 | SPTSRSSDELDAW | Forkhead box protein O3 | Foxo3 Fkhr2 Foxo3a | 672 |
| IPI00649362.1 | Q8C2Q3 | R.AQPSVS*LGAPYR.G | 280 | RAQPSVSLGAPYR | RNA-binding protein 14 (RNA-binding motif protein 14) | Rbm14 | 669 |
| IPI00172222.1 | Q8K2W9 | R.SLGFVGS*PCR.I | 286 | SLGFVGSPCRIRG | Uncharacterized protein C14orf93 homolog | 0 | 541 |
| IPI00625056.1 | | R.SSSVGS*SSSYPISSAGPR.T | |  |  |  |  |
| IPI00625056.1 | | R.TQLAS*WSDPTEETGPVAGILDTETLEK.V | | |  |  |  |
| IPI00625056.1 | | K.GYYSPYSVSGSGSTAGS*R.T | |  |  |  |  |
| IPI00462783.1 | Q925H1 | K.GDLLVNDNPDPAPLS*PELQDFK.C | 216 | PDPAPLSPELQDF | Zinc finger transcription factor Trps1 | Trps1 | 1281 |
| IPI00757089.1 | E9Q6P5 | R.VEQALS*EVASS*LQSSAPK.Q | 673 | RVEQALSEVASSL | Tetratricopeptide repeat protein 7B (TPR repeat protein 7B) | Ttc7b | 843 |
| IPI00408119.4 | P27546 | K.ATSPS*TLVSTGPSSR.S | 787 | SKATSPSTLVSTG | Microtubule-associated protein 4 (MAP-4) | Map4 Mtap4 | 1125 |
| IPI00132122.3 | Q9CW03 | R.GSGS*QSSVPSVDQFTGVGIR.V | 1083 | SERGSGSQSSVPS | Structural maintenance of chromosomes protein 3 (SMC protein 3) (SMC-3) (Basement membrane-associated chondroitin proteoglycan) (Bamacan) (Chondroitin sulfate proteoglycan 6) (Chromosome segregation protein SmcD) (Mad member-interacting protein 1) | Smc3 Bam Bmh Cspg6 Mmip1 Smc3l1 Smcd | 1217 |
| IPI00322431.8 | Q8K394 | R.GAAGGALPTS*PSPALGAK.G | 16 | GGALPTSPSPALG | Inactive phospholipase C-like protein 2 (PLC-L(2)) (PLC-L2) (Phospholipase C-L2) (Phospholipase C-epsilon-2) (PLC-epsilon-2) | Plcl2 Kiaa1092 Plce2 | 1128 |
| IPI00225777.3 | E9Q2I4 | R.SFEPPPYT*PPPILS*PVR.E | 647 | FEPPPYTPPPILS | ELM2 and Myb/SANT-like domain-containing 1 | Elmsan1 C130039O16Rik | 1089 |
| IPI00229739.4 | Q7TSI1 | K.S*AAGLCTSPVQDTPESR.A | 459 | QEPALKSAAGLCT | Pleckstrin homology domain-containing family M member 1 (PH domain-containing family M member 1) | Plekhm1 | 1074 |
| IPI00225616.3 | Q8C4S8 | K.SY*EFEDLLQSSSENSR.V | 328 | ADHRKSYEFEDLL | DENN domain-containing protein 2A | Dennd2a | 1000 |
| IPI00129472.5 | P46062 | R.SGS*DAGEVRPPTPAS*PR.A | 53 | PLLRSGSDAGEVR | Signal-induced proliferation-associated protein 1 (Sipa-1) (GTPase-activating protein Spa-1) | Sipa1 Spa-1 Spa1 | 1037 |
| IPI00462743.4 | Q7TPS5 | K.LSSPAAFLPACSS*PSR.E | 260 | FLPACSSPSRELK | C2 domain-containing protein 5 (138 kDa C2 domain-containing phosphoprotein) | C2cd5 Cdp138 Kiaa0528 | 1016 |
| IPI00462743.4 | Q7TPS5 | K.LSSPAAFLPACS*SPSR.E | 259 | AFLPACSSPSREL | C2 domain-containing protein 5 (138 kDa C2 domain-containing phosphoprotein) | C2cd5 Cdp138 Kiaa0528 | 1016 |
| IPI00462743.4 | Q7TPS5 | K.LSSPAAFLPACSSPS*R.E | 262 | PACSSPSRELKEI | C2 domain-containing protein 5 (138 kDa C2 domain-containing phosphoprotein) | C2cd5 Cdp138 Kiaa0528 | 1016 |
| IPI00515222.1 | Q8CCN5 | R.GVSTVTDAAS*GTFDR.S | 823 | TVTDAASGTFDRS | Breast carcinoma-amplified sequence 3 homolog (K20D4) (Protein rudhira) | Bcas3 | 928 |
| IPI00471450.4 | Q66JV4 | R.SS*GEEFWTPPDFR.G | 702 | PKNFRSSGEEFWT | RNA-binding protein 12B-B (RNA-binding motif protein 12B-B) | Rbm12b2 Rbm12bb | 834 |
| IPI00742305.1 | Q9QZK2 | R.TGSEPTLS*PALVR.R | 370 | GSEPTLSPALVRR | Breast cancer anti-estrogen resistance protein 3 (p130Cas-binding protein AND-34) | Bcar3 And34 | 820 |
| IPI00308852.4 | P98078 | K.SSPNPFVGS*PPK.G | 401 | PNPFVGSPPKGLS | Disabled homolog 2 (Adaptor molecule disabled-2) (Differentially expressed in ovarian carcinoma 2) (DOC-2) (Mitogen-responsive phosphoprotein) | Dab2 Doc2 | 766 |
| IPI00331212.2 | Q9ES28 | R.KPS*DEEFAVR.K | 673 | KPERKPSDEEFAV | Rho guanine nucleotide exchange factor 7 (Beta-Pix) (PAK-interacting exchange factor beta) (p85SPR) | Arhgef7 Kiaa0142 Pak3bp | 862 |
| IPI00122594.4 | Q8CJF7 | K.LDS*SQLPLQTGLDVPAT*PR.K | 1940 | EEPKLDSSQLPLQ | Protein ELYS (Embryonic large molecule derived from yolk sac) (Protein MEL-28) (Putative AT-hook-containing transcription factor 1) | Ahctf1 Elys | 2243 |
| IPI00659200.2 | Q6ZPE2 | R.LGLGT*LSSSLSR.A | 1137 | QRLGLGTLSSSLS | Myotubularin-related protein 5 (SET-binding factor 1) (Sbf1) | Sbf1 Kiaa3020 Mtmr5 | 1867 |
| IPI00659200.2 | Q6ZPE2 | R.STST*LYSQFQTAESENR.S | 1749 | ARRSTSTLYSQFQ | Myotubularin-related protein 5 (SET-binding factor 1) (Sbf1) | Sbf1 Kiaa3020 Mtmr5 | 1867 |
| IPI00659200.2 | Q6ZPE2 | R.STS*TLYSQFQTAESENR.S | 1748 | AARRSTSTLYSQF | Myotubularin-related protein 5 (SET-binding factor 1) (Sbf1) | Sbf1 Kiaa3020 Mtmr5 | 1867 |
| IPI00652887.1 | P53564 | K.EWPSAES*PYSQSSELSLTGASR.S | 882 | EWPSAESPYSQSS | Homeobox protein cut-like 1 (CCAAT displacement protein) (CDP) (Homeobox protein cux-1) | Cux1 Cutl1 Cux Kiaa4047 | 1515 |
| IPI00652960.1 | Q6Y7W8 | R.ALSSGGSITS*PPLS*PALPK.Y | 26 | SGGSITSPPLSPA | GRB10-interacting GYF protein 2 (PERQ amino acid-rich with GYF domain-containing protein 2) (Trinucleotide repeat-containing gene 15 protein) | Gigyf2 Kiaa0642 Perq2 Tnrc15 | 1291 |
| IPI00750622.1 | Q924N4 | K.IDDIPGLSDTS*PDLSSR.S | 32 | PGLSDTSPDLSSR | Solute carrier family 12 member 6 (Electroneutral potassium-chloride cotransporter 3) (K-Cl cotransporter 3) | Slc12a6 Kcc3 | 1150 |
| IPI00330220.3 | A2ARP1 | R.ALQTS*PQPVEGTGLPR.R | 959 | DRALQTSPQPVEG | Inositol hexakisphosphate and diphosphoinositol-pentakisphosphate kinase 1 (EC 2.7.4.21) (EC 2.7.4.24) (Diphosphoinositol pentakisphosphate kinase 1) (Histidine acid phosphatase domain-containing protein 2A) (InsP6 and PP-IP5 kinase 1) (VIP1 homolog) | Ppip5k1 Hisppd2a Kiaa0377 Vip1 | 1436 |
| IPI00169806.3 | Q8K1R7 | R.SST*VTEAPIAVVTSR.T | 333 | KRPRSSTVTEAPI | Serine/threonine-protein kinase Nek9 (EC 2.7.11.1) (Nercc1 kinase) (Never in mitosis A-related kinase 9) (NimA-related protein kinase 9) | Nek9 Nercc | 984 |
| IPI00130246.1 | Q91YK2 | K.SILVS*PTGLSR.V | 672 | DKSILVSPTGLSR | Ribosomal RNA processing protein 1 homolog B (RRP1-like protein B) | Rrp1b | 724 |
| IPI00757585.1 | O88448 | R.ASS*LNFLNK.S | 575 | RMKRASSLNFLNK | Kinesin light chain 2 (KLC 2) | Klc2 | 599 |
| IPI00319830.7 | Q62261 | K.SALPAQSAAT*LPAR.T | 2186 | PAQSAATLPARTL | Spectrin beta chain, non-erythrocytic 1 (Beta-II spectrin) (Embryonic liver fodrin) (Fodrin beta chain) | Sptbn1 Elf Spnb-2 Spnb2 Sptb2 | 2363 |
| IPI00319830.7 | Q62261 | R.AQTLPTSVVTITSESS*PGK.R | 2340 | TITSESSPGKREK | Spectrin beta chain, non-erythrocytic 1 (Beta-II spectrin) (Embryonic liver fodrin) (Fodrin beta chain) | Sptbn1 Elf Spnb-2 Spnb2 Sptb2 | 2363 |
| IPI00652415.2 | Q3UH06 | K.NIEYVSSPT*AELVDAFCAPETVCR.L | 739 | EYVSSPTAELVDA | Ras-responsive element-binding protein 1 (RREB-1) (RAS-responsive zinc finger transcription factor RREB) | Rreb1 | 1700 |
| IPI00623304.3 | O08648 | R.LES*EEDSIGWGTADCGPEASR.H | 492 | KLERLESEEDSIG | Mitogen-activated protein kinase kinase kinase 4 (EC 2.7.11.25) (MAPK/ERK kinase kinase 4) (MEK kinase 4) (MEKK 4) | Map3k4 Mekk4 | 1597 |
| IPI00754425.1 | Q6PAR5 | R.LQELESCSGLGSTS*DDTDVR.E | 748 | SGLGSTSDDTDVR | GTPase-activating protein and VPS9 domain-containing protein 1 (GAPex-5) (Rab5-activating protein 6) | Gapvd1 Gapex5 Kiaa1521 | 1458 |
| IPI00466371.2 | Q3V3R4 | K.EY*AQRIPS*GGDGKT*LK.F | 616 | KTIRKEYAQRIPS | Integrin alpha-1 (CD49 antigen-like family member A) (Laminin and collagen receptor) (VLA-1) (CD antigen CD49a) | Itga1 | 1179 |
| IPI00660405.1 | Q6P1G0 | K.TFPAGSSLEEASLSS*PK.G | 646 | EEASLSSPKGSSE | HEAT repeat-containing protein 6 | Heatr6 | 1184 |
| IPI00754509.1 | Q3UHD9 | R.ALSTDCTPSGDLS*PLSR.E | 812 | TPSGDLSPLSREP | Arf-GAP with GTPase, ANK repeat and PH domain-containing protein 2 (AGAP-2) (Centaurin-gamma-1) (Cnt-g1) (Phosphatidylinositol 3-kinase enhancer) (PIKE) | Agap2 Centg1 Kiaa0167 | 1186 |
| IPI00621873.2 | Q66T02 | R.SLS*ELCLISVAPGVR.T | 964 | APSRSLSELCLIS | Pleckstrin homology domain-containing family G member 5 (PH domain-containing family G member 5) (Synectin-binding RhoA exchange factor) (SYX) | Plekhg5 Kiaa0720 Syx | 1073 |
| IPI00225469.1 | P97305 | R.ETS*VDDGLGSQYPLK.K | 372 | SPSRETSVDDGLG | Nuclear factor of activated T-cells, cytoplasmic 3 (NF-ATc3) (NFATc3) (NFATx) (T-cell transcription factor NFAT4) (NF-AT4) | Nfatc3 Nfat4 | 1075 |
| IPI00671847.2 | Q9DBR7 | K.TGS*YGALAEISASK.E | 445 | GLRKTGSYGALAE | Protein phosphatase 1 regulatory subunit 12A (Myosin phosphatase-targeting subunit 1) (Myosin phosphatase target subunit 1) | Ppp1r12a Mypt1 | 1029 |
| IPI00648568.1 | Q8BYG9 | R.VEPQSVS*LS*WR.E | 471 | VEPQSVSLSWREP | Ephrin type-A receptor 10 (EC 2.7.10.1) | Epha10 | 1007 |
| IPI00227790.1 | P24604 | K.YNLFESS*IR.K | 152 | YNLFESSIRKTLP | Tyrosine-protein kinase Tec (EC 2.7.10.2) | Tec | 630 |
| IPI00169960.3 | | K.T*GETDSS*NC.- |  |  |  |  |  |
| IPI00654074.2 | Q921C3 | R.GPEVEGS*PVSEALR.E | 1830 | GPEVEGSPVSEAL | Bromodomain and WD repeat-containing protein 1 (WD repeat-containing protein 9) | Brwd1 Wdr9 | 2304 |
| IPI00653910.1 | Q924A2 | R.AVS*PAVPFSR.S | 766 | AGFRAVSPAVPFS | Protein capicua homolog | Cic Kiaa0306 | 2510 |
| IPI00653910.1 | Q924A2 | K.AQSVS*PVQATPSGGSAQLLPGK.V | 1809 | PKAQSVSPVQATP | Protein capicua homolog | Cic Kiaa0306 | 2510 |
| IPI00665494.1 | Q8VDD9 | R.AQS*YDIQAWK.K | 1315 | LRNRAQSYDIQAW | PH-interacting protein (PHIP) (IRS-1 PH domain-binding protein) (Neuronal differentiation-related protein) (NDRP) (WD repeat-containing protein 11) | Phip Ndrp Phip1 Wdr11 | 1821 |
| IPI00665494.1 | Q8VDD9 | K.SEVSTS*PFSIPTR.S | 1476 | KSEVSTSPFSIPT | PH-interacting protein (PHIP) (IRS-1 PH domain-binding protein) (Neuronal differentiation-related protein) (NDRP) (WD repeat-containing protein 11) | Phip Ndrp Phip1 Wdr11 | 1821 |
| IPI00110435.2 | Q80TM9 | R.ACS*DSLESIPAGQVASDDLR.D | 543 | PIARACSDSLESI | Nischarin (Imidazoline receptor 1) (I-1) (IR1) (Imidazoline receptor I-1-like protein) (Imidazoline-1 receptor) (I1R) | Nisch Kiaa0975 | 1593 |
| IPI00757643.1 | | R.TVS*QQSFDGVSLDSGGPDDR.I | |  |  |  |  |
| IPI00408190.1 | O08784 | K.AGAVTSSASLS*SPALAK.G | 592 | TSSASLSSPALAK | Treacle protein (Treacher Collins syndrome protein homolog) | Tcof1 | 1320 |
| IPI00172197.2 | Q8K298 | K.AS*SPVTAATFITENR.E | 292 | SSSVKASSPVTAA | Anillin | Anln | 1121 |
| IPI00172197.2 | Q8K298 | K.ASS*PVTAATFITENR.E | 293 | SSVKASSPVTAAT | Anillin | Anln | 1121 |
| IPI00462728.2 | D3YTT6 | R.QDS*WEVVEGLR.G | 33 | QGSRQDSWEVVEG | Oxysterol-binding protein | Osbpl3 | 886 |
| IPI00420256.2 | O08696 | K.VGGLDFS*PVR.T | 635 | VGGLDFSPVRTPQ | Forkhead box protein M1 (Forkhead homolog 16) (Winged-helix transcription factor Trident) | Foxm1 Fkh16 | 760 |
| IPI00410764.1 | Q80VY9 | K.MSMPIMS*SR.G | 603 | MSMPIMSSRGDME | ATP-dependent RNA helicase DHX33 (EC 3.6.4.13) (DEAH box protein 33) | Dhx33 | 698 |
| IPI00719977.1 | E9Q5C9 | K.LETPNT*FPK.R | 613 | KLETPNTFPKRKK | Nucleolar and coiled-body phosphoprotein 1 (140 kDa nucleolar phosphoprotein) (Nopp140) | Nolc1 Kiaa0035 | 702 |
| IPI00648273.1 | Q8R1A4 | R.GS*WACSIFDLK.N | 190 | DDTPRGSWACSIF | Dedicator of cytokinesis protein 7 (Protein moonlight) | Dock7 Gm430 Kiaa1771 Mnlt | 2130 |
| IPI00648273.1 | Q8R1A4 | R.S*PSGSAFGSQENLR.W | 1420 | RGQLERSPSGSAF | Dedicator of cytokinesis protein 7 (Protein moonlight) | Dock7 Gm430 Kiaa1771 Mnlt | 2130 |
| IPI00551498.1 | Q5SNZ0 | K.DTTS*FEDISPQGISDDSSTGSR.V | 1562 | NNKDTTSFEDISP | Girdin (Akt phosphorylation enhancer) (APE) (Coiled-coil domain-containing protein 88A) (G alpha-interacting vesicle-associated protein) (GIV) (Girders of actin filament) (Hook-related protein 1) (HkRP1) | Ccdc88a Grdn Kiaa1212 | 1873 |
| IPI00330385.4 | Q80UV9 | R.DASVYQDES*NLSVLDIPSATSEK.Q | 1690 | SVYQDESNLSVLD | Transcription initiation factor TFIID subunit 1 (EC 2.3.1.48) (EC 2.7.11.1) (Cell cycle gene 1 protein) (TBP-associated factor 250 kDa) (p250) (Transcription initiation factor TFIID 250 kDa subunit) (TAF(II)250) (TAFII-250) (TAFII250) | Taf1 Ccg1 | 1891 |
| IPI00785472.2 | Q8BHZ4 | K.GSPSVAASS*PPAIPK.V | 366 | PSVAASSPPAIPK | Zinc finger protein 592 (Zfp-592) | Znf592 Kiaa0211 Zfp592 | 1262 |
| IPI00622704.3 | Q148V7 | R.AGS*ISTLDS*LDFAR.Y | 180 | QLNRAGSISTLDS | RAB11-binding protein RELCH (LisH domain and HEAT repeat-containing protein KIAA1468) (RAB11-binding protein containing LisH, coiled-coil, and HEAT repeats) | Relch Kiaa1468 | 1216 |
| IPI00227516.4 | Q148V8 | R.LAS*ATANALYSSNLR.D | 1057 | SRVRLASATANAL | Protein FAM83H | Fam83h | 1209 |
| IPI00474474.2 | | R.SKS*VIEQVSWDN.- |  |  |  |  |  |
| IPI00752067.1 | O70405 | K.T*PSSQNLLTLLAR.Q | 635 | SFDFPKTPSSQNL | Serine/threonine-protein kinase ULK1 (EC 2.7.11.1) (Serine/threonine-protein kinase Unc51.1) (Unc-51-like kinase 1) | Ulk1 | 1051 |
| IPI00323820.4 | P97310 | R.ISDPLTSS*PGR.S | 27 | SDPLTSSPGRSSR | DNA replication licensing factor MCM2 (EC 3.6.4.12) (Minichromosome maintenance protein 2 homolog) (Nuclear protein BM28) | Mcm2 Bm28 Cdcl1 Kiaa0030 Mcmd2 | 904 |
| IPI00408258.4 | Q6P1D7 | K.S*PPIDLTQSVPEPLS*PR.A | 926 | LISPAKSPPIDLT | Structure-specific endonuclease subunit SLX4 (BTB/POZ domain-containing protein 12) | Slx4 Btbd12 | 1565 |
| IPI00755614.1 | Q4QRL3 | R.VQSS*LCLGDETLAGGQR.R | 1385 | MRRVQSSLCLGDE | Coiled-coil domain-containing protein 88B (Gipie) (Hook-related protein 3) (HkRP3) | Ccdc88b Ccdc88 | 1481 |
| IPI00454192.1 | Q80U22 | R.LSEGPAALAGPAS*PPR.R | 534 | ALAGPASPPRRVT | Iporin (Interacting protein of Rab1) (RUN and SH3 domain-containing protein 2) | Rusc2 Kiaa0375 | 1514 |
| IPI00677379.1 | P53349 | R.APS*PDGFSPYSPEETSR.R | 287 | PPRRAPSPDGFSP | Mitogen-activated protein kinase kinase kinase 1 (EC 2.7.11.25) (MAPK/ERK kinase kinase 1) (MEK kinase 1) (MEKK 1) | Map3k1 Mekk Mekk1 | 1493 |
| IPI00624652.2 | Q80Z38 | R.RELDRFSLDSEDVY*S*R.S | 593 | LDSEDVYSRSPAP | SH3 and multiple ankyrin repeat domains protein 2 (Shank2) (Cortactin-binding protein 1) (CortBP1) | Shank2 Cortbp1 Kiaa1022 | 1476 |
| IPI00756424.2 | Q05D44 | R.ELEELS*LEAQGIR.A | 66 | RELEELSLEAQGI | Eukaryotic translation initiation factor 5B (eIF-5B) (EC 3.6.5.3) (Translation initiation factor IF-2) | Eif5b If2 | 1216 |
| IPI00608085.1 | Q3U0M1 | K.FNFESVPES*PGEK.G | 953 | FESVPESPGEKGH | Trafficking protein particle complex subunit 9 (NIK- and IKBKB-binding protein) | Trappc9 Kiaa1882 Nibp | 1148 |
| IPI00416732.1 | Q80TM6 | K.ASS*FSGISILTR.G | 381 | PVTKASSFSGISI | R3H domain-containing protein 2 | R3hdm2 Kiaa1002 | 1044 |
| IPI00460719.4 | Q6P1H6 | R.GICDYLPS*PNK.T | 266 | ICDYLPSPNKTTP | Ankyrin repeat and LEM domain-containing protein 2 (LEM domain-containing protein 4) | Ankle2 D5Ertd585e Kiaa0692 Lem4 | 964 |
| IPI00124959.1 | E9PVX6 | K.LPSSS*PPLEPTDTSVTSR.R | 2425 | AKLPSSSPPLEPT | Proliferation marker protein Ki-67 (Antigen identified by monoclonal antibody Ki-67 homolog) (Antigen KI-67 homolog) (Antigen Ki67 homolog) | Mki67 | 3177 |
| IPI00124959.1 | E9PVX6 | K.LPSS*SPPLEPTDTSVTSR.R | 2424 | LAKLPSSSPPLEP | Proliferation marker protein Ki-67 (Antigen identified by monoclonal antibody Ki-67 homolog) (Antigen KI-67 homolog) (Antigen Ki67 homolog) | Mki67 | 3177 |
| IPI00468765.3 | A6H8H2 | R.EDVETGLDPLS*LLATECVEK.T | 1202 | TGLDPLSLLATEC | DENN domain-containing protein 4C | Dennd4c | 1906 |
| IPI00750623.1 | Q80U93 | K.SSAS*VTGEPPLYPTGSDSSR.A | 492 | SFKSSASVTGEPP | Nuclear pore complex protein Nup214 (214 kDa nucleoporin) (Nucleoporin Nup214) | Nup214 Kiaa0023 | 2085 |
| IPI00606189.3 | Q3UXZ9 | K.DLDLEPLS*DLEEGLEESR.D | 1111 | LDLEPLSDLEEGL | Lysine-specific demethylase 5A (EC 1.14.11.-) (Histone demethylase JARID1A) (Jumonji/ARID domain-containing protein 1A) (Retinoblastoma-binding protein 2) (RBBP-2) | Kdm5a Jarid1a Rbp2 | 1690 |
| IPI00229626.7 | Q8CHC4 | R.SAEDLDLLNAS*FQDESK.I | 830 | LDLLNASFQDESK | Synaptojanin-1 (EC 3.1.3.36) (Synaptic inositol 1,4,5-trisphosphate 5-phosphatase 1) | Synj1 Kiaa0910 | 1574 |
| IPI00749576.1 | Q5PSV9 | R.VLLAADS*EEEGDFPSGR.C | 168 | VLLAADSEEEGDF | Mediator of DNA damage checkpoint protein 1 | Mdc1 Kiaa0170 | 1707 |
| IPI00124298.2 | P97393 | R.GSEEDPLLS*PVETWK.G | 1201 | EEDPLLSPVETWK | Rho GTPase-activating protein 5 (Rho-type GTPase-activating protein 5) (p190-B) | Arhgap5 Rhogap5 | 1501 |
| IPI00377729.3 | Q80U44 | R.SGILIS*DAELDAFLK.E | 518 | RSGILISDAELDA | Zinc finger FYVE domain-containing protein 16 (Endofin) (Endosomal-associated FYVE domain protein) | Zfyve16 Kiaa0305 | 1528 |
| IPI00556693.1 | Q69ZA1 | R.GGDVS*PSPYSSSSWR.R | 384 | ERGGDVSPSPYSS | Cyclin-dependent kinase 13 (EC 2.7.11.22) (EC 2.7.11.23) (CDC2-related protein kinase 5) (Cell division cycle 2-like protein kinase 5) (Cell division protein kinase 13) | Cdk13 Cdc2l5 Kiaa1791 | 1511 |
| IPI00776253.1 | Q5XJV7 | R.TESQSLLQQSS*SPFR.G | 1258 | SLLQQSSSPFRGH | SET domain-containing protein 5 | Setd5 Kiaa1757 | 1441 |
| IPI00227135.2 | Q8BYJ6 | R.S*LTSSLENIFSR.G | 573 | KNKAKRSLTSSLE | TBC1 domain family member 4 (Akt substrate of 160 kDa) (AS160) | Tbc1d4 As160 Kiaa0603 | 1307 |
| IPI00227135.2 | Q8BYJ6 | R.SLTSS*LENIFSR.G | 577 | KRSLTSSLENIFS | TBC1 domain family member 4 (Akt substrate of 160 kDa) (AS160) | Tbc1d4 As160 Kiaa0603 | 1307 |
| IPI00469950.3 | P59281 | R.FLS*LEYSPVGK.E | 380 | CPERFLSLEYSPV | Rho GTPase-activating protein 39 | Arhgap39 D15Wsu169e Kiaa1688 | 1107 |
| IPI00652115.1 | Q91ZW3 | K.QNLLS*VGDYR.H | 136 | EKQNLLSVGDYRH | SWI/SNF-related matrix-associated actin-dependent regulator of chromatin subfamily A member 5 (EC 3.6.4.-) (Sucrose nonfermenting protein 2 homolog) (mSnf2h) | Smarca5 Snf2h | 1051 |
| IPI00381244.6 | Q6PDK2 | K.ASEPLLS*PPPFGESR.K | 2231 | ASEPLLSPPPFGE | Histone-lysine N-methyltransferase 2D (Lysine N-methyltransferase 2D) (EC 2.1.1.43) (ALL1-related protein) (Myeloid/lymphoid or mixed-lineage leukemia protein 2) | Kmt2d Mll2 Mll4 | 5588 |
| IPI00381244.6 | Q6PDK2 | R.ASQVEPQS*PGLGLR.A | 2299 | SQVEPQSPGLGLR | Histone-lysine N-methyltransferase 2D (Lysine N-methyltransferase 2D) (EC 2.1.1.43) (ALL1-related protein) (Myeloid/lymphoid or mixed-lineage leukemia protein 2) | Kmt2d Mll2 Mll4 | 5588 |
| IPI00381244.6 | Q6PDK2 | R.VS*PAAAQLADTFFGK.G | 4410 | LPPGRVSPAAAQL | Histone-lysine N-methyltransferase 2D (Lysine N-methyltransferase 2D) (EC 2.1.1.43) (ALL1-related protein) (Myeloid/lymphoid or mixed-lineage leukemia protein 2) | Kmt2d Mll2 Mll4 | 5588 |
| IPI00421052.1 | Q6KCD5 | R.DVPPDILLDS*PER.K | 318 | PDILLDSPERKQK | Nipped-B-like protein (Delangin homolog) (SCC2 homolog) | Nipbl Scc2 | 2798 |
| IPI00263048.1 | E9Q7G0 | R.TQPDGTSVPGEPAS*PISQR.L | 1739 | VPGEPASPISQRL | Nuclear mitotic apparatus protein 1 (Nuclear mitotic apparatus protein) (NuMA protein) | Numa1 | 2094 |
| IPI00117274.1 | Q8C3J5 | K.VEEEPIS*PGSTLPEVK.L | 1683 | VEEEPISPGSTLP | Dedicator of cytokinesis protein 2 (Protein Hch) | Dock2 | 1828 |
| IPI00648459.1 | A2BH40 | R.GPS*PSPVGSPASVAQSR.S | 697 | PGIRGPSPSPVGS | AT-rich interactive domain-containing protein 1A (ARID domain-containing protein 1A) (BRG1-associated factor 250) (BAF250) (BRG1-associated factor 250a) (BAF250A) (Osa homolog 1) (SWI-like protein) (SWI/SNF complex protein p270) (SWI/SNF-related, matrix-associated, actin-dependent regulator of chromatin subfamily F member 1) | Arid1a Baf250 Baf250a Osa1 Smarcf1 | 2283 |
| IPI00651871.2 | A1L3T8 | R.IVEYS*PPSAPR.R | 128 | VRIVEYSPPSAPR | Brd1 protein | Brd1 | 259 |
| IPI00317465.2 | Q80WQ8 | R.AYVLIT*PLR.N | 516 | RAYVLITPLRNKK | Mis18-binding protein 1 (Kinetochore-associated protein KNL-2 homolog) | Mis18bp1 Kiaa1903 Knl2 M18bp1 | 998 |
| IPI00170232.2 | Q8K4L3 | R.Y*QTQPVTLGEVEQVQSGK.L | 809 | RRMNARYQTQPVT | Supervillin (Archvillin) (p205/p250) | Svil | 2170 |
| IPI00417154.2 | Q80XI3 | R.TSS*PTSLPPLAR.S | 267 | EPPRTSSPTSLPP | Eukaryotic translation initiation factor 4 gamma 3 (eIF-4-gamma 3) (eIF-4G 3) (eIF4G 3) (eIF-4-gamma II) (eIF4GII) | Eif4g3 | 1579 |
| IPI00659248.1 | Q8C1B1 | R.SESVEGFLS*PSR.C | 1291 | SVEGFLSPSRCGS | Calmodulin-regulated spectrin-associated protein 2 (Calmodulin-regulated spectrin-associated protein 1-like protein 1) | Camsap2 Camsap1l1 Kiaa1078 | 1461 |
| IPI00659248.1 | Q8C1B1 | R.SES*VEGFLSPSR.C | 1285 | RTPRSESVEGFLS | Calmodulin-regulated spectrin-associated protein 2 (Calmodulin-regulated spectrin-associated protein 1-like protein 1) | Camsap2 Camsap1l1 Kiaa1078 | 1461 |
| IPI00648909.1 | | R.SNS*AWQIYLQR.R |  |  |  |  |  |
| IPI00474255.2 | Q8C033 | R.SVDSS*LCDLLR.D | 1280 | HRSVDSSLCDLLR | Rho guanine nucleotide exchange factor 10 | Arhgef10 Kiaa0294 | 1345 |
| IPI00474255.2 | Q8C033 | R.S*VDSSLCDLLR.D | 1276 | SSLEHRSVDSSLC | Rho guanine nucleotide exchange factor 10 | Arhgef10 Kiaa0294 | 1345 |
| IPI00467906.2 | Q6NZL6 | R.LTS*LDGWCAR.T | 873 | KQSRLTSLDGWCA | Tonsoku-like protein (Inhibitor of kappa B-related protein) (I-kappa-B-related protein) (IkappaBR) (NF-kappa-B inhibitor-like protein 2) (Nuclear factor of kappa light polypeptide gene enhancer in B-cells inhibitor-like 2) | Tonsl Ikbr Nfkbil2 | 1363 |
| IPI00462727.5 | O35071 | R.YPPYT*TPPR.M | 1079 | PRYPPYTTPPRMR | Kinesin-like protein KIF1C | Kif1c | 1100 |
| IPI00315032.8 | P55200 | R.AVFGESGGGGGSGEDEQFLGFGS*DEEVR.V | 151 | QFLGFGSDEEVRV | Histone-lysine N-methyltransferase 2A (Lysine N-methyltransferase 2A) (EC 2.1.1.43) (ALL-1) (Myeloid/lymphoid or mixed-lineage leukemia) (Myeloid/lymphoid or mixed-lineage leukemia protein 1) (Zinc finger protein HRX) [Cleaved into: MLL cleavage product N320 (N-terminal cleavage product of 320 kDa) (p320); MLL cleavage product C180 (C-terminal cleavage product of 180 kDa) (p180)] | Kmt2a All1 Hrx Mll Mll1 | 3966 |
| IPI00315032.8 | P55200 | R.AVFGES*GGGGGSGEDEQFLGFGSDEEVR.V | 134 | RAVFGESGGGGGS | Histone-lysine N-methyltransferase 2A (Lysine N-methyltransferase 2A) (EC 2.1.1.43) (ALL-1) (Myeloid/lymphoid or mixed-lineage leukemia) (Myeloid/lymphoid or mixed-lineage leukemia protein 1) (Zinc finger protein HRX) [Cleaved into: MLL cleavage product N320 (N-terminal cleavage product of 320 kDa) (p320); MLL cleavage product C180 (C-terminal cleavage product of 180 kDa) (p180)] | Kmt2a All1 Hrx Mll Mll1 | 3966 |
| IPI00399954.2 | P97412 | R.WPS*LVDR.N | 2254 | VASRWPSLVDRNA | Lysosomal-trafficking regulator (Beige protein) (CHS1 homolog) | Lyst Bg Chs1 | 3788 |
| IPI00399954.2 | P97412 | R.NADDWENFTFS*PAYEASYNR.A | 2269 | WENFTFSPAYEAS | Lysosomal-trafficking regulator (Beige protein) (CHS1 homolog) | Lyst Bg Chs1 | 3788 |
| IPI00623970.2 | P42859 | R.SGS*IVELLAGGGSSCS*PVLSR.K | 398 | GRGRSGSIVELLA | Huntingtin (Huntington disease protein homolog) (HD protein homolog) | Htt Hd Hdh | 3119 |
| IPI00756871.1 | B1AY13 | R.TIS*AQDTLAYATALLNEK.E | 2558 | TFQRTISAQDTLA | Ubiquitin carboxyl-terminal hydrolase 24 (EC 3.4.19.12) (Deubiquitinating enzyme 24) (Ubiquitin thioesterase 24) (Ubiquitin-specific-processing protease 24) | Usp24 | 2617 |
| IPI00626578.2 | Q09XV5 | R.TTGYPSS*PATTTSGTALR.L | 2520 | TTGYPSSPATTTS | Chromodomain-helicase-DNA-binding protein 8 (CHD-8) (EC 3.6.4.12) (ATP-dependent helicase CHD8) (Axis duplication inhibitor) (Duplin) | Chd8 Kiaa1564 | 2582 |
| IPI00229766.1 | Q9QY06 | K.LASAVLSQS*LDLSEK.H | 1302 | SAVLSQSLDLSEK | Unconventional myosin-IXb (Unconventional myosin-9b) | Myo9b Myr5 | 2114 |
| IPI00460042.3 | Q6GYP7 | R.SSS*TSDILEPFTVER.A | 796 | PLPRSSSTSDILE | Ral GTPase-activating protein subunit alpha-1 (GAP-related-interacting partner to E12) (GRIPE) (GTPase-activating RapGAP domain-like 1) (Tuberin-like protein 1) (p240) | Ralgapa1 Garnl1 Kiaa0884 Tulip1 | 2035 |
| IPI00408894.3 | Q61194 | K.ASVCNLQIS*PK.S | 261 | VCNLQISPKSEDI | Phosphatidylinositol 4-phosphate 3-kinase C2 domain-containing subunit alpha (PI3K-C2-alpha) (PtdIns-3-kinase C2 subunit alpha) (EC 2.7.1.154) (Cpk-m) (Phosphoinositide 3-kinase-C2-alpha) (p170) | Pik3c2a Cpk | 1686 |
| IPI00752672.1 | B1AYB6 | R.TDPLGS*PDVFTR.N | 246 | RTDPLGSPDVFTR | Methyl-CpG-binding domain protein 5 (Methyl-CpG-binding protein MBD5) | Mbd5 | 1498 |
| IPI00648738.1 | B2RUJ6 | K.GLIVYCVTS*PK.K | 1503 | IVYCVTSPKKGDR | Rap guanine nucleotide exchange factor (GEF) 6 (Rapgef6 protein) | Rapgef6 | 1606 |
| IPI00515195.1 | Q6NZJ6 | R.EATLPPVS*PPK.A | 1231 | ATLPPVSPPKAAL | Eukaryotic translation initiation factor 4 gamma 1 (eIF-4-gamma 1) (eIF-4G 1) (eIF-4G1) | Eif4g1 | 1600 |
| IPI00317401.5 | Q4VA53 | K.APS*PSQPPKK.I | 1381 | RPSKAPSPSQPPK | Sister chromatid cohesion protein PDS5 homolog B (Androgen-induced proliferation inhibitor) (Androgen-induced prostate proliferative shutoff-associated protein AS3) | Pds5b Aprin As3 Kiaa0979 | 1446 |
| IPI00719910.3 | E9Q394 | R.S*AVLLADEATAAPMFTNR.R | 1839 | PKERPRSAVLLAD | A-kinase anchor protein 13 (AKAP-13) (AKAP-Lbc) | Akap13 Brx | 2776 |
| IPI00620682.1 | Q60974 | R.ES*PVSAPLEGLICR.A | 1274 | GMSMRESPVSAPL | Nuclear receptor corepressor 1 (N-CoR) (N-CoR1) (Retinoid X receptor-interacting protein 13) (RIP13) | Ncor1 Rxrip13 | 2453 |
| IPI00749729.1 | A2A884 | K.WALAGPCS*PSADK.S | 2053 | ALAGPCSPSADKS | Transcription factor HIVEP3 (Human immunodeficiency virus type I enhancer-binding protein 3 homolog) (KB-binding and recognition component) (Kappa-B and V(D)J recombination signal sequences-binding protein) (Kappa-binding protein 1) (KBP-1) (Recombinant component) (Schnurri-3) (Zinc finger protein ZAS3) | Hivep3 KBP1 Kiaa1555 Krc Rc shn3 Zas3 | 2348 |
| IPI00776296.1 | A2AF47 | R.DEFPCGFVS*PTNR.G | 1208 | FPCGFVSPTNRGS | Dedicator of cytokinesis protein 11 (Activated Cdc42-associated guanine nucleotide exchange factor) (ACG) (Zizimin-2) | Dock11 Ziz2 | 2073 |
| IPI00776296.1 | A2AF47 | R.DEFPCGFVSPT*NR.G | 1210 | CGFVSPTNRGSLA | Dedicator of cytokinesis protein 11 (Activated Cdc42-associated guanine nucleotide exchange factor) (ACG) (Zizimin-2) | Dock11 Ziz2 | 2073 |
| IPI00623570.3 | G5E870 | R.S*ESPPAELPSLR.R | 310 | KSTKKRSESPPAE | E3 ubiquitin-protein ligase TRIP12 (EC 2.3.2.26) (HECT-type E3 ubiquitin transferase TRIP12) (Thyroid receptor-interacting protein 12) (TR-interacting protein 12) (TRIP-12) | Trip12 | 2025 |
| IPI00670480.1 | B2RY04 | R.NS*AEIAPPLPVR.R | 1832 | ESSQRNSAEIAPP | Dedicator of cytokinesis protein 5 (Lens rupture protein 2) (Rupture of lens cataract protein) | Dock5 Lr2 Rlc | 1868 |
| IPI00664305.1 | A2AIV2 | R.SFLSEPS*SPGR.S | 1577 | SFLSEPSSPGRSK | Protein virilizer homolog | Virma Kiaa1429 | 1811 |
| IPI00378684.3 | A2A7B5 | R.SDS*PAWSLSGR.D | 777 | LESRSDSPAWSLS | PR domain-containing 2, with ZNF domain | Prdm2 | 1709 |
| IPI00675346.1 | Q8C170 | R.GNYPSPSS*PVIVR.L | 2296 | NYPSPSSPVIVRL | Unconventional myosin-IXa (Unconventional myosin-9a) | Myo9a Myr7 | 2542 |
| IPI00322707.5 | Q61687 | K.LTPVSLSNS*PIK.G | 590 | PVSLSNSPIKGVD | Transcriptional regulator ATRX (EC 3.6.4.12) (ATP-dependent helicase ATRX) (HP1 alpha-interacting protein) (HP1-BP38 protein) (Heterochromatin protein 2) (X-linked nuclear protein) | Atrx Hp1bp2 Xnp | 2476 |
| IPI00475383.2 | Q8C147 | K.GPLTS*CDFDLR.S | 197 | GKGPLTSCDFDLR | Dedicator of cytokinesis protein 8 | Dock8 | 2100 |
| IPI00127764.1 | Q9R0L6 | R.INFS*DLDQR.S | 110 | KQRINFSDLDQRS | Pericentriolar material 1 protein (PCM-1) (mPCM-1) | Pcm1 | 2025 |
| IPI00753321.1 | E9Q6J5 | K.LGIPETIS*PR.N | 2968 | GIPETISPRNRQK | Biorientation of chromosomes in cell division protein 1-like 1 | Bod1l Kiaa1327 | 3032 |
