# Supplementary material for: LKB1‐MARK2 signalling mediates lipopolysaccharide‐induced production of cytokines in mouse macrophages
Source: J Cell Mol Med. 2020 Aug 25;24(19):11307–17. doi: 10.1111/jcmm.15710 (PMC7576310; doi:10.1111/jcmm.15710)
Supplement: Supplementary file 5 — Table S3 [file JCMM-24-11307-s005.doc]

**Supplemental Table 3.** Results for IL-8 driven luciferase reporter assay

| **Gene** | **Protein** | **Fold change of IL-8 activity** |
| --- | --- | --- |
| Braf | Serine/threonine-protein kinase B-raf | 0.89±0.15 |
| Btk | Tyrosine-protein kinase BTK | 1.13±0.08 |
| Cdk1 | Cyclin-dependent kinase 1 | 1.08±0.12 |
| Cdk5 | Cell division protein kinase 5 | 1.09±0.06 |
| Cdk11B | Cyclin-dependent kinase 11B | 0.70±0.22 |
| Cdk13 | Cyclin-dependent kinase 13 | 0.82±0.10 |
| Csnk1d | Casein kinase I isoform delta | 0.93±0.05 |
| Dyrk1a | Dual specificity tyrosine-phosphorylation-regulated kinase 1A | 1.02±0.10 |
| Epha10 | Ephrin type-A receptor 10 | 0.87±0.13 |
| Gprk6 | G protein-coupled receptor kinase 6 | 0.51±0.16 |
| Inpp5d | Inositol polyphosphate-5-phosphatase D | 1.03±0.19 |
| Irak3 | Interleukin-1 receptor-associated kinase 3 | 1.16±0.10 |
| Itpkb | inositol 1,4,5-trisphosphate 3-kinase B | 0.74±0.19 |
| Map3k1 | Mitogen-activated protein kinase kinase kinase 1 | 0.97±0.14 |
| Map3k3 | Mitogen-activated protein kinase kinase kinase 3 | 0.92±0.11 |
| Map3k4 | Mitogen-activated protein kinase kinase kinase 4 | 1.09±0.22 |
| Map3k7 | TAK1, Mitogen-activated protein kinase kinase kinase 7 | 0.85±0.14 |
| Map3k20 | Mitogen-activated protein kinase kinase kinase 20 | 0.64±0.05 |
| Map4k1 | Mitogen-activated protein kinase kinase kinase kinase 1 | 0.97±0.03 |
| Mark2 | Serine/threonine-protein kinase MARK2 | 0.68±0.16 |
| Mast3 | Microtubule-associated serine/threonine-protein kinase 3 | 0.79±0.01 |
| Mastl | Microtubule-associated serine/threonine-protein kinase-like | 0.80±0.10 |
| Melk | Maternal embryonic leucine zipper kinase | 0.81±0.19 |
| Mtmr2 | Myotubularin-related protein 2 | 0.88±0.17 |
| Mtmr5 | Myotubularin-related protein 5 | 1.11±0.08 |
| Phka2 | Phosphorylase b kinase regulatory subunit alpha, liver isoform | 1.13±0.18 |
| Pik3c2a | Phosphatidylinositol-4-phosphate 3-kinase C2 domain-containing alpha polypeptide | 0.98±0.09 |
| Pip5k1a | Phosphatidylinositol-4-phosphate 5-kinase type-1 beta | 0.72±0.01 |
| Pip5k3 | FYVE finger-containing phosphoinositide kinase | 0.94±0.17 |
| Pkn1 | Serine/threonine-protein kinase N1 | 0.96±0.12 |
| Pkn2 | Serine/threonine-protein kinase N2 | 1.02±0.13 |
| Prkacb | Protein kinase, cAMP dependent, catalytic, beta | 1.18±0.19 |
| Prkag2 | 5'-AMP-activated protein kinase subunit gamma-2 | 1.12±0.09 |
| Prkar1a | Protein kinase, cAMP dependent regulatory, type I, alpha | 1.01±0.28 |
| Prkcd | Protein kinase C delta | 0.93±0.08 |
| Prkd2 | Serine/threonine-protein kinase D2 | 1.36±0.15 |
| Ptpn22 | Tyrosine-protein phosphatase non-receptor type 22 | 1.00±0.06 |
| Raf1 | RAF proto-oncogene serine/threonine-protein kinase | 0.70±0.11 |
| Ripk2 | Receptor-interacting serine/threonine-protein kinase 2 | 1.23±0.14 |
| Rps6ka2 | Ribosomal protein S6 kinase alpha-5 | 1.42±0.13 |
| Rps6kc1 | Ribosomal protein S6 kinase delta-1 | 1.53±0.08 |
| Stk11 | Serine/threonine kinase 11 | 0.66±0.01 |
| Tlk1 | Serine/threonine-protein kinase tousled-like 1 | 1.29±0.08 |
| Tlk2 | Serine/threonine-protein kinase tousled-like 2 | 0.61±0.18 |
| Ulk1 | Serine/threonine-protein kinase ULK1 | 1.00±0.21 |
