# Supplementary material for: LKB1‐MARK2 signalling mediates lipopolysaccharide‐induced production of cytokines in mouse macrophages
Source: J Cell Mol Med. 2020 Aug 25;24(19):11307–17. doi: 10.1111/jcmm.15710 (PMC7576310; doi:10.1111/jcmm.15710)
Supplement: Supplementary file 6 — Table S4 [file JCMM-24-11307-s006.doc]

**Supplemental Table 4.** RT-PCR results for siRNA and luciferase reporter assay transfection

| **Gene** | **Protein** | **Fold change** |
| --- | --- | --- |
| Braf | Serine/threonine-protein kinase B-raf | 0.20±0.12 |
| Btk | Tyrosine-protein kinase BTK | 0.30±0.11 |
| Cdk1 | Cyclin-dependent kinase 1 | 0.31±0.14 |
| Cdk5 | Cyclin-dependent kinase 5 | 0.32±0.10 |
| Cdk11B | Cyclin-dependent kinase 11B | 0.32±0.15 |
| Cdk13 | Cyclin-dependent kinase 13 | 0.29±0.14 |
| Csnk1d | Casein kinase I isoform delta | 0.26±0.15 |
| Dyrk1a | Dual specificity tyrosine-phosphorylation-regulated kinase 1A | 0.26±0.09 |
| Epha10 | Ephrin type-A receptor 10 | 0.42±0.10 |
| Gprk6 | G protein-coupled receptor kinase 6 | 0.33±0.13 |
| Inpp5d | Inositol polyphosphate-5-phosphatase D | 0.30±0.16 |
| Irak3 | Interleukin-1 receptor-associated kinase 3 | 0.27±0.11 |
| Itpkb | inositol 1,4,5-trisphosphate 3-kinase B | 0.26±0.13 |
| Map3k1 | Mitogen-activated protein kinase kinase kinase 1 | 0.25±0.10 |
| Map3k3 | Mitogen-activated protein kinase kinase kinase 3 | 0.25±0.12 |
| Map3k4 | Mitogen-activated protein kinase kinase kinase 4 | 0.18±0.15 |
| Map3k7 | TAK1, Mitogen-activated protein kinase kinase kinase 7 | 0.29±0.07 |
| Map3k20 | Mitogen-activated protein kinase kinase kinase 20 | 0.29±0.12 |
| Map4k1 | Mitogen-activated protein kinase kinase kinase kinase 1 | 0.25±0.11 |
| Mark2 | Serine/threonine-protein kinase MARK2 | 0.23±0.08 |
| Mast3 | Microtubule-associated serine/threonine-protein kinase 3 | 0.24±0.07 |
| Mastl | Microtubule-associated serine/threonine-protein kinase-like | 0.19±0.17 |
| Melk | Maternal embryonic leucine zipper kinase | 0.30±0.15 |
| Mtmr2 | Myotubularin-related protein 2 | 0.32±0.11 |
| Mtmr5 | Myotubularin-related protein 5 | 0.29±0.15 |
| Phka2 | Phosphorylase b kinase regulatory subunit alpha, liver isoform | 0.38±0.10 |
| Pik3c2a | Phosphatidylinositol-4-phosphate 3-kinase C2 domain-containing alpha polypeptide | 0.18±0.13 |
| Pip5k1a | Phosphatidylinositol-4-phosphate 5-kinase type-1 beta | 0.33±0.11 |
| Pip5k3 | FYVE finger-containing phosphoinositide kinase | 0.27±0.12 |
| Pkn1 | Serine/threonine-protein kinase N1 | 0.22±0.15 |
| Pkn2 | Serine/threonine-protein kinase N2 | 0.23±0.09 |
| Prkacb | Protein kinase, cAMP dependent, catalytic, beta | 0.29±0.16 |
| Prkag2 | 5'-AMP-activated protein kinase subunit gamma-2 | 0.30±0.13 |
| Prkar1a | Protein kinase, cAMP dependent regulatory, type I, alpha | 0.34±0.11 |
| Prkcd | Protein kinase C delta | 0.36±0.09 |
| Ptpn22 | Tyrosine-protein phosphatase non-receptor type 22 | 0.26±0.08 |
| Raf1 | RAF proto-oncogene serine/threonine-protein kinase | 0.28±0.18 |
| Ripk2 | Receptor-interacting serine/threonine-protein kinase 2 | 0.34±0.07 |
| Rps6kb2 | Ribosomal protein S6 kinase beta-5 | 0.27±0.09 |
| Rps6kc1 | Ribosomal protein S6 kinase delta-1 | 0.25±0.13 |
| Stk11 | Serine/threonine kinase 11 | 0.24±0.10 |
| Tlk1 | Serine/threonine-protein kinase tousled-like 1 | 0.35±0.10 |
| Tlk2 | Serine/threonine-protein kinase tousled-like 2 | 0.33±0.15 |
| Ulk1 | Serine/threonine-protein kinase ULK1 | 0.37±0.11 |
| Cd14 | Monocyte differentiation antigen CD14 | 2.95±0.98 |
| Md2 | Lymphocyte antigen 96, Ly-96 | 3.86±1.56 |
| Tlr4 | Toll-like receptor 4 | 3.54±1.80 |
